# Global burden of type 2 diabetes in adolescents from 1990 to 2019

**DOI:** 10.3389/fendo.2024.1405739

**Published:** 2024-07-11

**Authors:** Juan Luo, Jiahui Hou, Jinglin Yi, Langbo Li, Xinlan Zhao

**Affiliations:** Department of Endocrinology, Hunan Provincial People’s Hospital, The First Affiliated Hospital of Hunan Normal University, Changsha, China

**Keywords:** type 2 diabetes, adolescents, global burden, incidence, disability-adjusted life years (DALYs), estimated annual percentage change (EAPC)

## Abstract

**Purpose:**

To evaluate the burden of type 2 diabetes (T2D) among adolescents (15–24 years old) from 1990 to 2019.

**Methods:**

The age-standardized incidence rate (ASIR) and disability-adjusted life years (DALYs) rate of adolescents were analyzed according to age, sex, geographical location, and sociodemographic index (SDI). The estimated annual percentage change (EAPC) was estimated to quantify the trends.

**Results:**

From 1990 to 2019, the ASIR (EAPC = 1.07) and age-standardized DALY rate (EAPC = 2.01) of T2D in adolescents showed an increasing trend. The ASIR was higher in males than in females. The burden was greater in the 20–24-year age group. Of the five SDI regions, the highest ASIR and age-standardized DALY rate were found in low-middle-SDI regions, while the greatest increase in these rates was observed in high-SDI regions (EAPC = 3.28 and 3.55, respectively). Of the 21 regions analyzed, the highest ASIR and age-standardized DALY rate were found in Oceania. Of the 204 countries analyzed, the Marshall Islands (651.16) and Kiribati (277.42) had the highest ASIR and DALYs, respectively. The regions with the greatest increase in the ASIR from 1990 to 2019 were Western Europe (EAPC = 4.15), high-income North America (EAPC = 4.72).

**Conclusions:**

The global burden of T2D in adolescents showed an overall upward trend from 1990 to 2019. It is necessary to strengthen prevention measures related to risk factors for T2D among young people, especially in areas with a low-to-medium SDI.

## 1 Introduction

Diabetes is a chronic noninfectious metabolic disease characterized by a high blood glucose concentration. Type 2 diabetes (T2D) is the most common type of diabetes. It accounts for more than 90% of all diagnosed cases of diabetes globally (1) and poses a serious threat to global health (2). In 2019, the number of disability-adjusted life years (DALYs) attributed to diabetes globally was 66.3 million, which makes diabetes the fourth largest cause of disability (3). The global number of diabetes patients is showing a sustained, rapidly increasing trend. In 2019, the global number of diabetes patients was estimated to be 463 million, accounting for 9.3% of the global adult population. By 2030 and 2045, this number is expected to increase to 590 million (10.2%) and 700 million (10.9%) (4, 5), respectively, which will place considerable economic pressure on individuals, families, healthcare systems, and society.

Traditionally, T2D has been considered to be a metabolic disorder in middle-aged and older adults but relatively rare in adolescents and young people. However, due to obesity, a high-energy diet, and a sedentary lifestyle, the incidence rate of T2D in adolescents is increasing sharply. Early-onset T2D (diagnosed before the age of 40, i.e., in adolescents aged 15–24 years) is becoming increasingly common and thus deserves special attention. A study in the United States found that children and adolescents are the age groups with the highest T2D incidence rates (6). From 2002–2003 to 2011–2012, the T2D incidence rate in adolescents aged 10–19 years in the United States increased by 4.8% annually (7). Young people with early-onset T2D are exposed to hyperglycemia for longer periods of time than those with late-onset T2D, which increases their risk of microvascular and macrovascular diseases, kidney disease, and other complications (8). The complex manifestations of T2D in patients aged 15–24 years may require decades of intensive treatment (9). Therefore, understanding the global burden of T2D in adolescents (15–24 years old) may facilitate the balancing of medical resources in different regions and provide the basis and guidance for the prevention and treatment of diabetes.

The data used in this study were derived from the Global Burden of Disease (GBD) Study. The data describe the epidemiological characteristics (incidence rate and DALYs) and global burden of T2D in those aged 15–24 years from 1990 to 2019. We compared the global burden for different ages, sexes, regions, sociodemographic index (SDI) values, and countries. Moreover, we evaluated the trend in the burden of T2D from 1990 to 2019 at the global, regional, and national levels, with the aim of formulating relevant strategies to address these trends.

## 2 Methods

### 2.1 Data source

The data were sourced from the GBD 2019 database published on the website of the Institute for Health Indicators and Evaluation at the University of Washington. The aim of the GBD Study was to assess the global disease burden and health-related data for 369 diseases and injuries and 87 risk factors in 204 countries and regions. We only collected T2D data from those aged 15–24 years, using the Global Health Data Exchange query tool (http://ghdx.healthdata.org/gbd-results-tool). We obtained the incidence rate and DALY data for T2D in adolescents (15–24 years old) according to age, sex, and region. In addition, considering that there is only a small number of T2D patients aged less than 15 years, we selected those over the age of 15 years and divided them into two age groups (15–19 and 20–24 years) for analysis. This study is based on a publicly available database and does not require ethical approval.

The SDI is a comprehensive indicator of per capita income, average educational level, and total fertility rate, with values ranging from 0 to 1. It divides 204 countries into the following five development levels: low SDI (≤ 0.45), low-middle SDI (0.45–0.60), middle SDI (0.60–0.68), high-middle SDI (0.68–0.80), and high SDI (0.80) (9).

### 2.2 T2D definition

According to the 10th edition of the International Classification of Diseases, the T2D codes are E11–E11.1 and the diagnosis codes are E11.3–E11.9. In the 2019 GDB Study, T2D was defined as a fasting plasma glucose concentration ≥ 126 mg/dL (70 mmoL/L), a 2-h blood glucose concentration ≥ 200 mg/dL (11.1 mmoL/L), and/or a record of diabetes treatment.

### 2.3 Statistical analysis

An age-standardized rate per 100,000 population is calculated as follows:


$$\text{Age}-\text{standardized}\;\text{rate}\;=\frac{\sum_{\text{i}=1}^{A}a1wi}{\sum_{i=1}^{A}Wi}\times \;100,000$$

where a_i_ is the age-specific rate for the ith age group, w_i_ is the number of people in the corresponding ith age subgroup in the selected reference standard population, and A is the number of age groups.

Age standardization aims to eliminate the impact of population age composition and ensure comparability of research indicators. The age-standardized rate in the GBD database is estimated using the world-population age standard (10). Direct standardization results in standardized or age-adjusted rates, which are weighted averages of specific age rates for each population being compared. The weight (standard) is used to represent the relative age distribution of an external population and serves as a summary rate for each population. This rate reflects the number of events that can be expected to occur if the populations being compared have the same age distribution.

EAPC is a widely accepted quantitative indicator used for estimating the annual average change in age-standardized rate within a given period. Fitting was performed based on the natural logarithm of time variables and their corresponding observations, thereby ensuring that each observation contributed to the calculation of EAPCs. Moreover, as EAPC estimates and quantifies the long-term trend in disease burden indicators such as the incidence rate and mortality of diseases.

We established a regression model to describe the relationship between the natural logarithm (ln) of age-standardized rate and time, as defined by y=b0+βx+c, y=In(ASIR), where x=the calendar year, b0 is constant term, c is the false term, and β is the meaning of the negative or positive tendency of the selected age-standardized rate. The EAPC was counted using the following formula: EAPC = 100 × (exp [β] − 1). The 95% confidence interval (CI) of the EAPC was obtained from the linear regression model (11).

If the EAPC and its 95% CI were less than 0, it indicated a downward trend, whereas if the EAPC and its 95% CI were greater than 0, it indicated an upward trend. R software (version 4.1.1) was used for the statistical analyses.

## 3 Results

### 3.1 Global burden and trend analysis

**Figure 1:**
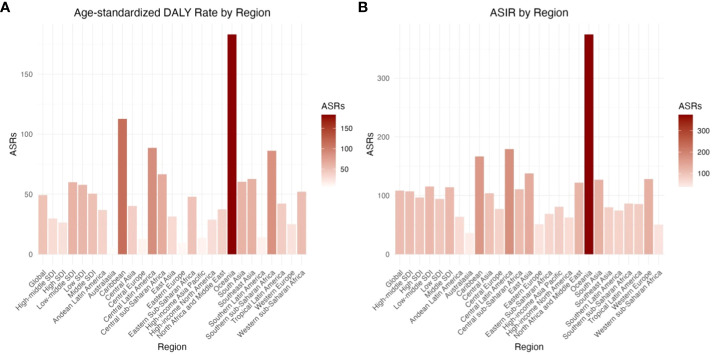
The age-standardized DALY rate and the age-standardized incidence rate (ASIR) of type 2 diabetes among adolescents by 21 regions in 2019. **(A)** Age-standardized DALY Rate by Region **(B)** ASIR by Region.

In 1990, the global age-standardized incidence rate (ASIR) for T2D in adolescents (15–24 years old) from the ages of 15–24 years was 70.05 (95% UI: 69.89–70.21) per 100,000 people, and the age-standardized DALY rate was 37.02 (95% UI: 36.90–37.13) per 100,000 people. In 2019, the ASIR for adolescents (15–24 years old) from the ages of 15–24 years was 108.36 (95% UI: 108.17–108.54) per 100,000 people, and the age-standardized DALY rate was 49.20 (95% UI: 49.07–49.32) per 100,000 people. This represents a 38.31% and 12.18% increase in the ASIR and age-standardized DALY rate, respectively, from 1990 to 2019 (**Table 1**; **Figure 1**).

**Table 1:** The age-standardized DALY rate and the age-standardized incidence rate (ASIR) of type 2 diabetes among adolescents in 1990 and 2019, and its temporal trends from 1990 to 2019.

Location	1990				2019				1990–2019	1990–2019
	Age-standardized DALY rate (per 100000)		ASIR		Age-standardized DALY rate (per 100000)		ASIR		EAPC DALY	EAPC incidence
	No.(95%UI)	Male/ Female	No.(95%UI)	Male/ Female	No.(95%UI)	Male/ Female	No.(95%UI)	Male/ Female	No.(95%CI)	No.(95%CI)
Global	37.02(36.90,37.13)	0.77	70.05(69.89,70.21)	1.07	49.20(49.07,49.32)	0.87	108.36 (108.17,108.54)	1.11	1.07(1.00,1.14)	2.01(1.85,2.17)
Sociodemographic index	–	–	–	–	–	–	–	–	–	–
High-middle SDI	25.79(25.58,26.01)	0.82	71.06(70.70,71.43)	1.12	29.83(29.57,30.08)	0.99	107.14(106.66,107.62)	1.20	1.47(1.15,1.78)	2.70(2.30,3.10)
High SDI	11.78(11.60,11.97)	0.69	46.08(45.71,46.45)	1.03	26.44(26.16,26.73)	0.86	96.94(96.38,97.49)	1.03	3.55(3.28,3.82)	3.28(3.05,3.51)
Low-middle SDI	46.41(46.13,46.70)	0.68	70.53(70.18,70.88)	1.02	60.00(59.74,60.27)	0.82	115.43(115.06,115.79)	1.08	0.67(0.55,0.78)	1.56(1.43,1.69)
Low SDI	51.96(51.50,52.41)	0.85	61.80(61.31,62.30)	1.03	57.79(57.48,58.11)	0.86	94.38(93.98,94.78)	1.07	0.17(0.08,0.26)	1.21(1.09,1.33)
Middle SDI	42.36(42.15,42.57)	0.80	79.56(79.27,79.85)	1.10	50.41(50.18,50.64)	0.93	114.16(113.82,114.51)	1.14	0.77(0.68,0.86)	2.02(1.78,2.27)
Region	–	–	–	–	–	–	–	–	–	–
Andean Latin America	25.45(24.33,26.61)	0.65	34.00(32.70,35.34)	0.96	36.89(35.77,38.03)	0.83	63.91(62.43,65.41)	0.97	1.12(0.96,1.27)	1.88(1.76,1.99)
Australasia	4.10(3.43,4.85)	0.71	17.08(15.70,18.55)	0.87	5.98(5.22,6.82)	0.55	36.32(34.40,38.32)	0.82	1.03(0.48,1.58)	2.16(1.71,2.62)
Caribbean	94.40(92.15,96.69)	0.38	110.24(107.81,112.72)	0.75	112.77(110.41,115.17)	0.49	166.77(163.89,169.69)	0.73	0.54(0.44,0.65)	1.28(1.21,1.34)
Central Asia	28.43(27.50,29.38)	0.59	65.52(64.11,66.95)	0.86	40.24(39.23,41.27)	0.54	103.88(102.25,105.53)	0.82	0.80(0.55,1.04)	1.72(1.57,1.88)
Central Europe	12.64(12.12,13.16)	0.68	51.64(50.60,52.70)	1.12	12.84(12.22,13.49)	0.87	77.22(75.67,78.79)	1.13	0.18(-0.01,0.38)	1.34(1.14,1.54)
Central Latin America	78.41(77.46,79.36)	0.66	114.27(113.14,115.42)	0.97	88.66(87.78,89.54)	0.76	179.29(178.04,180.55)	0.88	0.42(0.24,0.60)	1.70(1.53,1.86)
Central sub-Saharan Africa	66.10(64.55,67.69)	1.36	71.38(69.77,73.02)	1.29	66.62(65.62,67.63)	1.32	110.64(109.35,111.93)	1.34	-0.02(-0.06,0.03)	1.43(1.32,1.55)
East Asia	25.87(25.68,26.07)	1.10	93.29(92.93,93.66)	1.26	31.48(31.22,31.75)	1.40	137.72(137.15,138.28)	1.53	2.81(2.21,3.41)	3.56(2.90,4.22)
Eastern Europe	12.37(11.98,12.77)	0.79	48.09(47.32,48.87)	0.99	9.60(9.19,10.03)	1.05	51.26(50.29,52.24)	1.09	-1.47(-1.77,-1.18)	-0.06(-0.22,0.10)
Eastern Sub-Saharan Africa	57.42(56.64,58.21)	1.07	54.97(54.21,55.74)	1.07	47.93(47.47,48.40)	1.23	68.91(68.36,69.47)	1.21	-0.87(-0.95,-0.78)	0.65(0.60,0.69)
High-income Asia Pacific	7.54(7.23,7.86)	0.50	46.38(45.60,47.17)	1.21	13.74(13.23,14.26)	0.93	81.03(79.76,82.32)	1.17	2.01(1.82,2.20)	1.99(1.83,2.15)
High-income North America	8.22(7.95,8.49)	0.56	36.70(36.12,37.30)	0.84	28.78(28.30,29.27)	0.78	62.67(61.96,63.39)	0.74	4.72(4.38,5.05)	2.29(2.07,2.51)
North Africa and Middle East	30.63(30.21,31.05)	0.53	59.43(58.84,60.01)	0.93	37.49(37.12,37.86)	0.70	121.95(121.29,122.62)	1.01	0.76(0.69,0.82)	2.64(2.60,2.69)
Oceania	115.85(110.01,121.91)	1.26	218.12(210.10,226.37)	1.55	183.20(177.94,188.58)	1.23	374.88(367.33,382.54)	1.47	1.40(1.20,1.60)	1.82(1.71,1.92)
South Asia	45.25(44.97,45.55)	0.60	75.33(74.96,75.71)	0.98	60.33(60.07,60.59)	0.74	126.97(126.60,127.35)	1.06	0.58(0.40,0.77)	1.32(1.07,1.57)
Southeast Asia	66.54(66.01,67.06)	0.94	55.83(55.35,56.32)	0.97	62.69(62.22,63.15)	1.05	80.07(79.55,80.59)	1.00	-0.68(-0.95,-0.41)	0.68(0.30,1.07)
Southern Latin America	9.35(8.70,10.02)	0.21	32.71(31.50,33.96)	0.75	14.28(13.57,15.02)	0.47	74.67(73.02,76.35)	0.83	1.41(1.16,1.66)	3.26(3.08,3.44)
Southern sub-Saharan Africa	61.99(60.52,63.49)	0.83	57.10(55.69,58.55)	0.77	86.24(84.70,87.80)	1.44	86.52(84.98,88.08)	0.88	1.40(0.94,1.86)	1.48(1.24,1.72)
Tropical Latin America	59.66(58.79,60.54)	0.60	66.09(65.17,67.02)	0.96	42.26(41.59,42.94)	0.90	85.65(84.69,86.62)	1.07	-1.19(-1.56,-0.81)	1.01(0.87,1.14)
Western Europe	12.57(12.29,12.86)	0.79	47.63(47.07,48.19)	0.97	25.02(24.58,25.47)	0.86	128.14(127.14,129.15)	1.05	3.30(3.03,3.56)	4.15(3.93,4.37)
Western sub-Saharan Africa	42.72(42.05,43.40)	0.82	32.40(31.81,33.00)	0.92	52.08(51.62,52.55)	0.76	50.58(50.11,51.04)	0.99	0.59(0.36,0.82)	1.60(1.52,1.68)

From 1990 to 2019, the ASIR and age-standardized DALY rate of T2D in adolescents aged 15–24 years showed an increasing trend in both males (EAPC = 1.07, 95% CI: 1.00–1.14) and females (EAPC = 2.01, 95% CI: 1.85–2.17) (**Table 1**; **Figure 1**).

### 3.2 Trends according to sex and age

**Figure 2:**
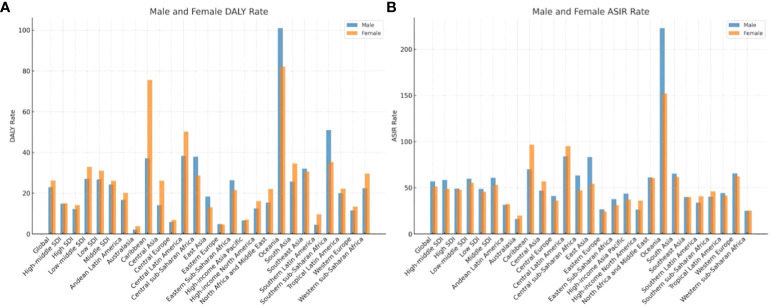
The gender distribution of ASIR and age DALY standardized rate of type 2 diabetes among adolescents in the world, 21 regions and different SDI sub-regions. **(A)** Male and Female DALY Rate **(B)** Male and Female ASIR Rate.

The male-to-female ASIR ratios for T2D in adolescents (15–24 years old) were 1.07 and 1.11 per 100,000 people globally in 1990 and 2019, respectively, indicating that the ASIR was higher in males than in females. This was true for all of the five SDI regions. In 1990 and 2019, the age-standardized DALY rates for adolescents (15–24 years old) were 0.77 and 0.87 for every 100,000 people, respectively. The age-standardized DALY rate was lower in males than in females, and this was true for all of the five SDI regions (**Table 1**; **Figure 2**).

**Figure 3:**
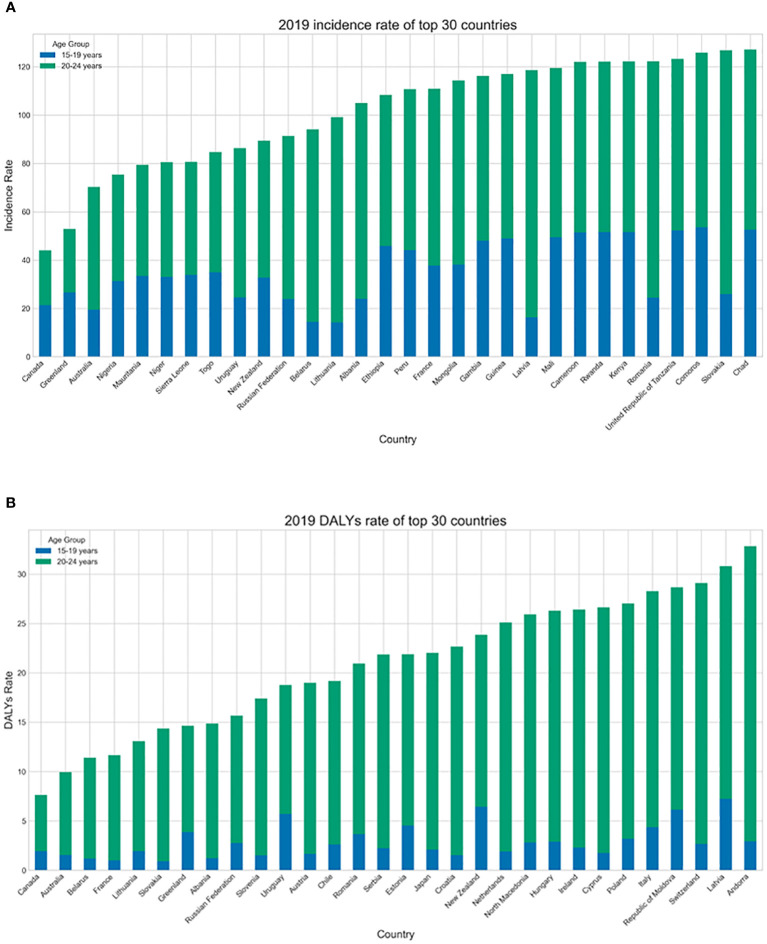
**(A)** The age groups of the highest age-standardized incidence rate (ASIR) of top 30 countries with in 2019. **(B)** The age groups of age DALY standardized rate of top 30 countries with in 2019.

Of the 30 countries with the highest incidence rate and DALY rate in 2019, all except Greenland showed lower incidence rates in the 15–19-year age group than in the 20–24-year age group (**Figures 3A, B**). Only 17 (Benin, Burkina Faso, Cameroon, Chad, Côte dIvoire, Gambia, Ghana, Guinea, Guinea Bissau, Mali, Mauritania, Niger, Nigeria, Senegal, Sierra Leone, Togo, and Zimbabwe) of the 204 countries analyzed had a higher age-standardized DALY rate in the 15–19-year age group, while only two countries (Greenland and United States) had a higher ASIR in the 15–19-year age group (**Supplementary Tables 1**, **2**; **Supplementary Figure 1**).

### 3.3 T2D burden and its trend by SDI

In 2019, the highest ASIR and age-standardized DALY rate for adolescents (15–24 years old) were found in low-middle-SDI regions, with an ASIR of 115.43 (95% UI: 115.06–115.79) and an age-standardized DALY rate of 60.00 (95% UI: 59.74–60.27) for every 100,000 people aged 15–24 years. The ASIR was lowest at 94.38 per 100,000 people (95% UI: 93.98–94.78) in low-SDI regions, and the age-standardized DALY rate was lowest at 26.44 for every 100,000 people (95% UI: 26.16–26.73) in high-SDI regions (**Table 1**; **Figure 1**).

From 1990 to 2019, the ASIR and age-standardized DALY rate increased to a certain degree in all regions. The ASIR (EAPC = 3.28) and age-standardized DALY rate (EAPC = 3.55) both increased the most in high-SDI regions, while the ASIR and age-standardized DALY rate increased the least in low-SDI regions, with growth rates of ASIR(121%)and ge-standardized DALY rate(17%), respectively (**Table 1**; **Figure 1**).

**Figure 4:**
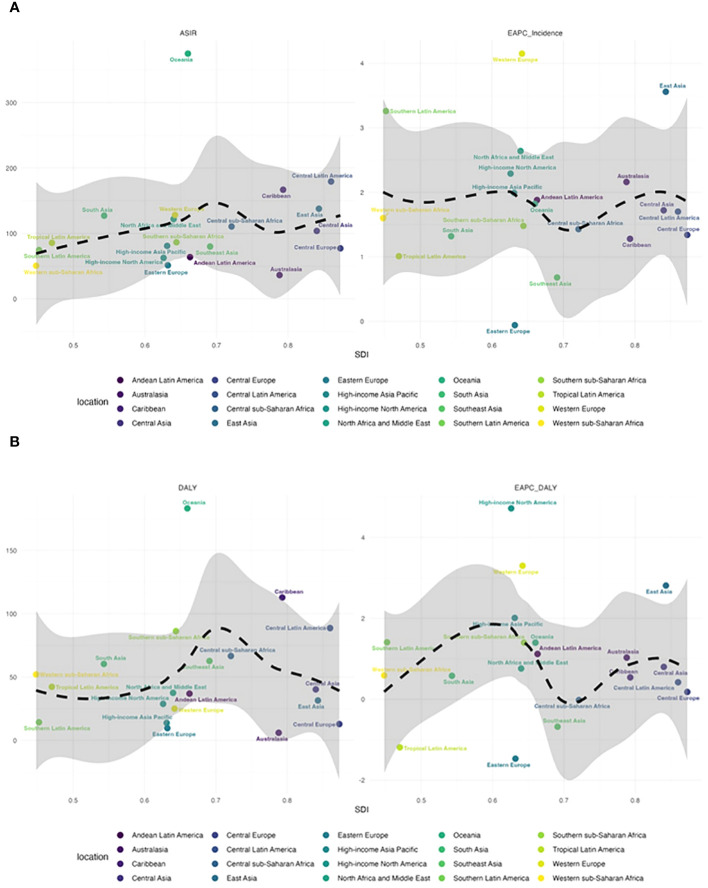
**(A)** The relationship between ASIR and EAPC and SDI in 21 regions. **(B)** The relationship between DALY and EAPC and SDI in 21 regions.

As shown in **Figure 4A**, in 21 regions, the ASIR for T2D in adolescents (15–24 years old) roughly showed a wave-like trend as the SDI value increased, first increasing, then decreasing, and then increasing again. The ASIR reached its highest level at an SDI value of approximately 0.7, and in Oceania, Central Latin America, South Asia, the Caribbean, and East Asia, it far exceeded the expected level. The relationship between the ASIR, EAPC, and SDI was relatively stable, with the EPAC reaching its lowest level at an SDI value of approximately 0.7. In Western Europe, East Asia, and Southern Latin America, the ASIR growth rate was significant and was much higher than expected. The DALYs associated with adolescents (15–24 years old) showed a wave-like trend according to the SDI value, first increasing and then decreasing as the SDI value increased (**Figure 4B**). The ASIR increased until an SDI value of 0.7 and then decreased. The relationship between DALYs, the EAPC, and the SDI value also showed a wave-like pattern, with two peaks at SDI values of approximately 0.6 and 0.83, with the largest increase at an SDI value of 0.6. The DALY rates in high-income North America, Western Europe, and East Asia were much higher than the global expectations.

In all 204 countries analyzed, the ASIR and EAPC for adolescents (15–24 years old) first decreased and then increases as the SDI value increased (**Supplementary Figure 2A**). The ASIR reached its lowest level at an SDI value of approximately 0.75, corresponding to countries such as the Netherlands, Denmark, and Germany. The ASIR showed a continuous downward trend as the SDI value increased to 0.75. Then, as the SDI value increased further above 0.75, most countries showed an increasing trend in the ASIR, with these trends in countries such as Qatar, Guatemala, and Cameroon far exceeding expectations. The relationship between DALYs, the EAPC, and the SDI value showed a similar trend (**Supplementary Figure 2B**).

### 3.4 T2D burden by geographical region

In 2019, of the 21 geographical regions analyzed, Oceania (374.88, 95% UI: 367.33–382.54) had the highest ASIR per 100,000 people for adolescents (15–24 years old), followed by Central Latin America (179.29, 95% UI: 178.04–180.55) and the Caribbean (166.77, 95% UI: 163.89–169.69). The three regions with the lowest ASIR were Australasia (36.32, 95% UI: 34.40–38.32), Western Sub-Saharan Africa (50.58, 95% UI: 50.11–51.04), and Eastern Europe (51.26, 95% UI: 50.29–52.24). From 1990 to 2019, the ASIR showed the greatest increase in Western Europe (EAPC = 4.15, 95% CI: 3.93–4.37) and the greatest decrease in Eastern Europe (EAPC = -0.06, 95% CI: -0.22–0.10; **Supplementary Table 6**; **Figures 1A**, **4A**; **Supplementary Figure 3**).

In 2019, the regions with the highest age-standardized DALY rate per 100,000 people for T2D in adolescents (15–24 years old) were Oceania (183.20, 95% UI: 177.94–188.58), Caribbean (112.77, 95% UI: 110.41–115.17), and Central Latin America (88.66, 95% UI: 87.78–89.54), while the regions with the lowest age-standardized DALY rate were Australasia (5.98, 95% UI: 5.22–6.82), Eastern Europe (9.60 95% UI: 9.19–10.03), and Central Europe (12.84, 95% UI: 12.22–13.49). From 1990 to 2019, the age-standardized DALY rate showed the largest increase in high-income North America (EAPC = 4.72, 95% CI: 4.38–5.05), followed by Western Europe (EAPC = 3.30, 95% CI: 3.03–3.56) and East Asia (EAPC = 2.81, 95% CI: 2.21–3.41). Eastern Europe (EAPC = -1.47, 95% CI: -1.77 to -1.18), showed the greatest decrease, followed by Tropical Latin America (EAPC = -1.19, 95% CI: -1.56 to -0.81) and Eastern Sub-Saharan Africa (EAPC = -0.87, 95% CI: -0.95 to -0.78; **Supplementary Table 3**; **Figures 1B**, **4B**; **Supplementary Figure 4**)

### 3.5 T2D burden by country

In 2019, of the 204 countries analyzed, the Marshall Islands (651.16, 95% UI: 503.74–828.54), American Samoa (623.60, 95% UI: 483.66–791.91), and Niue (531.10, 95% UI: 27.78–2528.02) had the highest ASIR per 100,000 people for T2D in adolescents (15–24 years old), and in these countries, the DALY rate was higher in the 15–19-year age group than the 20–24-year age group. Canada (22.00, 95% UI: 20.60–23.46), Greenland (26.45, 95% UI: 3.26–98.02), and Australia (34.72, 95% UI: 32.68–36.86) had the lowest ASIR, and from 1990 to 2019, the ASIR increased the most in the United Kingdom (EAPC = 7.72, 95% CI: 6.53–8.93), followed by Greenland (EAPC = 4.98, 95% CI: 4.65–5.30) and Uruguay (EAPC = 4.41, 95% CI: 3.89–4.94). The ASIR showed the largest decrease in Ethiopia (EAPC = -1.04, 95% CI: -1.11 to -0.96), followed by Rwanda (EAPC = -0.74, 95% CI: -0.99 to -0.48) and Belarus (EAPC = -0.41, 95% CI: -0.63 to -0.18; **Supplementary Table 4**; **Supplementary Figures 1A**, **2A**, **3**).

In 2019, Kiribati (277.42, 95% UI: 211.87–356.90), the Solomon Islands (261.18, 95% UI: 233.34–291.45), and the Marshall Islands (207.13, 95% UI: 128.08–317.14) had the highest age-standardized DALY rates per 100,000 individuals for T2D in adolescents (15–24 years old). Canada (3.76, 95% UI: 3.21–4.38), Australia (4.88, 95% UI: 4.14–5.72), and Belarus (5.59, 95% UI: 4.18–7.33) had the lowest age-standardized DALY rates. From 1990 to 2019, the age-standardized DALY rate showed the greatest increase in Mauritius (EAPC = 4.21, 95% CI: 3.65–4.78), followed by the United States (EAPC = 4.73, 95% CI: 4.38–5.07) and the United Kingdom (EAPC = 5.96, 95% CI: 4.76–7.18). The age-standardized DALY rate showed the greatest decrease in Ethiopia (EAPC = -3.32, 95% CI: -3.46 to -3.17), followed by Belarus (EAPC = -3.25, 95% CI: -3.73 to -2.76) and the Philippines (EAPC = -3.06, 95% CI: -3.48 to -2.64; **Supplementary Tables 3**, **5**; **Supplementary Figures 1B**, **2B**, **4**).

## 4 Discussion

Our analysis of data from the 2019 GBD Study showed that, from 1990 to 2019, the burden of T2D in adolescents (15–24 years old) showed an upward trend, with the greatest burden in medium-low-SDI regions and the fastest growth in high-SDI regions. Males were more likely to suffer from T2D, while females showed a higher number of DALYs associated with T2D. The etiology of T2D is complex, and in addition to being influenced by known immutable factors, such as age and genetic factors, there are some factors that can be modified through lifestyle changes. In recent years, the surge in T2D cases has been largely due to lifestyle changes and rapid economic development, continuous urbanization, sedentary lifestyles, and unhealthy dietary patterns are considered to be the main factors driving this growth (12).

Obesity and overweight are recognized as the main risk factors for T2D. In 2016, it was estimated that more than 80% of adolescents and approximately 27.5% of adults globally did not meet the recommended physical activity levels (13). High-energy foods and high-sugar beverages, as well as reduced levels of physical activity, are the main causes of obesity in adolescents and young adults (14). A high body mass index is closely related to insulin resistance, dyslipidemia, and other cardiac metabolic risk factors. The associations between physical activity and sedentary time and the risk of T2D are largely mediated by obesity. Research has shown that the prevalence of obesity among adolescents and young adults has increased globally. Therefore, controlling weight in adolescents is key to reducing the incidence of adolescents (15–24 years old).

A poor diet, with high levels of processed and red meat and low levels of fruit and whole grains, is another risk factor for T2D (15). The main reason for obesity in young people is the consumption of high-energy foods and high-sugar beverages. The advertising of fast food, carbonated drinks, and sugary beverages targeting adolescents has increased, and the consumption of these foods and drinks has increased accordingly (15). According to GBD data, in 2013, overweight and obesity affected 24% of boys and 23% of girls (aged less than 20 years) globally. Dietary quality is the main driving factor for adolescent obesity and the T2D epidemic (15).

The burden of T2D was greater in the 20–24-year age group than in the 15–19-year age group. In late adolescence and early adulthood, significant biological and social changes occur. The sharp increase in risk factors, such as alcohol consumption, smoking, overweight, and unsafe sexual behavior in early adulthood, coupled with longer exposure to pollutants, may lead to the age-related heterogeneity in the burden of T2D (16). Although the absolute burden of T2D remains small for adolescents, related complications may seriously shorten life expectancy and reduce the quality of life. According to a previous research model, after the diagnosis of diabetes at the age of 20 years, the life expectancy of females decreases by 17.9 years and the life expectancy of males decreases by 17.2 years (17). Besides, a diagnosis of T2D between the ages of 19 and 24 years can significantly increase medical and socioeconomic costs. Therefore, when developing preventive measures for adolescents, the importance of sex and age differences should be taken into account, and more attention should be paid to the differences in risk factors among high-risk youth groups.

We found that the incidence of T2D was higher in males than in females. This may be because males have greater levels of exposure to smoking, alcohol consumption, dietary salt intake, cholesterol, stress, and other risk factors (17). Although the global smoking rate is declining, there is a correlation between smoking and the incidence of diabetes, and the burden attributed to smoking remains large (18). The dietary habits of males, including a high intake of red meat and processed meat and a low intake of fruits and whole grains, may also explain the observed sex differences (19). However, the number of DALYs was higher in females than in males. This may be because females enter puberty earlier than males, and this is associated with fat accumulation. Among all age groups, the global prevalence of obesity is generally higher in females than in males. Compared with boys and males, girls and females undertake less exercise, whereas females engage in more household activities (13). A high body mass index and low levels of physical activity have a greater impact on the adolescents (15–24 years old) in females than in males. The proportion of females exposed to second-hand smoke is 12.30%, while in males, it is 6.37% (10). In underdeveloped areas, it is more difficult for girls to receive diagnosis, treatment, and metabolic health prevention and control interventions, such as effective control of blood glucose concentrations, leading to disability and serious complications (20).

Consistent with the findings of previous studies, the burden of T2D was mainly concentrated in high-income countries (21). We also found that the ASIR and age-standardized DALY rate of 15–24-year-olds showed an upward trend in all of the five SDI regions. The increase in the T2D burden was 2–3 times greater in high-income countries, such as Western Europe, high-income North America, the United States, and the United Kingdom, than in low-income countries. The Western dietary pattern of consuming large amounts of red and processed meat, refined carbohydrates, and sugary beverages is closely related to the risk of T2D (22). Meta-analyses have consistently suggested that a diet rich in processed meat is associated with an increased risk of T2D in high-income countries (23). The increase in obesity incidence may be a result of economic development and may affect the occurrence of T2D, but economic development and wealth can also promote better interventions for T2D.

However, the ASIR and age-standardized DALY rate of T2D were significantly higher in low- and middle-income regions, such as Oceania, the Caribbean, and Central Latin America, and countries, such as the Marshall Islands, Kiribati, and the Solomon Islands, than in other regions. Approximately 80% of global T2D cases occur in low-income and low-to-middle-income countries. It is predicted that the most rapid increase in the number of T2D cases will continue to occur in these regions in the coming decades (18). Malnutrition was once rampant in these countries, but now there is surplus nutrition. This nutritional transformation includes increased consumption of high-energy, low-nutrient, highly processed foods, such as refined grains, food with added sugar and sodium, animal fats, and trans fats, leading to a decrease in the consumption of whole grains, fruits, and vegetables (24). This dietary pattern has been adopted by low- and middle-income countries, and risk factors occurring early in life (famine, malnutrition, and low birth weight), as well as current levels of malnutrition and environmental pollution exposure, are important factors contributing to the rapid increase in the incidence of T2D in low- and middle-income countries. It is estimated that 3 billion people in low- and middle-income countries still use solid fuels and traditional stoves for heating and cooking, which increases their exposure to pollution, leading to T2D and its complications (18). The incidence rate and severity of early-onset diabetes continue to increase, and the burden gap between groups with different income levels may further expand in the future. In addition, the limited health resources for the prevention and treatment of diabetes in low-income countries will bring serious public health challenges.

Based on data from the 2019 GDB database, here we report the trends in T2D burden among 15–24-year-olds in 204 countries and 21 regions from 1990 to 2019. A comprehensive evaluation was conducted, but due to limitations in GBD research methods, age-standardized CIs were used to estimate the annual percentage change to reduce bias and inaccuracy. However, bias cannot be completely excluded, the analysis is based on age-standardized rates, which may obscure variations in the burden of T2D within specific age groups or populations. Therefore, it is important to interpret these results with caution. The incidence rate and DALY data for adolescents (15–24 years old) are influenced by factors such as detection methods, screening quality, and data registration in each region. Discrepancies in data quality and completeness across regions can lead to underestimations, particularly in countries with a low SDI value. The age threshold for T2D is set at fifteen years, and due to insufficient data, children and adolescents younger than 15 years were not included in the analysis as they were unlikely to impact the results. Furthermore, the discussion on risk factors primarily focuses on lifestyle factors like diet and physical activity, while other potential contributors to the burden of T2D, such as genetic predisposition and environmental factors, are given less emphasis.

The global burden of T2D among adolescents (aged 15–24 years) showed an overall upward trend from 1990 to 2019; however, there were significant differences according to sex, age, and region. Low-to-medium-SDI regions, such as Oceania, the Marshall Islands, and Kiribati, showed the highest burden. Areas with a high SDI value, such as high-income North America, Western Europe, and the United Kingdom, showed the most rapid increase in the burden of T2D. Thus, it is necessary to strengthen prevention, control, and intervention measures related to risk factors for T2D in adolescents, especially in areas with a low-to-medium SDI value.

## Data availability statement

The original contributions presented in the study are included in the article/**Supplementary Material**. Further inquiries can be directed to the corresponding author.

## Ethics statement

Ethical approval was not required for the studies involving humans because the data is publicly available. The studies were conducted in accordance with the local legislation and institutional requirements. Written informed consent for participation was not required from the participants or the participants’ legal guardians/next of kin in accordance with the national legislation and institutional requirements because the data is publicly available.

## Author contributions

JL: Writing – original draft, Writing – review & editing. JH: Writing – original draft, Writing – review & editing. JY: Writing – original draft, Writing – review & editing. LL: Writing – original draft, Writing – review & editing. XZ: Writing – original draft, Writing – review & editing.
