# Supplementary material for: Global burden of type 2 diabetes in adolescents from 1990 to 2019
Source: Front Endocrinol (Lausanne). 2024 Jul 11;15:1405739. doi: 10.3389/fendo.2024.1405739 (PMC11269148; doi:10.3389/fendo.2024.1405739)
Supplement: Supplementary file 1 [file DataSheet_1.docx]

**Supplementary table 1. Age distribution of the DALY rate of type 2 diabetes among adolescents in 204 countries.**

| 2019 daly rate | 15-19 years | 20-24 years |
| --- | --- | --- |
| Afghanistan | 33.06 | 100.50 |
| Albania | 1.23 | 13.64 |
| Algeria | 13.12 | 48.98 |
| American Samoa | 84.99 | 290.93 |
| Andorra | 2.94 | 29.89 |
| Angola | 44.55 | 91.42 |
| Antigua and Barbuda | 65.72 | 131.40 |
| Argentina | 6.63 | 26.82 |
| Armenia | 14.66 | 45.81 |
| Australia | 1.56 | 8.39 |
| Austria | 1.66 | 17.34 |
| Azerbaijan | 24.60 | 61.69 |
| Bahamas | 70.61 | 148.10 |
| Bahrain | 45.41 | 106.67 |
| Bangladesh | 53.84 | 76.84 |
| Barbados | 88.40 | 158.01 |
| Belarus | 1.20 | 10.22 |
| Belgium | 3.06 | 30.99 |
| Belize | 85.42 | 154.52 |
| Benin | 73.44 | 66.30 |
| Bermuda | 32.17 | 68.68 |
| Bhutan | 32.25 | 61.33 |
| Bolivia (Plurinational State of) | 25.61 | 50.17 |
| Bosnia and Herzegovina | 12.48 | 40.12 |
| Botswana | 79.31 | 102.32 |
| Brazil | 26.35 | 59.25 |
| Brunei Darussalam | 35.42 | 101.31 |
| Bulgaria | 6.11 | 28.87 |
| Burkina Faso | 72.08 | 57.88 |
| Burundi | 37.16 | 68.33 |
| Cabo Verde | 53.94 | 55.40 |
| Cambodia | 44.94 | 81.18 |
| Cameroon | 91.95 | 73.61 |
| Canada | 1.95 | 5.67 |
| Central African Republic | 54.90 | 114.47 |
| Chad | 74.74 | 64.02 |
| Chile | 2.61 | 16.56 |
| China | 11.20 | 52.64 |
| Colombia | 42.96 | 93.20 |
| Comoros | 29.19 | 58.99 |
| Congo | 45.08 | 92.94 |
| Cook Islands | 57.68 | 193.62 |
| Costa Rica | 40.76 | 91.66 |
| Croatia | 1.54 | 21.12 |
| Cuba | 45.93 | 101.12 |
| Cyprus | 1.76 | 24.88 |
| Czechia | 3.63 | 32.86 |
| Côte d'Ivoire | 78.60 | 67.64 |
| Democratic People's Republic of Korea | 17.70 | 68.96 |
| Democratic Republic of the Congo | 42.93 | 88.11 |
| Denmark | 4.09 | 29.79 |
| Djibouti | 33.72 | 69.55 |
| Dominica | 102.44 | 199.71 |
| Dominican Republic | 77.68 | 155.64 |
| Ecuador | 35.44 | 70.82 |
| Egypt | 18.00 | 52.32 |
| El Salvador | 50.60 | 108.98 |
| Equatorial Guinea | 45.54 | 95.50 |
| Eritrea | 35.97 | 75.51 |
| Estonia | 4.54 | 17.35 |
| Eswatini | 132.98 | 162.55 |
| Ethiopia | 24.35 | 48.87 |
| Fiji | 129.14 | 288.95 |
| Finland | 13.18 | 62.55 |
| France | 1.00 | 10.66 |
| Gabon | 52.96 | 109.58 |
| Gambia | 62.20 | 50.62 |
| Georgia | 15.69 | 56.91 |
| Germany | 12.50 | 53.59 |
| Ghana | 80.23 | 61.93 |
| Greece | 3.71 | 36.38 |
| Greenland | 3.86 | 10.79 |
| Grenada | 95.51 | 173.93 |
| Guam | 46.01 | 134.16 |
| Guatemala | 73.61 | 150.36 |
| Guinea | 80.44 | 71.11 |
| Guinea-Bissau | 99.77 | 84.77 |
| Guyana | 122.56 | 252.33 |
| Haiti | 96.91 | 183.56 |
| Honduras | 55.71 | 115.07 |
| Hungary | 2.90 | 23.40 |
| Iceland | 4.98 | 35.52 |
| India | 34.35 | 71.44 |
| Indonesia | 51.52 | 86.10 |
| Iran (Islamic Republic of) | 12.88 | 39.20 |
| Iraq | 47.27 | 114.64 |
| Ireland | 2.29 | 24.12 |
| Israel | 5.26 | 34.48 |
| Italy | 4.38 | 23.91 |
| Jamaica | 63.75 | 133.24 |
| Japan | 2.09 | 19.95 |
| Jordan | 17.54 | 54.75 |
| Kazakhstan | 17.85 | 54.85 |
| Kenya | 24.83 | 52.84 |
| Kiribati | 136.79 | 426.09 |
| Kuwait | 22.71 | 81.32 |
| Kyrgyzstan | 13.17 | 37.13 |
| Lao People's Democratic Republic | 59.14 | 105.07 |
| Latvia | 7.24 | 23.56 |
| Lebanon | 13.88 | 53.14 |
| Lesotho | 95.01 | 119.44 |
| Liberia | 71.08 | 76.72 |
| Libya | 12.12 | 55.62 |
| Lithuania | 1.94 | 11.13 |
| Luxembourg | 3.17 | 29.88 |
| Madagascar | 32.60 | 65.10 |
| Malawi | 39.03 | 79.45 |
| Malaysia | 26.72 | 63.01 |
| Maldives | 20.24 | 46.68 |
| Mali | 70.74 | 65.36 |
| Malta | 8.86 | 45.49 |
| Marshall Islands | 98.37 | 322.11 |
| Mauritania | 46.24 | 46.06 |
| Mauritius | 52.85 | 114.07 |
| Mexico | 57.29 | 133.57 |
| Micronesia (Federated States of) | 93.24 | 298.15 |
| Monaco | 3.91 | 33.96 |
| Mongolia | 17.17 | 32.87 |
| Montenegro | 3.66 | 29.43 |
| Morocco | 13.42 | 49.18 |
| Mozambique | 48.05 | 94.94 |
| Myanmar | 59.62 | 111.87 |
| Namibia | 47.27 | 64.80 |
| Nauru | 99.30 | 291.62 |
| Nepal | 56.02 | 68.73 |
| Netherlands | 1.91 | 23.20 |
| New Zealand | 6.44 | 17.42 |
| Nicaragua | 56.20 | 110.45 |
| Niger | 56.82 | 46.64 |
| Nigeria | 37.22 | 29.34 |
| Niue | 97.22 | 296.77 |
| North Macedonia | 2.81 | 23.12 |
| Northern Mariana Islands | 46.97 | 149.92 |
| Norway | 11.21 | 39.95 |
| Oman | 16.07 | 44.86 |
| Pakistan | 76.95 | 131.10 |
| Palau | 75.31 | 253.35 |
| Palestine | 19.43 | 55.06 |
| Panama | 47.94 | 97.12 |
| Papua New Guinea | 100.97 | 265.45 |
| Paraguay | 25.38 | 55.63 |
| Peru | 17.89 | 38.15 |
| Philippines | 52.57 | 93.10 |
| Poland | 3.19 | 23.84 |
| Portugal | 9.46 | 50.03 |
| Puerto Rico | 49.30 | 118.57 |
| Qatar | 16.90 | 51.89 |
| Republic of Korea | 5.45 | 30.80 |
| Republic of Moldova | 6.13 | 22.54 |
| Romania | 3.66 | 17.28 |
| Russian Federation | 2.77 | 12.91 |
| Rwanda | 28.84 | 56.13 |
| Saint Kitts and Nevis | 63.44 | 118.53 |
| Saint Lucia | 91.98 | 194.35 |
| Saint Vincent and the Grenadines | 109.99 | 215.14 |
| Samoa | 61.70 | 187.11 |
| San Marino | 3.99 | 34.29 |
| Sao Tome and Principe | 34.06 | 37.77 |
| Saudi Arabia | 23.10 | 79.33 |
| Senegal | 76.53 | 74.19 |
| Serbia | 2.25 | 19.61 |
| Seychelles | 28.32 | 75.76 |
| Sierra Leone | 63.71 | 56.07 |
| Singapore | 9.52 | 42.44 |
| Slovakia | 0.91 | 13.45 |
| Slovenia | 1.52 | 15.88 |
| Solomon Islands | 138.12 | 391.28 |
| Somalia | 41.71 | 82.18 |
| South Africa | 39.13 | 88.23 |
| South Sudan | 30.24 | 58.09 |
| Spain | 4.35 | 39.51 |
| Sri Lanka | 36.81 | 90.12 |
| Sudan | 11.97 | 47.97 |
| Suriname | 96.83 | 175.75 |
| Sweden | 5.15 | 29.56 |
| Switzerland | 2.67 | 26.43 |
| Syrian Arab Republic | 26.66 | 64.17 |
| Taiwan (Province of China) | 10.49 | 38.76 |
| Tajikistan | 46.22 | 77.50 |
| Thailand | 24.79 | 57.22 |
| Timor-Leste | 35.10 | 65.18 |
| Togo | 59.89 | 46.51 |
| Tokelau | 65.07 | 203.16 |
| Tonga | 54.84 | 163.24 |
| Trinidad and Tobago | 56.96 | 124.05 |
| Tunisia | 11.05 | 49.39 |
| Turkey | 8.47 | 33.99 |
| Turkmenistan | 36.43 | 74.24 |
| Tuvalu | 73.78 | 223.13 |
| Uganda | 39.03 | 79.39 |
| Ukraine | 6.13 | 26.89 |
| United Arab Emirates | 12.47 | 47.34 |
| United Kingdom | 22.63 | 92.47 |
| United Republic of Tanzania | 32.66 | 61.59 |
| United States of America | 21.66 | 41.40 |
| United States Virgin Islands | 52.74 | 113.07 |
| Uruguay | 5.72 | 13.05 |
| Uzbekistan | 20.91 | 56.48 |
| Vanuatu | 59.32 | 171.32 |
| Venezuela (Bolivarian Republic of) | 65.26 | 124.78 |
| Viet Nam | 24.18 | 47.96 |
| Yemen | 5.90 | 30.18 |
| Zambia | 42.23 | 86.11 |
| Zimbabwe | 159.71 | 140.19 |

**Supplementary table 2. Age distribution of the incidence rate of type 2 diabetes among adolescents in 204 countries.**

| 2019 incidence rate | 15-19 years | 20-24 years |
| --- | --- | --- |
| Afghanistan | 103.98 | 259.60 |
| Albania | 23.90 | 81.10 |
| Algeria | 71.31 | 177.49 |
| American Samoa | 403.39 | 856.41 |
| Andorra | 72.59 | 129.07 |
| Angola | 93.52 | 132.62 |
| Antigua and Barbuda | 134.62 | 204.26 |
| Argentina | 51.47 | 104.99 |
| Armenia | 61.89 | 129.68 |
| Australia | 19.42 | 50.90 |
| Austria | 46.31 | 91.02 |
| Azerbaijan | 70.43 | 138.87 |
| Bahamas | 139.69 | 220.61 |
| Bahrain | 89.31 | 215.36 |
| Bangladesh | 84.45 | 117.44 |
| Barbados | 134.43 | 197.17 |
| Belarus | 14.42 | 79.68 |
| Belgium | 70.91 | 126.90 |
| Belize | 115.84 | 172.91 |
| Benin | 58.28 | 80.95 |
| Bermuda | 86.42 | 127.28 |
| Bhutan | 81.36 | 123.08 |
| Bolivia (Plurinational State of) | 51.26 | 79.31 |
| Bosnia and Herzegovina | 33.65 | 130.76 |
| Botswana | 66.97 | 96.80 |
| Brazil | 67.32 | 106.17 |
| Brunei Darussalam | 111.61 | 214.89 |
| Bulgaria | 34.82 | 122.69 |
| Burkina Faso | 53.33 | 78.63 |
| Burundi | 58.14 | 79.81 |
| Cabo Verde | 62.87 | 92.70 |
| Cambodia | 61.71 | 120.59 |
| Cameroon | 51.39 | 70.64 |
| Canada | 21.37 | 22.66 |
| Central African Republic | 107.62 | 165.29 |
| Chad | 52.58 | 74.57 |
| Chile | 46.35 | 101.37 |
| China | 88.59 | 191.27 |
| Colombia | 126.90 | 174.31 |
| Comoros | 53.56 | 72.28 |
| Congo | 94.36 | 137.16 |
| Cook Islands | 235.46 | 458.32 |
| Costa Rica | 138.28 | 188.79 |
| Croatia | 35.77 | 119.64 |
| Cuba | 135.59 | 201.09 |
| Cyprus | 63.94 | 117.58 |
| Czechia | 49.35 | 170.75 |
| Côte d'Ivoire | 55.85 | 78.75 |
| Democratic People's Republic of Korea | 105.25 | 180.72 |
| Democratic Republic of the Congo | 90.02 | 127.03 |
| Denmark | 68.22 | 118.86 |
| Djibouti | 62.99 | 85.73 |
| Dominica | 170.50 | 284.91 |
| Dominican Republic | 90.21 | 132.31 |
| Ecuador | 63.48 | 95.17 |
| Egypt | 61.23 | 151.08 |
| El Salvador | 131.04 | 188.53 |
| Equatorial Guinea | 93.35 | 134.82 |
| Eritrea | 67.34 | 95.68 |
| Estonia | 22.98 | 108.03 |
| Eswatini | 71.24 | 103.89 |
| Ethiopia | 45.83 | 62.57 |
| Fiji | 255.30 | 481.29 |
| Finland | 126.61 | 216.46 |
| France | 37.72 | 73.20 |
| Gabon | 97.77 | 141.63 |
| Gambia | 47.98 | 68.28 |
| Georgia | 79.20 | 168.47 |
| Germany | 108.27 | 186.56 |
| Ghana | 60.31 | 85.94 |
| Greece | 82.34 | 145.86 |
| Greenland | 26.62 | 26.28 |
| Grenada | 146.95 | 227.73 |
| Guam | 222.91 | 384.88 |
| Guatemala | 150.75 | 213.20 |
| Guinea | 48.89 | 68.09 |
| Guinea-Bissau | 60.99 | 90.27 |
| Guyana | 178.74 | 280.30 |
| Haiti | 159.05 | 246.06 |
| Honduras | 163.87 | 229.60 |
| Hungary | 37.68 | 124.30 |
| Iceland | 81.87 | 139.57 |
| India | 101.12 | 157.38 |
| Indonesia | 50.86 | 96.16 |
| Iran (Islamic Republic of) | 57.96 | 129.26 |
| Iraq | 102.86 | 237.33 |
| Ireland | 59.42 | 109.25 |
| Israel | 67.83 | 125.37 |
| Italy | 60.35 | 108.12 |
| Jamaica | 130.36 | 190.88 |
| Japan | 56.79 | 91.97 |
| Jordan | 81.54 | 190.11 |
| Kazakhstan | 77.58 | 164.42 |
| Kenya | 51.56 | 70.68 |
| Kiribati | 287.99 | 619.33 |
| Kuwait | 114.47 | 282.53 |
| Kyrgyzstan | 54.81 | 110.71 |
| Lao People's Democratic Republic | 75.58 | 145.84 |
| Latvia | 16.31 | 102.33 |
| Lebanon | 76.31 | 185.98 |
| Lesotho | 65.77 | 102.69 |
| Liberia | 66.47 | 96.19 |
| Libya | 81.77 | 218.20 |
| Lithuania | 14.19 | 84.95 |
| Luxembourg | 70.49 | 130.96 |
| Madagascar | 59.50 | 81.64 |
| Malawi | 76.91 | 105.52 |
| Malaysia | 82.25 | 158.83 |
| Maldives | 61.13 | 113.80 |
| Mali | 49.45 | 70.12 |
| Malta | 93.50 | 157.39 |
| Marshall Islands | 407.53 | 908.72 |
| Mauritania | 33.43 | 46.02 |
| Mauritius | 107.13 | 234.11 |
| Mexico | 157.67 | 236.24 |
| Micronesia (Federated States of) | 240.64 | 486.91 |
| Monaco | 77.28 | 135.65 |
| Mongolia | 38.16 | 76.21 |
| Montenegro | 40.49 | 136.79 |
| Morocco | 73.11 | 177.14 |
| Mozambique | 79.30 | 106.80 |
| Myanmar | 69.15 | 136.65 |
| Namibia | 59.63 | 85.34 |
| Nauru | 229.09 | 451.82 |
| Nepal | 102.63 | 145.45 |
| Netherlands | 58.82 | 107.01 |
| New Zealand | 32.71 | 56.70 |
| Nicaragua | 141.26 | 195.96 |
| Niger | 33.01 | 47.53 |
| Nigeria | 31.24 | 44.13 |
| Niue | 362.64 | 709.18 |
| North Macedonia | 33.84 | 123.00 |
| Northern Mariana Islands | 229.94 | 416.71 |
| Norway | 82.30 | 132.61 |
| Oman | 67.32 | 155.62 |
| Pakistan | 110.99 | 162.75 |
| Palau | 267.14 | 546.07 |
| Palestine | 63.72 | 155.41 |
| Panama | 131.71 | 180.29 |
| Papua New Guinea | 253.46 | 506.29 |
| Paraguay | 53.27 | 89.74 |
| Peru | 44.07 | 66.66 |
| Philippines | 52.93 | 89.54 |
| Poland | 40.23 | 129.06 |
| Portugal | 101.10 | 171.37 |
| Puerto Rico | 158.83 | 239.36 |
| Qatar | 79.77 | 214.07 |
| Republic of Korea | 68.93 | 113.63 |
| Republic of Moldova | 21.52 | 115.23 |
| Romania | 24.46 | 97.81 |
| Russian Federation | 23.88 | 67.44 |
| Rwanda | 51.55 | 70.61 |
| Saint Kitts and Nevis | 124.74 | 190.64 |
| Saint Lucia | 179.55 | 288.64 |
| Saint Vincent and the Grenadines | 166.35 | 267.64 |
| Samoa | 229.87 | 442.67 |
| San Marino | 76.60 | 135.08 |
| Sao Tome and Principe | 55.62 | 79.88 |
| Saudi Arabia | 108.82 | 255.36 |
| Senegal | 73.59 | 102.59 |
| Serbia | 32.53 | 118.33 |
| Seychelles | 93.46 | 199.19 |
| Sierra Leone | 33.88 | 46.79 |
| Singapore | 92.92 | 149.44 |
| Slovakia | 25.91 | 100.91 |
| Slovenia | 28.77 | 106.49 |
| Solomon Islands | 247.38 | 530.63 |
| Somalia | 69.85 | 98.20 |
| South Africa | 69.45 | 96.91 |
| South Sudan | 59.04 | 80.28 |
| Spain | 88.16 | 156.05 |
| Sri Lanka | 84.90 | 172.61 |
| Sudan | 73.37 | 180.85 |
| Suriname | 175.57 | 278.38 |
| Sweden | 66.48 | 113.88 |
| Switzerland | 64.94 | 119.19 |
| Syrian Arab Republic | 73.91 | 171.42 |
| Taiwan (Province of China) | 66.69 | 112.04 |
| Tajikistan | 73.33 | 146.48 |
| Thailand | 57.54 | 110.77 |
| Timor-Leste | 58.27 | 109.84 |
| Togo | 34.93 | 49.83 |
| Tokelau | 231.28 | 437.92 |
| Tonga | 199.51 | 378.16 |
| Trinidad and Tobago | 128.70 | 218.58 |
| Tunisia | 77.29 | 194.75 |
| Turkey | 50.21 | 118.82 |
| Turkmenistan | 91.16 | 157.74 |
| Tuvalu | 235.59 | 460.14 |
| Uganda | 76.39 | 105.79 |
| Ukraine | 34.95 | 108.25 |
| United Arab Emirates | 76.34 | 189.28 |
| United Kingdom | 175.41 | 358.43 |
| United Republic of Tanzania | 52.26 | 71.02 |
| United States of America | 72.39 | 60.53 |
| United States Virgin Islands | 137.02 | 209.16 |
| Uruguay | 24.54 | 61.79 |
| Uzbekistan | 68.39 | 133.79 |
| Vanuatu | 213.16 | 419.34 |
| Venezuela (Bolivarian Republic of) | 136.47 | 180.09 |
| Viet Nam | 46.58 | 86.43 |
| Yemen | 49.38 | 117.74 |
| Zambia | 65.86 | 91.32 |
| Zimbabwe | 89.38 | 112.95 |

**Supplementary table3. The Dalys and age-standardized DALY rate of type 2 diabetes among adolescents in 1990 and 2019, and its temporal trends from 1990 to 2019.**

|  |  | DALY No.(95%UI) |  |  | Age-standardized DALY rate (per 100000) No.95%UI) |  |  |
| --- | --- | --- | --- | --- | --- | --- | --- |
| nation | sex | 1990 | 2019 | 1990-2019 EAPC No.(95%CI) | 1990 | 2019 | 1990-2019 EAPC No.(95%CI) |
| Afghanistan | both | 1189.72(714.00,2131.26) | 5216.83(3291.62,7879.40) | 4.88(4.24,5.53) | 52.39(49.43,55.49) | 65.84(64.07,67.66) | 1.15(1.00,1.31) |
| Albania | both | 48.43(26.80,83.45) | 31.99(13.67,56.16) | -1.75(-2.05,-1.46) | 7.66(5.65,10.14) | 7.26(4.96,10.31) | -0.71(-0.96,-0.46) |
| Algeria | both | 1358.87(847.78,2047.05) | 1984.55(1153.30,3108.44) | 1.62(1.13,2.11) | 26.07(24.70,27.49) | 30.55(29.22,31.93) | 0.66(0.55,0.76) |
| American Samoa | both | 11.25(7.76,15.92) | 19.82(13.63,28.58) | 1.58(1.34,1.83) | 114.66(57.75,204.36) | 185.10(112.68,287.02) | 1.58(1.34,1.82) |
| Andorra | both | 0.87(0.27,1.77) | 1.41(0.57,2.73) | 0.86(0.39,1.32) | 9.66(0.15,64.99) | 16.04(1.01,73.58) | 1.69(1.52,1.86) |
| Angola | both | 1348.46(875.68,1984.62) | 3708.52(2480.64,5241.38) | 3.63(3.49,3.77) | 70.46(66.74,74.33) | 67.33(65.18,69.54) | -0.08(-0.21,0.04) |
| Antigua and Barbuda | both | 9.95(7.44,12.95) | 13.94(9.85,19.27) | 0.77(0.54,1.00) | 86.05(41.16,159.13) | 97.65(53.26,164.87) | -0.11(-0.30,0.09) |
| Argentina | both | 632.46(392.11,790.04) | 1200.32(765.28,1746.19) | 2.23(1.96,2.51) | 11.75(10.86,12.71) | 16.45(15.53,17.40) | 1.25(0.91,1.59) |
| Armenia | both | 133.04(81.70,198.83) | 111.01(70.91,165.45) | -0.24(-0.92,0.44) | 23.37(19.57,27.71) | 29.80(24.50,35.96) | 0.57(0.31,0.82) |
| Australia | both | 114.62(61.73,190.22) | 155.48(45.69,354.66) | 0.98(0.14,1.81) | 4.20(3.47,5.04) | 4.88(4.14,5.72) | 0.33(-0.34,0.99) |
| Austria | both | 76.61(34.43,135.62) | 100.47(35.30,196.96) | 1.14(0.78,1.50) | 5.80(4.57,7.29) | 9.28(7.55,11.31) | 1.31(0.82,1.79) |
| Azerbaijan | both | 472.24(313.86,676.54) | 652.90(446.33,929.76) | 0.76(0.23,1.29) | 33.33(30.39,36.48) | 42.63(39.40,46.06) | -0.36(-0.86,0.14) |
| Bahamas | both | 39.96(29.66,52.51) | 67.56(48.03,90.89) | 2.12(1.87,2.36) | 73.89(52.77,100.70) | 108.28(83.99,137.48) | 1.43(1.23,1.63) |
| Bahrain | both | 40.63(29.65,53.61) | 123.68(93.23,166.44) | 5.48(4.66,6.32) | 47.70(34.04,65.26) | 75.19(62.52,89.68) | 2.46(2.11,2.80) |
| Bangladesh | both | 15618.76(9721.31,27780.44) | 19474.43(13345.12,27507.08) | 0.61(0.45,0.78) | 72.29(71.16,73.44) | 65.02(64.11,65.94) | -0.48(-0.58,-0.39) |
| Barbados | both | 48.15(36.93,60.85) | 47.70(34.99,61.65) | -0.45(-0.65,-0.25) | 104.61(77.15,138.80) | 122.24(90.04,162.24) | -0.05(-0.26,0.17) |
| Belarus | both | 167.20(100.16,258.76) | 52.61(17.75,103.81) | -4.24(-5.13,-3.35) | 11.42(9.76,13.29) | 5.59(4.18,7.33) | -3.25(-3.73,-2.76) |
| Belgium | both | 162.15(78.38,288.45) | 224.26(94.90,426.20) | 1.25(0.89,1.62) | 11.00(9.37,12.84) | 16.63(14.53,18.97) | 1.24(1.02,1.47) |
| Belize | both | 30.44(22.78,37.98) | 99.73(75.30,129.43) | 3.83(3.37,4.30) | 81.68(55.24,116.52) | 119.01(96.79,144.83) | 0.86(0.41,1.31) |
| Benin | both | 392.14(278.29,534.55) | 1752.26(1158.94,2457.89) | 5.26(4.86,5.66) | 46.46(41.97,51.30) | 69.97(66.73,73.33) | 1.35(0.98,1.72) |
| Bermuda | both | 3.72(2.57,5.22) | 3.09(2.03,4.52) | -0.56(-0.71,-0.42) | 40.90(10.35,112.39) | 49.92(10.62,144.92) | 0.51(0.41,0.60) |
| Bhutan | both | 50.41(31.99,73.51) | 69.87(45.58,103.97) | 1.83(1.63,2.04) | 36.80(27.35,48.48) | 46.39(36.13,58.73) | 0.74(0.67,0.81) |
| Bolivia (Plurinational State of) | both | 469.72(337.81,635.96) | 808.28(564.74,1134.15) | 1.82(1.62,2.02) | 39.38(35.89,43.12) | 37.55(35.00,40.23) | -0.37(-0.44,-0.31) |
| Bosnia and Herzegovina | both | 85.42(53.46,133.86) | 109.01(75.44,149.23) | 1.96(1.12,2.81) | 10.45(8.35,12.93) | 25.91(21.26,31.32) | 3.66(3.16,4.16) |
| Botswana | both | 159.47(113.99,214.47) | 381.48(255.15,543.70) | 2.46(1.76,3.16) | 59.53(50.62,69.59) | 90.49(81.64,100.05) | 0.96(0.52,1.39) |
| Brazil | both | 17640.09(13567.81,21708.22) | 14685.61(10373.10,20386.93) | -0.65(-0.85,-0.45) | 60.52(59.63,61.42) | 42.34(41.66,43.03) | -1.23(-1.61,-0.84) |
| Brunei Darussalam | both | 49.72(36.39,67.29) | 52.53(38.44,70.86) | 0.51(0.24,0.79) | 96.50(71.54,127.56) | 67.45(50.42,88.54) | -1.10(-1.40,-0.80) |
| Bulgaria | both | 265.42(176.02,371.30) | 107.37(66.06,167.75) | -3.97(-4.42,-3.51) | 22.14(19.56,24.98) | 17.17(14.08,20.75) | -1.76(-2.07,-1.46) |
| Burkina Faso | both | 841.31(546.97,1260.85) | 2838.60(1906.64,4132.10) | 4.14(3.95,4.32) | 49.45(46.15,52.93) | 65.18(62.80,67.63) | 0.81(0.65,0.98) |
| Burundi | both | 681.31(462.43,998.56) | 1178.73(796.76,1651.14) | 2.13(1.97,2.29) | 68.88(63.80,74.26) | 52.31(49.36,55.40) | -1.28(-1.40,-1.16) |
| Cabo Verde | both | 17.88(11.94,25.65) | 55.45(38.58,75.21) | 3.85(2.65,5.08) | 25.68(15.18,40.73) | 54.65(41.21,71.13) | 2.03(1.18,2.89) |
| Cambodia | both | 1721.37(1051.89,2683.68) | 1892.74(1371.50,2495.35) | 0.29(0.07,0.51) | 89.84(85.65,94.19) | 62.56(59.77,65.44) | -1.70(-1.87,-1.52) |
| Cameroon | both | 1025.54(706.42,1385.43) | 4957.06(3237.00,6893.87) | 5.64(5.26,6.04) | 52.32(49.16,55.63) | 83.04(80.74,85.39) | 1.67(1.41,1.92) |
| Canada | both | 51.44(28.07,71.63) | 164.24(38.52,564.21) | 2.14(1.03,3.26) | 1.29(0.96,1.69) | 3.76(3.21,4.38) | 1.55(0.38,2.73) |
| Central African Republic | both | 396.68(273.00,553.63) | 899.28(604.17,1257.91) | 2.80(2.55,3.04) | 75.75(68.48,83.59) | 83.86(78.46,89.53) | 0.36(0.25,0.46) |
| Chad | both | 479.89(322.92,679.45) | 2201.18(1512.74,3076.55) | 5.35(4.97,5.73) | 43.85(40.00,47.98) | 69.53(66.64,72.52) | 1.63(1.20,2.07) |
| Chile | both | 105.53(61.84,191.79) | 258.45(123.67,477.16) | 3.10(2.89,3.31) | 4.12(3.37,4.98) | 9.39(8.28,10.62) | 2.48(2.20,2.76) |
| China | both | 68426.52(44634.92,104187.41) | 51508.96(31471.67,80986.75) | 2.08(1.08,3.09) | 25.68(25.49,25.87) | 31.34(31.07,31.62) | 2.90(2.28,3.52) |
| Colombia | both | 4291.88(3221.44,5466.76) | 5555.23(3754.36,8007.59) | 0.50(0.32,0.68) | 64.81(62.88,66.77) | 67.38(65.62,69.18) | -0.32(-0.54,-0.11) |
| Comoros | both | 41.77(16.00,69.60) | 59.86(35.56,90.79) | 0.47(-0.21,1.15) | 46.23(33.25,62.69) | 43.67(33.32,56.25) | -0.88(-1.53,-0.23) |
| Congo | both | 405.24(279.19,564.08) | 637.46(436.28,921.66) | 1.17(0.92,1.43) | 83.22(75.30,91.76) | 68.35(63.14,73.88) | -0.88(-1.04,-0.72) |
| Cook Islands | both | 3.31(2.39,4.49) | 3.31(2.37,4.62) | -0.44(-0.66,-0.23) | 90.86(20.71,255.66) | 123.76(28.27,345.47) | 0.87(0.75,0.99) |
| Costa Rica | both | 211.35(139.16,299.34) | 510.88(332.58,748.94) | 3.17(2.93,3.41) | 36.09(31.39,41.31) | 65.50(59.93,71.47) | 1.90(1.79,2.01) |
| Croatia | both | 71.63(40.89,118.89) | 55.92(25.20,102.37) | -1.57(-2.00,-1.14) | 10.19(7.96,12.85) | 11.06(8.35,14.43) | -0.38(-0.72,-0.04) |
| Cuba | both | 1558.96(1102.39,2182.43) | 1033.32(656.91,1542.11) | -1.42(-2.06,-0.77) | 67.12(63.83,70.54) | 72.76(68.39,77.34) | -0.09(-0.46,0.29) |
| Cyprus | both | 11.16(5.61,19.35) | 21.86(8.14,42.33) | 2.81(2.27,3.36) | 8.61(4.32,15.47) | 13.00(8.12,20.01) | 1.19(0.94,1.45) |
| Czechia | both | 180.65(95.51,296.45) | 172.54(84.91,299.82) | -0.55(-1.11,0.01) | 12.62(10.85,14.61) | 17.84(15.28,20.71) | 1.06(0.87,1.25) |
| Côte d'Ivoire | both | 1212.88(845.90,1633.55) | 3659.92(2428.78,5228.23) | 3.45(2.73,4.18) | 51.73(48.86,54.73) | 73.27(70.92,75.69) | 1.05(0.60,1.51) |
| Democratic People's Republic of Korea | both | 1477.85(917.04,2178.82) | 1724.74(1153.26,2527.74) | 1.01(0.77,1.24) | 38.44(36.51,40.46) | 42.62(40.63,44.69) | 0.29(0.21,0.37) |
| Democratic Republic of the Congo | both | 4499.53(3043.79,6425.98) | 11241.74(7737.04,16074.91) | 3.28(3.19,3.37) | 62.56(60.74,64.42) | 64.89(63.70,66.10) | 0.07(0.00,0.15) |
| Denmark | both | 82.09(33.42,154.69) | 128.74(59.97,233.78) | 1.77(1.25,2.30) | 10.23(8.13,12.72) | 16.58(13.84,19.73) | 1.79(1.65,1.92) |
| Djibouti | both | 42.92(27.69,64.90) | 105.72(64.83,151.25) | 3.69(3.31,4.07) | 40.29(29.14,54.28) | 51.14(41.85,61.88) | 0.80(0.63,0.98) |
| Dominica | both | 17.15(13.25,21.88) | 16.46(12.01,22.17) | 0.35(-0.20,0.90) | 118.40(69.12,189.56) | 149.72(86.35,241.66) | 0.96(0.32,1.60) |
| Dominican Republic | both | 1326.25(1031.73,1646.68) | 2280.04(1656.92,3096.87) | 2.20(1.93,2.47) | 86.02(81.45,90.77) | 115.57(110.88,120.42) | 1.18(0.94,1.43) |
| Ecuador | both | 582.28(437.39,776.91) | 1722.72(1242.04,2327.30) | 3.17(2.61,3.73) | 29.05(26.74,31.52) | 52.64(50.18,55.19) | 1.52(0.99,2.06) |
| Egypt | both | 1973.33(1308.33,2817.62) | 6247.17(3916.28,9216.43) | 4.93(4.67,5.18) | 18.74(17.92,19.58) | 34.69(33.83,35.56) | 2.86(2.52,3.21) |
| El Salvador | both | 505.75(353.43,707.32) | 968.67(673.09,1374.99) | 2.47(2.27,2.67) | 47.48(43.43,51.82) | 78.98(74.07,84.12) | 1.96(1.68,2.25) |
| Equatorial Guinea | both | 57.80(38.79,82.81) | 233.08(151.37,336.49) | 5.10(4.91,5.29) | 76.55(58.06,99.15) | 69.82(61.14,79.41) | -0.40(-0.63,-0.16) |
| Eritrea | both | 309.63(187.24,467.75) | 778.54(515.67,1105.98) | 3.21(2.94,3.49) | 53.14(47.37,59.42) | 55.19(51.38,59.21) | 0.02(-0.12,0.17) |
| Estonia | both | 18.45(10.64,30.98) | 13.86(6.92,24.35) | -0.60(-1.31,0.12) | 8.54(5.10,13.43) | 10.77(5.86,18.21) | 0.82(0.42,1.21) |
| Eswatini | both | 120.84(90.03,157.71) | 345.93(223.18,507.05) | 4.03(2.85,5.23) | 74.22(61.53,88.83) | 147.35(132.23,163.73) | 2.82(1.91,3.73) |
| Ethiopia | both | 7922.25(5218.21,11350.79) | 8244.96(5581.45,11767.51) | -0.45(-0.65,-0.24) | 83.87(82.03,85.74) | 36.27(35.49,37.06) | -3.32(-3.46,-3.17) |
| Fiji | both | 171.18(123.71,229.00) | 313.87(239.41,405.15) | 1.62(1.00,2.25) | 115.93(99.21,134.67) | 206.82(184.57,231.03) | 1.50(1.04,1.95) |
| Finland | both | 77.43(32.42,144.45) | 234.78(119.23,397.34) | 3.79(2.77,4.82) | 11.07(8.74,13.87) | 37.18(32.57,42.27) | 3.70(2.62,4.79) |
| France | both | 369.37(199.52,608.47) | 449.47(129.34,1017.80) | -0.87(-2.13,0.42) | 4.13(3.72,4.57) | 5.70(5.18,6.25) | -0.45(-1.72,0.84) |
| Gabon | both | 152.30(104.88,214.67) | 277.35(191.58,388.58) | 2.02(1.91,2.13) | 81.94(69.43,96.06) | 80.48(71.29,90.53) | -0.20(-0.28,-0.12) |
| Gambia | both | 68.23(42.67,99.42) | 272.15(187.14,378.22) | 4.52(4.09,4.95) | 35.71(27.74,45.29) | 56.57(50.04,63.73) | 1.44(1.07,1.80) |
| Georgia | both | 226.36(139.67,349.55) | 151.19(93.29,230.96) | -0.95(-1.32,-0.58) | 26.70(23.34,30.42) | 35.73(30.25,41.94) | 1.22(1.06,1.37) |
| Germany | both | 2312.19(1278.69,3745.86) | 3111.20(1615.05,5205.28) | 0.94(0.42,1.47) | 19.58(18.77,20.41) | 32.47(31.34,33.64) | 1.27(0.77,1.77) |
| Ghana | both | 1506.28(1095.60,2088.79) | 4544.55(3163.81,6165.09) | 4.26(4.04,4.47) | 52.42(49.81,55.15) | 71.33(69.27,73.44) | 1.27(1.10,1.45) |
| Greece | both | 159.54(81.40,280.96) | 204.33(87.20,376.66) | 0.67(0.45,0.89) | 9.93(8.45,11.60) | 19.59(16.99,22.47) | 2.53(2.39,2.68) |
| Greenland | both | 0.58(0.38,0.86) | 0.58(0.13,1.40) | -0.52(-1.29,0.25) | 5.24(0.01,59.99) | 7.23(0.02,65.89) | -0.82(-1.68,0.06) |
| Grenada | both | 21.27(16.09,26.48) | 24.87(18.67,32.24) | 0.19(-0.15,0.53) | 134.50(83.49,205.44) | 133.63(86.24,198.50) | -0.24(-0.37,-0.10) |
| Guam | both | 17.39(11.76,24.93) | 24.93(16.04,36.78) | 1.40(1.05,1.74) | 60.86(35.59,98.09) | 88.86(57.43,131.77) | 1.26(1.12,1.41) |
| Guatemala | both | 726.15(507.78,1006.81) | 4191.62(3025.83,5500.42) | 6.03(5.38,6.67) | 50.53(46.92,54.36) | 110.92(107.58,114.33) | 2.33(1.81,2.85) |
| Guinea | both | 502.45(353.21,706.12) | 1847.53(1320.25,2483.20) | 4.68(4.42,4.94) | 47.24(43.20,51.57) | 75.91(72.48,79.46) | 1.81(1.60,2.03) |
| Guinea-Bissau | both | 154.60(106.33,220.96) | 360.75(259.75,485.89) | 3.02(2.59,3.46) | 78.51(66.59,92.01) | 92.48(83.18,102.55) | 0.57(0.26,0.89) |
| Guyana | both | 244.39(182.23,307.15) | 293.63(215.35,387.54) | 0.51(0.22,0.81) | 146.91(129.06,166.54) | 185.64(164.96,208.28) | 0.77(0.50,1.03) |
| Haiti | both | 2300.49(1525.98,3726.58) | 3233.51(2253.44,4657.19) | 1.40(1.23,1.58) | 199.12(191.05,207.44) | 139.03(134.28,143.91) | -1.12(-1.29,-0.95) |
| Honduras | both | 625.63(438.95,841.40) | 1739.87(1158.90,2502.81) | 3.51(3.30,3.71) | 72.61(67.00,78.57) | 84.57(80.64,88.64) | 0.42(0.30,0.55) |
| Hungary | both | 211.16(117.13,347.95) | 135.40(66.60,235.89) | -1.73(-2.06,-1.39) | 14.84(12.91,16.99) | 12.87(10.79,15.24) | -0.39(-0.54,-0.24) |
| Iceland | both | 4.18(1.44,7.87) | 9.58(4.29,17.08) | 3.22(2.95,3.49) | 9.64(2.72,24.50) | 19.82(9.31,37.51) | 2.51(2.29,2.72) |
| India | both | 65776.23(42448.21,101105.85) | 138650.53(91539.51,204694.55) | 2.19(1.86,2.53) | 40.99(40.68,41.31) | 52.38(52.10,52.66) | 0.30(0.03,0.57) |
| Indonesia | both | 22868.77(18114.78,29265.37) | 30420.36(24527.60,37452.42) | 0.47(0.12,0.83) | 61.81(61.01,62.62) | 68.33(67.57,69.10) | 0.06(-0.26,0.38) |
| Iran (Islamic Republic of) | both | 1887.11(1118.62,2922.60) | 2975.99(1877.74,4595.99) | 2.54(1.28,3.82) | 16.42(15.69,17.18) | 25.67(24.76,26.61) | 2.01(1.46,2.57) |
| Iraq | both | 3929.80(2808.14,5159.70) | 7052.47(5059.06,9485.65) | 1.48(1.05,1.91) | 118.76(115.06,122.56) | 80.02(78.16,81.91) | -1.68(-2.07,-1.29) |
| Ireland | both | 57.02(22.76,104.60) | 77.27(29.90,150.85) | 1.17(0.62,1.71) | 9.64(7.29,12.50) | 12.90(10.19,16.12) | 1.30(0.75,1.85) |
| Israel | both | 64.22(25.84,130.13) | 267.07(150.65,437.97) | 3.70(3.13,4.27) | 7.81(6.01,9.97) | 19.47(17.20,21.95) | 2.19(1.68,2.71) |
| Italy | both | 799.85(345.76,1610.71) | 833.33(318.30,1677.17) | -1.84(-4.53,0.92) | 8.66(8.07,9.28) | 13.87(12.94,14.85) | -0.35(-2.90,2.26) |
| Jamaica | both | 243.36(166.84,343.01) | 514.45(375.25,705.54) | 2.46(2.22,2.70) | 49.96(43.88,56.65) | 97.53(89.26,106.37) | 1.90(1.61,2.19) |
| Japan | both | 829.68(589.81,1492.27) | 1357.25(548.82,2691.27) | 0.78(0.08,1.49) | 4.34(4.05,4.65) | 10.77(10.21,11.36) | 2.78(2.15,3.42) |
| Jordan | both | 332.85(237.74,453.55) | 807.75(502.65,1232.61) | 2.98(2.79,3.17) | 38.69(34.64,43.09) | 35.63(33.21,38.17) | -0.43(-0.71,-0.16) |
| Kazakhstan | both | 877.12(548.60,1323.41) | 899.53(562.29,1359.96) | 0.98(0.43,1.54) | 31.88(29.81,34.06) | 35.84(33.53,38.27) | 0.64(0.32,0.97) |
| Kenya | both | 1392.30(975.34,1959.53) | 4077.10(2815.52,5606.91) | 3.77(3.44,4.10) | 30.25(28.68,31.89) | 38.45(37.27,39.65) | 0.96(0.73,1.18) |
| Kiribati | both | 24.56(18.34,31.59) | 60.34(43.67,81.11) | 3.51(2.99,4.04) | 166.51(107.25,247.34) | 277.42(211.87,356.90) | 1.48(0.99,1.97) |
| Kuwait | both | 149.60(108.11,205.08) | 296.06(184.17,444.64) | 2.26(1.81,2.70) | 45.14(38.12,53.17) | 51.20(45.49,57.48) | -0.61(-1.13,-0.07) |
| Kyrgyzstan | both | 203.04(136.26,290.61) | 281.22(172.38,429.99) | 1.12(0.87,1.37) | 25.44(22.05,29.19) | 24.82(22.00,27.91) | -0.51(-0.68,-0.33) |
| Lao People's Democratic Republic | both | 894.05(583.47,1353.49) | 1140.56(815.09,1531.67) | 0.73(0.42,1.05) | 117.57(109.96,125.57) | 81.46(76.80,86.34) | -1.55(-1.71,-1.39) |
| Latvia | both | 43.47(27.82,67.89) | 26.66(17.20,38.71) | -0.93(-1.73,-0.13) | 11.67(8.46,15.72) | 15.18(9.97,22.15) | 1.15(0.83,1.47) |
| Lebanon | both | 155.87(97.13,232.69) | 235.45(138.71,370.07) | 2.38(1.98,2.79) | 26.89(22.83,31.47) | 32.97(28.89,37.47) | 0.97(0.85,1.09) |
| Lesotho | both | 166.44(120.19,219.84) | 457.57(314.97,628.70) | 4.45(3.93,4.98) | 46.83(39.96,54.56) | 106.88(97.31,117.14) | 4.00(3.55,4.46) |
| Liberia | both | 182.97(124.19,254.90) | 721.77(500.55,993.23) | 4.47(4.01,4.93) | 56.24(48.20,65.32) | 73.82(68.53,79.42) | 0.80(0.64,0.95) |
| Libya | both | 183.87(109.58,279.99) | 397.15(222.96,644.02) | 3.01(2.42,3.61) | 22.18(19.08,25.64) | 33.27(30.07,36.71) | 1.79(1.54,2.05) |
| Lithuania | both | 30.28(18.26,47.59) | 20.88(8.52,37.33) | -0.46(-1.22,0.30) | 5.35(3.61,7.63) | 6.41(3.95,9.91) | 1.10(0.50,1.71) |
| Luxembourg | both | 6.39(2.73,11.83) | 12.52(5.20,23.29) | 3.01(2.49,3.53) | 11.55(4.35,25.66) | 16.15(8.47,28.32) | 1.58(1.29,1.87) |
| Madagascar | both | 1466.90(1011.07,2072.58) | 2613.19(1748.75,3675.03) | 2.04(1.75,2.33) | 64.70(61.42,68.11) | 48.40(46.56,50.29) | -0.93(-1.20,-0.67) |
| Malawi | both | 1135.16(768.42,1625.51) | 2274.54(1510.29,3248.90) | 1.88(1.58,2.18) | 61.54(58.01,65.23) | 58.68(56.28,61.16) | -0.55(-0.83,-0.27) |
| Malaysia | both | 1491.00(1100.76,1963.51) | 2541.83(1751.35,3655.80) | 1.54(1.24,1.85) | 44.26(42.04,46.56) | 44.36(42.65,46.12) | -0.52(-0.75,-0.28) |
| Maldives | both | 16.94(11.57,24.54) | 24.68(16.48,35.87) | 2.30(2.00,2.59) | 39.38(22.90,63.24) | 33.09(21.23,49.69) | -0.39(-0.79,0.01) |
| Mali | both | 870.28(594.26,1228.16) | 3021.75(2063.46,4280.94) | 4.09(3.91,4.28) | 57.92(54.12,61.91) | 68.13(65.71,70.61) | 0.22(0.01,0.43) |
| Malta | both | 11.83(6.09,19.37) | 13.14(6.73,22.85) | -0.19(-0.53,0.16) | 23.26(11.94,40.88) | 26.66(14.21,46.32) | -0.19(-0.56,0.18) |
| Marshall Islands | both | 10.63(7.37,14.87) | 20.94(14.27,30.09) | 2.10(1.50,2.71) | 129.17(63.39,234.79) | 207.13(128.08,317.14) | 1.62(1.38,1.86) |
| Mauritania | both | 188.88(131.06,272.24) | 378.45(241.25,542.86) | 2.39(2.16,2.61) | 48.32(41.66,55.75) | 46.15(41.60,51.06) | -0.14(-0.36,0.07) |
| Mauritius | both | 75.82(52.45,106.40) | 156.74(115.02,207.98) | 3.81(3.35,4.28) | 35.77(28.16,44.83) | 82.61(70.18,96.64) | 4.21(3.65,4.78) |
| Mexico | both | 17320.34(12808.90,22066.43) | 20555.31(14515.89,28346.99) | 0.62(0.39,0.85) | 96.23(94.80,97.68) | 94.37(93.08,95.67) | 0.05(-0.19,0.30) |
| Micronesia (Federated States of) | both | 17.43(12.56,23.74) | 38.94(27.31,55.17) | 2.48(1.84,3.11) | 93.09(54.39,149.35) | 192.85(137.05,263.91) | 2.45(1.95,2.96) |
| Monaco | both | 0.36(0.12,0.71) | 0.67(0.29,1.22) | 2.75(2.21,3.29) | 10.18(0.00,160.98) | 18.52(0.10,139.40) | 2.19(1.99,2.39) |
| Mongolia | both | 103.68(67.06,151.62) | 117.95(71.95,180.72) | 0.66(0.19,1.13) | 23.74(19.39,28.79) | 24.80(20.52,29.75) | 0.02(-0.21,0.26) |
| Montenegro | both | 17.52(10.27,27.46) | 13.56(7.82,22.47) | -0.85(-1.17,-0.52) | 16.66(9.79,26.52) | 16.19(8.75,27.50) | -0.04(-0.25,0.17) |
| Morocco | both | 1022.75(614.15,1570.75) | 1883.90(1103.93,2990.51) | 2.35(2.01,2.69) | 20.30(19.07,21.58) | 30.80(29.43,32.22) | 1.62(1.46,1.78) |
| Mozambique | both | 1115.63(742.75,1571.00) | 4112.86(2818.78,5868.60) | 4.60(4.33,4.87) | 49.44(46.56,52.45) | 70.84(68.69,73.05) | 1.57(1.34,1.81) |
| Myanmar | both | 7812.74(5084.29,11957.08) | 8169.27(5903.94,11060.54) | -0.24(-0.41,-0.07) | 96.69(94.56,98.86) | 85.02(83.18,86.88) | -0.82(-0.97,-0.66) |
| Namibia | both | 137.51(90.87,186.50) | 262.25(176.00,358.27) | 2.00(1.42,2.59) | 46.85(39.33,55.40) | 55.80(49.25,62.97) | 0.35(-0.08,0.78) |
| Nauru | both | 2.06(1.44,2.92) | 4.01(2.89,5.90) | 2.04(1.65,2.44) | 111.40(14.12,398.01) | 192.79(52.64,495.02) | 1.73(1.43,2.03) |
| Nepal | both | 1572.42(947.10,2583.37) | 3947.36(2628.96,5706.77) | 3.11(2.95,3.27) | 43.72(41.59,45.94) | 62.20(60.27,64.17) | 1.11(0.90,1.32) |
| Netherlands | both | 178.30(69.76,351.73) | 263.89(95.02,512.93) | 2.52(1.61,3.44) | 7.04(6.04,8.17) | 12.26(10.82,13.83) | 2.89(2.29,3.49) |
| New Zealand | both | 20.58(12.00,44.71) | 69.42(25.02,139.90) | 4.07(3.83,4.32) | 3.58(2.20,5.49) | 11.77(9.17,14.90) | 3.67(3.37,3.97) |
| Nicaragua | both | 427.18(304.08,597.39) | 1031.24(688.73,1478.71) | 3.21(3.09,3.33) | 56.49(51.24,62.13) | 82.57(77.60,87.77) | 1.44(1.24,1.64) |
| Niger | both | 603.56(376.55,943.78) | 2362.63(1544.70,3500.72) | 4.26(3.95,4.56) | 41.51(38.25,44.97) | 51.87(49.79,54.02) | 0.40(0.16,0.64) |
| Nigeria | both | 6150.19(4181.34,8856.12) | 14709.82(9922.25,21447.33) | 2.68(2.42,2.94) | 35.10(34.23,35.99) | 33.39(32.85,33.93) | -0.43(-0.65,-0.20) |
| Niue | both | 0.40(0.28,0.54) | 0.47(0.33,0.65) | 0.30(0.09,0.50) | 106.15(0.02,1233.71) | 194.22(0.13,1944.04) | 2.22(2.00,2.43) |
| North Macedonia | both | 38.45(20.23,65.95) | 36.65(19.89,62.41) | 0.07(-0.37,0.51) | 11.16(7.91,15.30) | 12.68(8.91,17.62) | 0.39(0.13,0.64) |
| Northern Mariana Islands | both | 6.02(3.98,8.61) | 7.15(4.62,10.46) | 0.28(-0.54,1.12) | 63.23(23.07,142.04) | 97.01(39.36,199.71) | 1.54(1.36,1.71) |
| Norway | both | 131.08(61.51,235.66) | 172.47(79.85,306.51) | -0.74(-1.24,-0.25) | 19.48(16.28,23.14) | 25.18(21.56,29.25) | -1.05(-1.63,-0.47) |
| Oman | both | 64.83(39.44,98.84) | 226.62(139.70,352.41) | 4.56(4.19,4.94) | 20.51(15.82,26.15) | 30.06(26.17,34.44) | 1.28(0.99,1.58) |
| Pakistan | both | 10564.45(7140.68,15361.64) | 46715.99(33435.05,63746.59) | 5.17(5.09,5.25) | 50.36(49.40,51.33) | 103.27(102.33,104.21) | 2.49(2.43,2.56) |
| Palau | both | 2.76(1.93,3.78) | 3.80(2.66,5.30) | 1.05(0.54,1.57) | 88.42(16.62,269.12) | 161.86(42.15,426.28) | 2.07(1.86,2.28) |
| Palestine | both | 141.26(95.70,203.68) | 375.17(250.80,535.58) | 3.57(3.33,3.82) | 34.68(29.19,40.92) | 36.75(33.13,40.67) | 0.01(-0.33,0.35) |
| Panama | both | 193.92(132.95,274.03) | 510.96(346.77,711.14) | 3.20(3.11,3.28) | 39.90(34.49,45.94) | 71.85(65.75,78.36) | 1.97(1.84,2.09) |
| Papua New Guinea | both | 986.73(666.36,1400.11) | 3418.36(2401.60,4680.56) | 4.37(4.23,4.51) | 122.34(114.82,130.22) | 180.92(174.91,187.09) | 1.19(1.04,1.34) |
| Paraguay | both | 191.09(128.04,279.06) | 519.79(358.67,730.22) | 3.61(3.18,4.04) | 25.93(22.38,29.89) | 40.09(36.71,43.69) | 1.40(1.15,1.65) |
| Peru | both | 878.03(620.88,1166.23) | 1607.65(1072.52,2333.75) | 2.27(2.10,2.43) | 20.02(18.71,21.39) | 27.73(26.39,29.13) | 1.30(1.10,1.50) |
| Philippines | both | 18038.32(14429.71,23103.61) | 15130.56(12003.43,20232.23) | -1.33(-1.75,-0.91) | 141.73(139.67,143.82) | 72.27(71.12,73.43) | -3.06(-3.48,-2.64) |
| Poland | both | 619.75(387.08,954.71) | 553.64(278.13,948.39) | 0.22(-0.26,0.70) | 12.07(11.14,13.06) | 13.23(12.15,14.39) | 0.94(0.16,1.72) |
| Portugal | both | 392.69(229.36,607.90) | 338.74(173.27,569.74) | -1.41(-1.75,-1.08) | 23.93(21.63,26.42) | 29.18(26.16,32.47) | 0.24(0.06,0.43) |
| Puerto Rico | both | 302.38(198.91,446.83) | 386.63(249.71,578.91) | 0.72(0.45,1.00) | 48.57(43.24,54.37) | 82.97(74.90,91.68) | 1.69(1.54,1.84) |
| Qatar | both | 23.92(16.25,35.12) | 165.11(94.56,264.09) | 9.05(8.13,9.97) | 35.30(22.47,53.27) | 33.91(28.43,40.41) | 0.28(-0.27,0.83) |
| Republic of Korea | both | 1244.76(947.96,1669.53) | 1188.00(653.81,1950.98) | -0.27(-0.59,0.06) | 13.63(12.88,14.40) | 17.78(16.77,18.83) | 0.84(0.39,1.29) |
| Republic of Moldova | both | 140.19(92.70,211.99) | 59.18(34.60,93.00) | -2.72(-3.31,-2.13) | 22.15(18.63,26.14) | 14.11(10.74,18.23) | -1.58(-1.77,-1.38) |
| Romania | both | 500.65(341.19,769.24) | 207.72(112.41,341.49) | -3.09(-3.41,-2.76) | 12.78(11.69,13.95) | 10.28(8.93,11.78) | -0.49(-0.65,-0.33) |
| Russian Federation | both | 2196.98(1143.28,3720.51) | 1120.71(512.43,2050.09) | -2.69(-3.53,-1.85) | 10.75(10.31,11.21) | 7.70(7.25,8.16) | -1.71(-2.00,-1.41) |
| Rwanda | both | 905.73(620.74,1250.67) | 1089.39(702.23,1587.60) | 0.10(-0.39,0.59) | 68.15(63.78,72.75) | 42.10(39.64,44.68) | -2.74(-3.15,-2.33) |
| Saint Kitts and Nevis | both | 6.10(4.50,8.11) | 8.08(5.60,11.27) | 1.25(1.03,1.48) | 76.38(28.30,165.82) | 90.22(39.12,177.97) | 0.58(0.35,0.82) |
| Saint Lucia | both | 38.43(30.05,49.08) | 39.62(29.09,52.63) | 0.30(0.17,0.43) | 134.99(95.71,185.11) | 141.74(101.05,193.67) | 0.24(0.08,0.40) |
| Saint Vincent and the Grenadines | both | 33.16(25.99,41.08) | 29.38(22.26,37.34) | -0.91(-1.08,-0.73) | 143.90(99.11,202.23) | 161.10(108.20,231.02) | -0.01(-0.17,0.14) |
| Samoa | both | 25.85(17.54,35.54) | 49.05(34.43,68.48) | 1.92(1.79,2.04) | 74.75(48.68,110.02) | 122.66(90.64,162.48) | 1.61(1.37,1.85) |
| San Marino | both | 0.42(0.14,0.87) | 0.80(0.36,1.48) | 2.24(1.91,2.57) | 10.34(0.00,118.94) | 18.72(0.21,126.82) | 2.19(2.02,2.36) |
| Sao Tome and Principe | both | 4.75(2.66,7.38) | 14.92(9.95,21.59) | 3.39(2.96,3.82) | 20.37(6.33,49.50) | 35.86(20.00,59.56) | 1.54(1.21,1.87) |
| Saudi Arabia | both | 1315.37(924.51,1769.05) | 3194.36(2033.06,4782.26) | 3.78(3.44,4.12) | 41.59(39.37,43.90) | 50.43(48.69,52.23) | 1.00(0.74,1.27) |
| Senegal | both | 832.12(575.86,1141.39) | 2334.58(1598.18,3182.23) | 3.62(3.26,3.98) | 57.17(53.34,61.21) | 75.39(72.36,78.52) | 1.08(0.80,1.36) |
| Serbia | both | 169.48(101.14,268.54) | 115.98(54.55,205.62) | -1.87(-2.11,-1.63) | 12.77(10.92,14.85) | 10.69(8.83,12.83) | -0.79(-0.87,-0.71) |
| Seychelles | both | 3.51(2.30,5.18) | 7.51(5.09,10.72) | 2.82(2.68,2.96) | 24.18(5.86,65.61) | 51.38(21.45,104.40) | 2.65(2.53,2.76) |
| Sierra Leone | both | 214.62(133.04,316.89) | 1048.79(682.04,1476.81) | 6.48(5.95,7.01) | 32.50(28.29,37.15) | 59.99(56.41,63.74) | 2.32(1.91,2.74) |
| Singapore | both | 54.63(27.29,94.49) | 142.74(68.35,249.39) | 4.36(3.89,4.83) | 8.40(6.31,10.99) | 25.52(21.50,30.11) | 3.98(3.72,4.23) |
| Slovakia | both | 57.76(32.86,95.72) | 42.47(15.46,82.54) | -1.12(-1.65,-0.58) | 7.47(5.67,9.67) | 7.01(5.06,9.50) | -0.26(-0.37,-0.16) |
| Slovenia | both | 24.05(12.85,41.77) | 17.04(7.14,31.87) | -1.22(-1.67,-0.77) | 7.94(5.09,11.85) | 8.50(4.95,13.70) | 0.18(0.00,0.36) |
| Solomon Islands | both | 83.66(57.33,121.77) | 320.48(227.26,442.47) | 4.47(4.17,4.76) | 124.14(98.90,153.95) | 261.18(233.34,291.45) | 2.62(2.42,2.81) |
| Somalia | both | 626.56(414.80,916.23) | 2508.60(1717.51,3498.64) | 4.02(3.53,4.51) | 53.61(49.39,58.10) | 61.38(59.00,63.84) | 0.40(0.28,0.51) |
| South Africa | both | 4404.76(3569.78,5466.90) | 5884.83(4305.31,7700.93) | 1.62(0.77,2.48) | 57.65(55.96,59.38) | 63.00(61.40,64.63) | 0.70(0.14,1.27) |
| South Sudan | both | 481.47(306.62,749.31) | 803.32(511.64,1187.99) | 1.46(1.17,1.76) | 39.34(35.90,43.02) | 43.78(40.77,46.95) | 0.19(0.06,0.32) |
| Spain | both | 650.99(349.57,1116.11) | 967.20(430.34,1782.99) | 1.36(1.02,1.69) | 9.86(9.12,10.65) | 21.44(20.11,22.83) | 2.90(2.44,3.37) |
| Sri Lanka | both | 1212.26(855.63,1650.15) | 2087.36(1497.68,2886.88) | 1.70(1.43,1.96) | 36.57(34.54,38.68) | 62.72(60.06,65.47) | 1.86(1.68,2.04) |
| Sudan | both | 817.35(476.88,1306.84) | 2439.05(1449.43,3825.99) | 4.13(3.98,4.27) | 21.30(19.86,22.82) | 29.47(28.31,30.66) | 1.51(1.34,1.67) |
| Suriname | both | 77.30(52.86,103.32) | 124.78(89.99,168.64) | 1.81(1.49,2.13) | 95.87(75.69,119.85) | 135.19(112.51,161.11) | 1.00(0.65,1.35) |
| Sweden | both | 150.33(48.64,315.83) | 195.62(77.59,389.64) | 1.25(0.46,2.04) | 12.57(10.64,14.76) | 17.02(14.72,19.58) | 0.76(0.09,1.44) |
| Switzerland | both | 96.75(31.29,193.75) | 143.46(56.76,271.84) | 1.41(0.77,2.06) | 9.34(7.56,11.45) | 14.22(11.98,16.77) | 0.98(0.43,1.55) |
| Syrian Arab Republic | both | 1585.72(1165.89,2056.63) | 1363.26(926.46,1913.80) | -1.45(-2.13,-0.77) | 62.44(59.39,65.60) | 44.90(42.53,47.36) | -1.45(-1.78,-1.13) |
| Taiwan (Province of China) | both | 1021.23(684.45,1492.25) | 720.20(436.60,1070.99) | -1.68(-2.02,-1.33) | 26.86(25.23,28.56) | 24.23(22.48,26.09) | -0.79(-1.04,-0.53) |
| Tajikistan | both | 427.13(315.24,569.42) | 1084.07(756.61,1446.19) | 3.01(2.64,3.38) | 41.59(37.74,45.74) | 61.42(57.82,65.20) | 0.61(0.12,1.10) |
| Thailand | both | 3837.63(2739.27,5072.87) | 3861.40(2742.72,5369.78) | -1.22(-1.71,-0.73) | 32.02(31.01,33.05) | 40.56(39.28,41.86) | -0.43(-0.91,0.06) |
| Timor-Leste | both | 74.92(40.44,134.75) | 142.05(90.48,196.89) | 1.74(1.03,2.46) | 50.29(39.55,63.08) | 49.72(41.86,58.64) | -0.62(-1.22,-0.03) |
| Togo | both | 299.13(205.90,416.22) | 811.13(545.26,1125.85) | 3.36(3.04,3.69) | 41.50(36.92,46.50) | 53.39(49.77,57.20) | 0.80(0.55,1.06) |
| Tokelau | both | 0.21(0.15,0.29) | 0.30(0.21,0.41) | 0.68(0.38,0.99) | 78.48(0.00,1617.93) | 132.20(0.00,1936.75) | 1.71(1.55,1.87) |
| Tonga | both | 12.90(8.93,17.83) | 19.99(14.17,27.60) | 1.58(1.44,1.72) | 66.19(35.04,114.12) | 107.53(65.61,166.49) | 1.83(1.73,1.93) |
| Trinidad and Tobago | both | 154.01(110.22,212.35) | 160.29(113.47,228.29) | 0.21(-0.43,0.86) | 71.49(60.65,83.73) | 89.57(76.24,104.58) | 0.77(0.59,0.94) |
| Tunisia | both | 294.85(171.40,470.68) | 487.83(273.45,784.78) | 2.00(1.58,2.43) | 17.57(15.62,19.69) | 29.69(27.11,32.45) | 1.94(1.88,2.01) |
| Turkey | both | 3668.40(2535.52,5111.86) | 2850.72(1685.17,4575.55) | -1.00(-1.53,-0.46) | 31.15(30.15,32.18) | 20.88(20.12,21.66) | -1.34(-1.84,-0.84) |
| Turkmenistan | both | 217.85(147.59,303.74) | 450.28(295.58,633.09) | 2.94(2.47,3.40) | 30.48(26.57,34.82) | 54.81(49.86,60.12) | 2.15(1.90,2.40) |
| Tuvalu | both | 1.56(1.10,2.20) | 3.24(2.31,4.47) | 2.95(2.81,3.09) | 97.83(7.61,413.18) | 146.38(32.69,413.30) | 1.45(1.28,1.61) |
| Uganda | both | 1474.88(989.96,2096.65) | 4973.67(3352.30,7302.26) | 3.63(3.21,4.05) | 43.69(41.49,45.99) | 58.65(57.02,60.31) | 0.37(-0.01,0.75) |
| Ukraine | both | 1205.94(674.99,1869.80) | 722.50(416.40,1141.87) | -2.31(-2.88,-1.74) | 17.04(16.09,18.03) | 16.22(15.06,17.46) | -1.03(-1.50,-0.55) |
| United Arab Emirates | both | 67.76(41.18,103.83) | 193.97(109.24,311.98) | 4.70(2.70,6.74) | 23.33(18.04,29.79) | 29.42(25.43,33.87) | 0.95(0.73,1.16) |
| United Kingdom | both | 1751.62(900.64,2919.11) | 4640.10(2584.61,7548.09) | 6.33(5.11,7.57) | 20.18(19.25,21.16) | 56.58(54.96,58.23) | 5.96(4.76,7.18) |
| United Republic of Tanzania | both | 2049.85(1357.07,2968.31) | 5144.81(3449.54,7431.81) | 2.84(2.66,3.03) | 40.82(39.06,42.63) | 46.73(45.45,48.02) | 0.27(0.13,0.41) |
| United States of America | both | 3475.54(1862.72,6727.36) | 13631.00(7084.25,22376.96) | 5.45(5.04,5.86) | 8.94(8.64,9.24) | 31.26(30.73,31.79) | 4.73(4.38,5.07) |
| United States Virgin Islands | both | 10.63(7.74,14.13) | 9.95(6.70,14.30) | -0.83(-1.18,-0.48) | 62.65(30.74,114.09) | 82.07(39.26,151.42) | 0.22(-0.16,0.59) |
| Uruguay | both | 48.83(34.81,65.46) | 47.77(33.06,68.34) | -0.35(-0.75,0.05) | 9.98(7.38,13.20) | 9.28(6.84,12.33) | -0.43(-0.86,-0.01) |
| Uzbekistan | both | 898.23(531.45,1339.49) | 2275.16(1512.47,3353.72) | 3.09(2.47,3.71) | 22.66(21.20,24.19) | 38.20(36.64,39.80) | 1.26(0.79,1.73) |
| Vanuatu | both | 17.15(11.34,24.80) | 62.30(42.90,87.09) | 4.74(4.57,4.91) | 61.34(35.81,98.19) | 113.76(87.28,145.77) | 2.22(2.13,2.30) |
| Venezuela (Bolivarian Republic of) | both | 2020.51(1478.19,2671.34) | 4022.49(2859.00,5536.55) | 2.81(2.38,3.24) | 53.95(51.62,56.35) | 94.19(91.30,97.15) | 1.87(1.40,2.34) |
| Viet Nam | both | 3812.36(2579.03,5441.56) | 5116.07(3624.71,7055.69) | 1.57(1.32,1.83) | 28.40(27.51,29.32) | 35.74(34.77,36.74) | 0.94(0.63,1.25) |
| Yemen | both | 284.68(138.86,486.74) | 1018.15(529.34,1738.57) | 4.15(3.65,4.66) | 13.19(11.69,14.82) | 17.70(16.63,18.83) | 0.73(0.10,1.35) |
| Zambia | both | 1000.99(701.48,1345.67) | 2398.11(1679.20,3359.11) | 2.76(2.60,2.92) | 60.45(56.75,64.34) | 63.56(61.04,66.16) | -0.16(-0.45,0.13) |
| Zimbabwe | both | 1736.82(1334.32,2217.19) | 4604.07(3310.23,6288.63) | 3.24(2.72,3.76) | 80.15(76.41,84.04) | 150.22(145.91,154.63) | 2.41(1.99,2.83) |
| Afghanistan | female | 968.88(558.78,1902.01) | 4105.48(2460.69,6459.80) | 4.90(4.28,5.53) | 84.04(78.80,89.55) | 106.10(102.87,109.39) | 1.11(0.94,1.28) |
| Albania | female | 26.56(14.80,45.41) | 16.08(7.00,28.62) | -1.99(-2.25,-1.74) | 8.53(5.60,12.45) | 7.62(4.36,12.50) | -0.74(-0.94,-0.55) |
| Algeria | female | 833.72(519.27,1307.32) | 1166.40(711.80,1792.80) | 1.54(1.07,2.02) | 32.15(30.01,34.41) | 36.64(34.57,38.81) | 0.63(0.53,0.74) |
| American Samoa | female | 5.32(3.67,7.48) | 9.97(6.87,14.25) | 1.90(1.71,2.09) | 108.28(36.68,249.44) | 187.05(89.47,345.51) | 1.79(1.62,1.96) |
| Andorra | female | 0.48(0.16,0.92) | 0.85(0.38,1.60) | 1.04(0.62,1.46) | 11.33(0.01,120.97) | 20.35(0.29,130.68) | 1.96(1.79,2.12) |
| Angola | female | 562.97(356.89,894.53) | 1597.61(1046.15,2277.99) | 3.77(3.58,3.95) | 59.13(54.34,64.24) | 55.88(53.17,58.69) | -0.13(-0.24,-0.01) |
| Antigua and Barbuda | female | 6.79(4.98,8.87) | 8.08(5.66,11.18) | 0.27(0.10,0.44) | 116.35(45.95,244.09) | 115.66(50.11,229.07) | -0.47(-0.58,-0.36) |
| Argentina | female | 529.53(292.78,687.42) | 811.06(533.46,1179.97) | 1.53(1.20,1.86) | 19.55(17.92,21.29) | 22.33(20.82,23.92) | 0.58(0.20,0.97) |
| Armenia | female | 79.23(50.33,119.32) | 60.82(37.55,92.20) | -0.26(-1.02,0.51) | 27.91(22.10,34.80) | 33.95(25.92,43.81) | 0.60(0.35,0.84) |
| Australia | female | 63.74(35.54,104.68) | 97.94(27.90,217.04) | 1.76(0.74,2.80) | 4.73(3.64,6.04) | 6.25(5.07,7.63) | 1.13(0.28,1.99) |
| Austria | female | 47.91(23.32,83.68) | 65.09(24.58,117.49) | 1.30(0.99,1.60) | 7.44(5.47,9.94) | 12.51(9.65,16.01) | 1.51(1.20,1.81) |
| Azerbaijan | female | 298.73(199.25,424.63) | 401.72(268.78,572.05) | 1.11(0.63,1.58) | 41.86(37.24,46.91) | 54.54(49.30,60.21) | 0.14(-0.19,0.47) |
| Bahamas | female | 26.42(18.82,35.23) | 41.57(29.23,55.81) | 1.77(1.48,2.06) | 97.35(63.83,142.41) | 132.84(95.55,180.05) | 1.09(0.84,1.33) |
| Bahrain | female | 25.39(17.34,34.60) | 64.44(48.40,86.81) | 5.02(4.23,5.82) | 63.21(40.97,93.56) | 81.62(62.91,104.17) | 2.03(1.52,2.54) |
| Bangladesh | female | 10617.61(6046.88,22160.24) | 13278.82(8927.75,19549.65) | 0.68(0.59,0.77) | 95.43(93.62,97.27) | 84.55(83.11,86.00) | -0.40(-0.49,-0.31) |
| Barbados | female | 34.10(25.54,43.10) | 31.87(23.37,41.76) | -0.59(-0.85,-0.34) | 149.52(103.57,209.20) | 164.77(112.60,232.90) | -0.22(-0.45,0.00) |
| Belarus | female | 107.29(65.05,166.76) | 29.08(11.56,56.81) | -4.89(-5.80,-3.97) | 14.57(11.95,17.61) | 6.37(4.27,9.18) | -3.76(-4.31,-3.21) |
| Belgium | female | 102.85(51.59,180.30) | 142.57(64.27,260.08) | 1.21(0.87,1.56) | 14.28(11.65,17.35) | 21.52(18.13,25.38) | 1.16(0.94,1.38) |
| Belize | female | 22.69(16.44,28.76) | 65.30(48.09,83.12) | 3.57(3.14,4.00) | 121.43(76.65,183.11) | 153.56(118.58,195.69) | 0.60(0.21,0.98) |
| Benin | female | 222.89(146.52,327.67) | 1102.24(689.10,1614.56) | 5.68(5.28,6.08) | 49.66(43.35,56.62) | 85.73(80.74,90.95) | 1.92(1.56,2.28) |
| Bermuda | female | 2.32(1.59,3.24) | 1.86(1.23,2.72) | -0.71(-0.85,-0.57) | 51.60(7.63,181.70) | 59.50(6.42,227.45) | 0.24(0.09,0.39) |
| Bhutan | female | 28.59(17.91,43.21) | 35.25(22.11,53.44) | 1.17(1.01,1.34) | 45.94(30.66,66.23) | 49.39(34.44,68.75) | 0.04(-0.04,0.13) |
| Bolivia (Plurinational State of) | female | 326.33(220.20,464.93) | 473.35(323.03,673.38) | 1.16(0.96,1.37) | 53.67(48.00,59.84) | 44.40(40.49,48.60) | -0.89(-0.96,-0.81) |
| Bosnia and Herzegovina | female | 44.78(28.76,66.75) | 54.91(37.31,78.79) | 1.61(0.62,2.60) | 11.50(8.38,15.41) | 26.56(19.99,34.70) | 3.29(2.61,3.98) |
| Botswana | female | 70.98(47.26,99.79) | 183.09(120.60,268.38) | 3.39(2.94,3.85) | 49.98(39.01,63.14) | 86.66(74.56,100.18) | 2.08(1.87,2.30) |
| Brazil | female | 11170.21(8134.98,13468.68) | 7667.07(5406.45,11005.91) | -1.50(-1.71,-1.29) | 75.80(74.40,77.22) | 44.66(43.67,45.67) | -2.00(-2.36,-1.63) |
| Brunei Darussalam | female | 30.49(21.77,41.67) | 34.21(25.43,45.13) | 0.95(0.64,1.26) | 123.05(83.28,175.62) | 94.62(65.57,132.52) | -0.50(-0.82,-0.19) |
| Bulgaria | female | 178.46(106.36,248.74) | 68.11(42.01,103.80) | -4.29(-4.78,-3.79) | 30.35(26.06,35.14) | 22.51(17.48,28.54) | -2.02(-2.37,-1.68) |
| Burkina Faso | female | 451.33(275.63,726.41) | 1485.77(962.88,2234.41) | 4.05(3.83,4.28) | 51.53(46.88,56.53) | 67.28(63.90,70.80) | 0.75(0.56,0.95) |
| Burundi | female | 343.75(219.64,557.93) | 611.89(396.57,898.89) | 2.24(2.08,2.40) | 66.97(60.08,74.45) | 51.44(47.44,55.70) | -1.22(-1.34,-1.10) |
| Cabo Verde | female | 11.35(7.65,17.00) | 33.40(22.06,45.07) | 3.75(2.37,5.15) | 32.30(16.32,57.38) | 66.62(45.96,93.49) | 1.99(0.93,3.07) |
| Cambodia | female | 929.20(535.02,1802.39) | 890.07(601.49,1208.18) | -0.37(-0.68,-0.06) | 93.62(87.70,99.84) | 60.01(56.13,64.10) | -2.13(-2.34,-1.91) |
| Cameroon | female | 592.61(381.64,832.20) | 3007.98(1852.39,4417.75) | 5.99(5.57,6.41) | 58.41(53.80,63.31) | 100.64(97.07,104.31) | 2.08(1.81,2.35) |
| Canada | female | 29.21(13.58,46.98) | 106.38(21.48,321.11) | 2.55(1.38,3.74) | 1.48(0.99,2.13) | 4.98(4.08,6.03) | 2.00(0.76,3.25) |
| Central African Republic | female | 157.13(106.63,231.41) | 391.40(258.83,574.90) | 3.32(3.05,3.59) | 58.15(49.41,67.99) | 70.40(63.60,77.74) | 0.77(0.62,0.91) |
| Chad | female | 274.97(171.53,433.59) | 1343.64(843.80,1981.87) | 5.65(5.26,6.05) | 49.18(43.52,55.38) | 84.07(79.61,88.71) | 1.97(1.55,2.39) |
| Chile | female | 79.21(41.97,143.68) | 171.75(88.20,291.81) | 2.81(2.61,3.02) | 6.14(4.86,7.65) | 12.69(10.86,14.75) | 2.25(2.01,2.49) |
| China | female | 31676.11(21194.05,48002.16) | 20146.41(12762.08,31800.80) | 1.38(0.43,2.34) | 24.37(24.11,24.64) | 25.81(25.46,26.17) | 2.22(1.66,2.78) |
| Colombia | female | 2541.88(1830.33,3227.72) | 3036.89(2058.13,4413.91) | 0.28(0.07,0.49) | 74.49(71.62,77.44) | 74.74(72.11,77.46) | -0.36(-0.59,-0.12) |
| Comoros | female | 22.89(7.33,44.20) | 31.64(16.97,49.72) | 0.22(-0.60,1.05) | 49.84(31.51,75.12) | 46.33(31.61,65.55) | -1.06(-1.86,-0.26) |
| Congo | female | 165.27(107.26,231.20) | 323.26(221.28,466.48) | 2.08(1.63,2.53) | 66.59(56.81,77.60) | 67.65(60.47,75.46) | -0.06(-0.35,0.24) |
| Cook Islands | female | 1.87(1.32,2.52) | 1.79(1.27,2.52) | -0.56(-0.73,-0.39) | 104.29(11.27,396.23) | 126.38(12.75,491.34) | 0.49(0.39,0.59) |
| Costa Rica | female | 118.50(81.95,164.48) | 267.51(173.97,393.84) | 2.87(2.71,3.03) | 40.34(33.40,48.30) | 67.82(59.92,76.52) | 1.55(1.38,1.73) |
| Croatia | female | 32.81(17.89,54.77) | 26.06(11.47,47.38) | -1.71(-2.16,-1.25) | 9.48(6.52,13.35) | 10.52(6.87,15.55) | -0.50(-0.87,-0.12) |
| Cuba | female | 912.62(642.32,1268.19) | 542.35(348.19,811.72) | -1.76(-2.45,-1.06) | 80.41(75.27,85.81) | 79.19(72.66,86.16) | -0.40(-0.83,0.03) |
| Cyprus | female | 6.13(3.05,10.67) | 13.33(5.31,24.95) | 3.13(2.68,3.59) | 9.86(3.66,21.57) | 16.45(8.82,28.61) | 1.57(1.37,1.76) |
| Czechia | female | 78.73(39.80,134.31) | 79.85(39.23,143.79) | -0.36(-0.87,0.15) | 11.24(8.89,14.02) | 16.93(13.42,21.09) | 1.27(1.13,1.41) |
| Côte d'Ivoire | female | 646.77(423.75,945.72) | 2263.78(1424.92,3502.99) | 4.48(3.71,5.26) | 54.30(50.19,58.65) | 93.02(89.23,96.94) | 2.07(1.58,2.58) |
| Democratic People's Republic of Korea | female | 737.41(451.14,1150.10) | 734.24(480.38,1084.41) | 0.36(0.10,0.61) | 34.89(32.41,37.51) | 37.18(34.53,39.98) | 0.18(0.10,0.26) |
| Democratic Republic of the Congo | female | 1961.04(1226.55,2820.95) | 4872.30(3276.46,6986.47) | 3.27(3.09,3.46) | 54.47(52.08,56.94) | 56.41(54.84,58.02) | 0.08(-0.09,0.24) |
| Denmark | female | 50.02(21.99,92.90) | 82.68(40.20,146.25) | 1.87(1.34,2.40) | 12.85(9.53,16.99) | 21.89(17.42,27.20) | 1.88(1.72,2.03) |
| Djibouti | female | 17.24(10.42,28.35) | 39.90(23.23,59.87) | 3.44(2.97,3.91) | 36.71(21.48,58.61) | 43.14(30.80,58.83) | 0.55(0.37,0.74) |
| Dominica | female | 12.25(9.33,15.77) | 10.31(7.41,13.91) | 0.01(-0.71,0.73) | 174.35(90.76,303.60) | 194.28(94.39,354.52) | 0.56(-0.20,1.32) |
| Dominican Republic | female | 905.31(670.89,1152.25) | 1316.12(919.49,1788.05) | 1.62(1.32,1.92) | 112.81(105.58,120.41) | 134.11(126.95,141.56) | 0.80(0.56,1.05) |
| Ecuador | female | 350.13(260.82,466.19) | 884.46(618.63,1219.26) | 2.48(1.90,3.06) | 34.23(30.74,38.02) | 54.70(51.15,58.42) | 0.95(0.41,1.50) |
| Egypt | female | 1123.09(701.20,1681.33) | 2552.62(1572.79,3913.53) | 3.63(3.38,3.87) | 22.05(20.78,23.38) | 28.97(27.86,30.12) | 1.52(1.19,1.86) |
| El Salvador | female | 271.26(187.99,376.06) | 501.62(347.30,707.65) | 2.10(1.90,2.31) | 48.81(43.17,54.99) | 80.36(73.46,87.74) | 1.71(1.46,1.96) |
| Equatorial Guinea | female | 22.54(14.47,34.47) | 81.29(50.44,119.08) | 4.70(4.47,4.93) | 56.39(35.54,85.12) | 58.35(46.35,72.53) | 0.15(0.03,0.27) |
| Eritrea | female | 132.27(73.40,242.93) | 345.34(219.35,520.57) | 3.57(3.36,3.77) | 46.37(38.79,55.01) | 49.76(44.65,55.31) | 0.32(0.22,0.43) |
| Estonia | female | 8.38(4.54,14.42) | 8.30(4.11,14.40) | 0.06(-0.65,0.77) | 8.06(3.56,15.66) | 13.30(5.84,26.10) | 1.60(1.18,2.01) |
| Eswatini | female | 59.45(40.92,83.04) | 126.19(73.09,192.15) | 3.38(2.31,4.46) | 68.21(51.96,88.03) | 106.76(88.95,127.11) | 2.31(1.53,3.10) |
| Ethiopia | female | 3859.16(2446.82,6541.21) | 3965.85(2601.52,5816.95) | -0.61(-0.89,-0.32) | 79.73(77.23,82.29) | 34.98(33.90,36.09) | -3.40(-3.59,-3.20) |
| Fiji | female | 101.89(71.27,146.72) | 184.64(139.84,240.07) | 1.32(0.58,2.07) | 139.45(113.69,169.31) | 249.40(214.72,288.12) | 1.25(0.65,1.85) |
| Finland | female | 41.27(17.10,78.11) | 136.55(69.32,224.34) | 4.23(3.50,4.97) | 12.02(8.63,16.37) | 44.51(37.35,52.67) | 4.16(3.36,4.96) |
| France | female | 230.75(124.30,372.29) | 322.87(94.05,644.70) | -0.01(-1.11,1.10) | 5.22(4.57,5.94) | 8.33(7.45,9.29) | 0.40(-0.71,1.53) |
| Gabon | female | 48.70(31.87,69.22) | 107.98(72.05,157.85) | 2.79(2.48,3.11) | 51.01(37.69,67.55) | 59.53(48.83,71.88) | 0.49(0.21,0.76) |
| Gambia | female | 37.94(22.75,57.66) | 155.02(106.04,214.31) | 4.69(4.20,5.18) | 38.57(27.28,52.98) | 63.17(53.61,73.97) | 1.59(1.17,2.02) |
| Georgia | female | 126.93(78.67,197.97) | 75.38(44.92,118.25) | -1.31(-1.70,-0.92) | 29.95(24.96,35.65) | 37.65(29.62,47.26) | 1.02(0.88,1.15) |
| Germany | female | 1237.99(679.43,1996.77) | 1701.47(890.99,2861.23) | 1.10(0.57,1.64) | 21.54(20.33,22.80) | 38.13(36.33,40.00) | 1.52(0.98,2.07) |
| Ghana | female | 989.29(687.24,1462.99) | 2333.33(1563.95,3304.82) | 3.13(2.96,3.30) | 68.00(63.82,72.37) | 71.97(69.08,74.95) | 0.14(-0.03,0.32) |
| Greece | female | 92.92(48.75,163.30) | 128.56(56.10,232.24) | 0.95(0.83,1.06) | 11.72(9.46,14.37) | 24.95(20.82,29.67) | 2.80(2.61,2.99) |
| Greenland | female | 0.33(0.20,0.50) | 0.28(0.07,0.66) | -2.41(-3.45,-1.36) | 6.25(0.00,119.76) | 6.91(0.00,118.11) | -2.85(-4.19,-1.49) |
| Grenada | female | 16.54(12.33,20.89) | 16.10(11.94,20.67) | -0.56(-0.92,-0.19) | 211.84(122.26,342.20) | 183.55(104.93,299.83) | -0.85(-1.03,-0.67) |
| Guam | female | 8.08(5.51,11.48) | 11.69(7.79,17.03) | 1.17(0.83,1.51) | 66.07(28.63,130.99) | 89.72(45.86,158.62) | 0.89(0.62,1.16) |
| Guatemala | female | 440.39(297.61,601.33) | 2475.47(1735.51,3396.48) | 5.82(5.17,6.47) | 59.12(53.72,64.93) | 131.96(126.81,137.27) | 2.33(1.84,2.83) |
| Guinea | female | 325.24(209.94,497.68) | 1242.14(847.59,1722.20) | 4.92(4.65,5.20) | 59.31(53.04,66.13) | 97.33(91.98,102.90) | 1.89(1.69,2.09) |
| Guinea-Bissau | female | 83.64(53.60,128.07) | 227.15(154.34,310.82) | 3.68(3.16,4.21) | 83.71(66.71,103.78) | 114.58(100.16,130.50) | 1.14(0.75,1.53) |
| Guyana | female | 173.22(125.84,221.93) | 188.97(136.56,249.43) | 0.07(-0.24,0.39) | 204.49(175.17,237.33) | 242.31(208.88,279.72) | 0.41(0.10,0.73) |
| Haiti | female | 2019.89(1298.47,3476.14) | 2596.58(1806.66,3807.44) | 1.09(0.88,1.30) | 334.15(319.72,349.07) | 219.16(210.81,227.76) | -1.32(-1.49,-1.15) |
| Honduras | female | 371.60(255.04,505.13) | 968.45(641.33,1391.88) | 3.29(3.09,3.48) | 82.98(74.73,91.92) | 92.14(86.42,98.13) | 0.22(0.07,0.36) |
| Hungary | female | 106.43(59.65,173.82) | 65.90(32.30,114.81) | -1.74(-2.03,-1.45) | 15.30(12.53,18.50) | 12.90(9.97,16.45) | -0.37(-0.53,-0.22) |
| Iceland | female | 2.43(0.90,4.43) | 5.88(2.74,10.27) | 3.32(3.07,3.57) | 11.38(1.82,37.77) | 25.14(9.10,56.19) | 2.67(2.46,2.87) |
| India | female | 39785.28(24860.05,67269.07) | 74244.26(48615.40,110952.47) | 1.58(1.19,1.98) | 51.52(51.02,52.03) | 58.24(57.82,58.66) | -0.26(-0.60,0.07) |
| Indonesia | female | 13701.87(10443.97,18970.94) | 14720.41(11675.84,18379.02) | -0.37(-0.77,0.03) | 72.28(71.08,73.50) | 66.97(65.89,68.06) | -0.65(-0.99,-0.31) |
| Iran (Islamic Republic of) | female | 1027.28(613.96,1561.17) | 1595.54(1047.82,2420.78) | 2.53(1.31,3.76) | 18.10(17.01,19.25) | 28.20(26.84,29.63) | 2.08(1.58,2.59) |
| Iraq | female | 3037.59(1972.08,4183.46) | 4694.58(3328.26,6387.82) | 0.95(0.44,1.45) | 187.36(180.72,194.18) | 109.70(106.58,112.89) | -2.22(-2.69,-1.76) |
| Ireland | female | 33.12(13.79,58.64) | 47.54(19.36,89.79) | 1.34(0.83,1.85) | 11.44(7.87,16.09) | 16.08(11.84,21.36) | 1.40(0.94,1.86) |
| Israel | female | 43.73(17.87,86.32) | 169.39(96.31,279.25) | 3.42(2.78,4.06) | 10.78(7.82,14.50) | 25.18(21.53,29.27) | 1.92(1.27,2.58) |
| Italy | female | 428.06(201.21,838.19) | 432.03(169.69,855.66) | -2.27(-5.41,0.97) | 9.45(8.57,10.39) | 15.01(13.62,16.49) | -0.72(-3.72,2.37) |
| Jamaica | female | 170.88(118.30,238.92) | 330.12(236.77,451.49) | 2.24(2.03,2.45) | 68.85(58.92,79.99) | 127.40(113.99,142.00) | 1.84(1.59,2.08) |
| Japan | female | 434.36(325.99,716.47) | 586.67(234.00,1156.48) | 0.19(-0.63,1.01) | 4.63(4.20,5.09) | 9.56(8.80,10.36) | 2.16(1.40,2.92) |
| Jordan | female | 210.35(146.46,293.83) | 368.08(230.56,562.53) | 1.40(1.09,1.71) | 53.04(46.10,60.75) | 35.17(31.67,38.96) | -1.90(-2.30,-1.50) |
| Kazakhstan | female | 568.71(358.05,843.00) | 597.92(377.34,899.31) | 1.44(0.81,2.08) | 42.10(38.71,45.71) | 48.05(44.27,52.08) | 1.05(0.67,1.43) |
| Kenya | female | 692.93(467.85,992.66) | 1711.52(1123.23,2380.60) | 3.08(2.95,3.21) | 28.95(26.83,31.19) | 32.06(30.56,33.62) | 0.40(0.33,0.47) |
| Kiribati | female | 14.23(10.38,18.97) | 27.79(19.90,37.14) | 2.63(2.27,3.00) | 192.87(105.92,324.22) | 252.78(167.68,366.07) | 0.70(0.38,1.02) |
| Kuwait | female | 84.01(61.41,111.05) | 137.18(86.07,203.05) | 2.00(1.70,2.30) | 53.36(42.50,66.31) | 49.23(41.28,58.38) | -0.93(-1.45,-0.41) |
| Kyrgyzstan | female | 108.61(69.57,161.21) | 162.18(100.00,247.50) | 1.51(1.25,1.76) | 27.43(22.51,33.11) | 28.98(24.68,33.83) | -0.13(-0.25,0.00) |
| Lao People's Democratic Republic | female | 507.39(302.03,924.43) | 627.66(414.27,881.23) | 0.69(0.32,1.08) | 126.74(115.93,138.30) | 90.16(83.24,97.50) | -1.39(-1.61,-1.18) |
| Latvia | female | 20.46(11.81,36.93) | 14.80(8.58,22.71) | -0.21(-1.06,0.65) | 11.24(6.91,17.32) | 17.35(9.67,28.74) | 1.83(1.47,2.19) |
| Lebanon | female | 89.03(54.73,131.00) | 119.26(70.58,186.41) | 1.95(1.54,2.36) | 31.88(25.60,39.25) | 35.62(29.51,42.65) | 0.65(0.52,0.77) |
| Lesotho | female | 75.91(49.05,106.24) | 197.11(128.02,288.75) | 4.43(3.90,4.96) | 41.48(32.66,52.00) | 92.52(80.05,106.38) | 4.10(3.57,4.64) |
| Liberia | female | 113.97(73.70,167.54) | 472.23(316.85,651.38) | 4.69(4.19,5.19) | 66.67(54.81,80.48) | 95.84(87.38,104.90) | 1.14(0.94,1.35) |
| Libya | female | 100.20(61.58,151.19) | 210.93(120.05,335.67) | 2.88(2.33,3.43) | 25.21(20.50,30.69) | 36.24(31.52,41.49) | 1.70(1.45,1.94) |
| Lithuania | female | 16.02(9.84,25.25) | 11.00(4.68,19.02) | -0.51(-1.26,0.24) | 5.80(3.32,9.44) | 6.96(3.47,12.70) | 1.12(0.52,1.72) |
| Luxembourg | female | 3.97(1.81,6.97) | 8.27(3.83,14.68) | 3.05(2.59,3.51) | 14.55(3.86,40.00) | 21.97(9.62,43.74) | 1.68(1.44,1.91) |
| Madagascar | female | 807.83(513.52,1261.12) | 1354.64(884.49,1943.30) | 1.88(1.57,2.19) | 69.48(64.77,74.46) | 49.99(47.36,52.73) | -1.02(-1.31,-0.74) |
| Malawi | female | 592.46(375.94,910.63) | 950.15(621.41,1413.33) | 1.01(0.70,1.32) | 61.64(56.77,66.81) | 46.92(43.97,50.02) | -1.41(-1.64,-1.18) |
| Malaysia | female | 838.49(573.39,1146.55) | 1266.75(833.49,1874.39) | 1.36(1.11,1.62) | 49.74(46.43,53.23) | 45.74(43.26,48.34) | -0.54(-0.72,-0.36) |
| Maldives | female | 10.48(6.25,17.82) | 10.24(6.60,15.29) | 0.68(0.38,0.97) | 47.86(23.41,87.03) | 33.33(16.10,61.70) | -1.04(-1.60,-0.48) |
| Mali | female | 609.69(383.15,937.84) | 2109.54(1389.42,3116.95) | 4.08(3.84,4.31) | 78.46(72.35,84.95) | 94.23(90.24,98.35) | 0.20(-0.07,0.47) |
| Malta | female | 6.99(3.73,11.21) | 9.49(5.10,16.10) | 0.38(-0.03,0.79) | 28.18(11.31,58.30) | 39.96(18.66,76.28) | 0.41(-0.10,0.92) |
| Marshall Islands | female | 5.29(3.71,7.30) | 11.38(7.65,16.30) | 2.19(1.45,2.94) | 127.23(42.80,293.71) | 228.40(115.51,405.77) | 1.86(1.47,2.24) |
| Mauritania | female | 119.02(77.99,186.63) | 269.89(164.39,406.79) | 2.81(2.49,3.14) | 60.39(50.02,72.30) | 64.12(56.68,72.27) | 0.23(-0.07,0.54) |
| Mauritius | female | 46.83(32.97,64.55) | 93.65(65.43,126.87) | 3.72(3.19,4.25) | 45.13(33.13,60.15) | 99.58(80.43,121.99) | 4.11(3.47,4.76) |
| Mexico | female | 10849.35(7675.57,13591.41) | 11401.93(8110.39,15892.21) | 0.15(-0.10,0.39) | 117.00(114.81,119.23) | 104.31(102.41,106.25) | -0.32(-0.59,-0.05) |
| Micronesia (Federated States of) | female | 9.77(6.82,13.68) | 19.59(13.19,27.43) | 2.07(1.50,2.65) | 101.84(48.05,190.92) | 198.69(120.60,308.80) | 2.24(1.78,2.69) |
| Monaco | female | 0.21(0.08,0.40) | 0.41(0.18,0.74) | 2.79(2.27,3.32) | 12.04(0.00,315.35) | 23.21(0.01,259.25) | 2.27(2.09,2.46) |
| Mongolia | female | 61.90(39.62,91.78) | 62.42(38.22,92.70) | -0.01(-0.44,0.42) | 28.56(21.89,36.64) | 26.56(20.36,34.11) | -0.64(-0.93,-0.35) |
| Montenegro | female | 9.42(5.64,14.32) | 7.82(4.74,12.46) | -0.61(-1.00,-0.22) | 18.73(8.75,35.07) | 19.42(8.29,38.81) | 0.23(-0.02,0.48) |
| Morocco | female | 625.14(377.45,939.65) | 1119.50(668.53,1715.46) | 2.29(1.86,2.72) | 24.50(22.62,26.50) | 36.96(34.82,39.19) | 1.65(1.43,1.88) |
| Mozambique | female | 583.04(361.17,897.01) | 1655.65(1115.60,2421.93) | 3.43(3.02,3.84) | 46.73(42.99,50.70) | 52.96(50.44,55.58) | 0.53(0.20,0.86) |
| Myanmar | female | 4309.26(2607.69,7585.06) | 3253.89(2182.81,4572.22) | -1.50(-1.74,-1.26) | 105.82(102.69,109.03) | 66.90(64.62,69.24) | -2.11(-2.32,-1.90) |
| Namibia | female | 70.34(43.71,101.05) | 122.05(76.98,176.09) | 1.81(1.43,2.20) | 46.85(36.53,59.22) | 51.30(42.60,61.25) | 0.19(-0.06,0.44) |
| Nauru | female | 1.07(0.77,1.48) | 2.00(1.39,2.87) | 1.94(1.51,2.36) | 116.25(3.65,623.16) | 195.66(23.73,711.70) | 1.66(1.31,2.02) |
| Nepal | female | 1097.54(626.38,2044.90) | 2376.26(1551.82,3516.03) | 2.62(2.48,2.76) | 59.10(55.65,62.70) | 72.00(69.13,74.96) | 0.54(0.33,0.75) |
| Netherlands | female | 95.70(36.39,187.25) | 150.72(59.95,294.88) | 2.85(1.92,3.79) | 7.73(6.25,9.46) | 14.27(12.08,16.75) | 3.22(2.58,3.86) |
| New Zealand | female | 14.56(8.57,28.75) | 44.83(18.31,82.14) | 3.72(3.53,3.91) | 5.11(2.83,8.49) | 15.44(11.25,20.70) | 3.33(3.03,3.63) |
| Nicaragua | female | 262.36(185.12,363.22) | 549.50(358.56,791.40) | 2.70(2.57,2.82) | 67.02(59.14,75.66) | 89.09(81.79,96.86) | 1.10(0.80,1.40) |
| Niger | female | 369.68(208.78,681.20) | 1569.05(971.83,2415.15) | 4.45(4.12,4.78) | 49.15(44.26,54.45) | 67.97(64.63,71.44) | 0.71(0.45,0.97) |
| Nigeria | female | 3110.22(1927.52,5178.10) | 7388.58(4664.62,11757.52) | 2.54(2.29,2.79) | 34.14(32.94,35.36) | 32.36(31.62,33.11) | -0.51(-0.74,-0.28) |
| Niue | female | 0.19(0.13,0.25) | 0.22(0.15,0.30) | 0.04(-0.22,0.30) | 106.81(0.00,2419.79) | 184.53(0.00,3541.87) | 1.92(1.72,2.12) |
| North Macedonia | female | 21.00(11.55,35.13) | 19.15(10.52,32.29) | -0.08(-0.46,0.31) | 12.52(7.75,19.17) | 13.86(8.35,21.86) | 0.31(0.10,0.52) |
| Northern Mariana Islands | female | 3.31(2.20,4.78) | 3.42(2.27,4.85) | -0.17(-1.50,1.18) | 65.01(14.62,190.60) | 95.16(22.35,264.67) | 1.42(1.26,1.57) |
| Norway | female | 60.09(27.44,108.93) | 82.19(37.69,146.63) | -0.90(-1.50,-0.29) | 18.33(13.99,23.64) | 24.85(19.77,30.88) | -1.17(-1.76,-0.58) |
| Oman | female | 38.48(24.98,56.99) | 110.09(70.52,163.57) | 3.68(3.14,4.23) | 29.70(21.05,40.77) | 39.80(32.70,48.05) | 1.17(0.85,1.48) |
| Pakistan | female | 5574.56(3664.07,8642.79) | 27185.79(18562.08,38803.42) | 5.53(5.44,5.62) | 55.67(54.21,57.15) | 122.67(121.21,124.14) | 2.78(2.70,2.86) |
| Palau | female | 1.29(0.89,1.78) | 1.53(1.05,2.17) | 0.53(0.04,1.02) | 86.83(4.42,411.24) | 149.14(11.10,637.65) | 1.87(1.71,2.04) |
| Palestine | female | 95.89(64.87,137.96) | 246.68(171.53,342.66) | 3.47(3.09,3.86) | 48.47(39.24,59.25) | 49.54(43.55,56.13) | -0.13(-0.62,0.36) |
| Panama | female | 106.80(73.22,149.35) | 277.68(186.32,389.05) | 3.06(2.92,3.20) | 44.17(36.19,53.38) | 79.77(70.66,89.74) | 1.87(1.75,2.00) |
| Papua New Guinea | female | 373.75(252.37,541.61) | 1357.61(972.12,1862.89) | 4.67(4.53,4.81) | 95.13(85.72,105.28) | 150.86(142.94,159.11) | 1.56(1.43,1.70) |
| Paraguay | female | 105.10(66.39,158.62) | 245.51(159.52,365.46) | 3.05(2.54,3.57) | 28.39(23.22,34.37) | 38.61(33.93,43.76) | 0.96(0.62,1.29) |
| Peru | female | 507.33(348.61,686.94) | 851.40(565.11,1219.69) | 1.81(1.62,2.00) | 22.79(20.85,24.86) | 30.64(28.62,32.77) | 1.00(0.77,1.23) |
| Philippines | female | 7011.89(5258.89,9026.46) | 7767.81(5964.32,10113.28) | -0.13(-0.41,0.16) | 109.55(107.00,112.15) | 75.71(74.03,77.41) | -1.78(-2.06,-1.50) |
| Poland | female | 360.38(239.38,525.66) | 284.69(154.51,474.46) | -0.20(-0.65,0.26) | 14.28(12.84,15.83) | 13.97(12.39,15.71) | 0.57(-0.22,1.36) |
| Portugal | female | 234.05(139.26,353.55) | 216.97(112.85,357.65) | -1.13(-1.46,-0.80) | 28.83(25.26,32.77) | 38.06(33.16,43.48) | 0.53(0.37,0.69) |
| Puerto Rico | female | 196.39(128.95,286.12) | 227.93(144.37,340.28) | 0.43(0.30,0.56) | 62.56(54.12,71.96) | 98.14(85.80,111.77) | 1.44(1.39,1.50) |
| Qatar | female | 10.47(7.26,15.17) | 39.80(25.01,61.74) | 5.92(5.25,6.60) | 45.50(22.24,82.81) | 44.23(31.56,60.43) | 0.16(-0.27,0.59) |
| Republic of Korea | female | 929.09(698.73,1238.14) | 671.41(396.43,1085.86) | -1.32(-1.57,-1.07) | 21.09(19.76,22.50) | 21.33(19.73,23.02) | -0.08(-0.45,0.29) |
| Republic of Moldova | female | 91.81(57.40,151.81) | 36.53(21.83,54.94) | -3.04(-3.64,-2.44) | 28.79(23.20,35.32) | 17.91(12.57,24.82) | -1.79(-2.01,-1.57) |
| Romania | female | 344.52(221.51,579.05) | 119.33(65.19,192.21) | -3.65(-3.95,-3.34) | 17.80(15.97,19.79) | 12.15(10.07,14.54) | -1.00(-1.21,-0.78) |
| Russian Federation | female | 1145.89(576.90,1961.39) | 492.61(221.00,937.06) | -3.32(-4.33,-2.31) | 11.41(10.76,12.09) | 6.91(6.32,7.55) | -2.30(-2.71,-1.90) |
| Rwanda | female | 480.30(310.89,752.39) | 536.25(336.34,816.62) | -0.06(-0.59,0.47) | 70.32(64.16,76.92) | 40.71(37.33,44.31) | -2.79(-3.15,-2.43) |
| Saint Kitts and Nevis | female | 4.25(3.10,5.65) | 4.68(3.15,6.55) | -0.16(-0.30,-0.01) | 106.96(30.63,268.14) | 103.91(32.12,250.83) | -0.84(-1.07,-0.60) |
| Saint Lucia | female | 27.95(21.27,36.03) | 24.39(17.53,32.25) | -0.19(-0.37,-0.02) | 193.28(128.35,279.73) | 176.79(113.65,263.10) | -0.13(-0.38,0.12) |
| Saint Vincent and the Grenadines | female | 26.05(19.98,32.61) | 19.25(14.45,24.64) | -1.53(-1.72,-1.34) | 229.74(150.06,337.11) | 218.18(131.83,340.14) | -0.59(-0.76,-0.42) |
| Samoa | female | 11.90(8.04,16.53) | 24.69(17.24,35.06) | 2.33(2.19,2.48) | 74.29(38.11,131.19) | 126.54(81.48,187.97) | 1.82(1.51,2.14) |
| San Marino | female | 0.25(0.09,0.50) | 0.51(0.23,0.91) | 2.39(2.14,2.64) | 12.22(0.00,220.22) | 23.43(0.03,229.05) | 2.40(2.26,2.54) |
| Sao Tome and Principe | female | 2.51(1.39,4.00) | 7.01(4.43,10.51) | 2.96(2.48,3.44) | 21.94(3.63,71.84) | 34.13(13.69,70.90) | 1.08(0.71,1.46) |
| Saudi Arabia | female | 821.57(551.86,1151.46) | 1555.92(1021.29,2280.27) | 2.73(2.44,3.01) | 58.78(54.82,62.95) | 56.49(53.71,59.40) | 0.04(-0.21,0.28) |
| Senegal | female | 485.43(315.03,701.32) | 1382.88(887.89,2006.88) | 3.70(3.26,4.15) | 64.57(58.94,70.60) | 92.27(87.47,97.28) | 1.34(1.00,1.68) |
| Serbia | female | 98.64(59.31,157.68) | 60.79(29.88,105.75) | -2.32(-2.57,-2.07) | 15.13(12.29,18.43) | 11.61(8.87,14.93) | -1.21(-1.33,-1.08) |
| Seychelles | female | 1.95(1.27,2.86) | 3.82(2.63,5.38) | 2.34(2.13,2.55) | 26.62(3.10,97.63) | 55.63(14.54,146.63) | 2.55(2.36,2.74) |
| Sierra Leone | female | 121.67(71.86,191.90) | 742.41(475.88,1096.57) | 7.32(6.69,7.95) | 35.02(29.07,41.83) | 82.87(77.02,89.06) | 3.33(2.80,3.86) |
| Singapore | female | 35.85(18.98,58.61) | 76.64(36.74,133.66) | 4.00(3.49,4.51) | 10.89(7.60,15.26) | 26.67(20.99,33.51) | 3.44(3.21,3.67) |
| Slovakia | female | 31.54(17.45,52.21) | 22.28(8.75,42.24) | -1.21(-1.68,-0.73) | 8.27(5.64,11.71) | 7.57(4.75,11.52) | -0.29(-0.37,-0.21) |
| Slovenia | female | 11.87(5.95,20.49) | 8.34(3.48,15.55) | -1.16(-1.57,-0.75) | 7.87(4.05,13.91) | 8.55(3.77,16.86) | 0.30(0.13,0.47) |
| Solomon Islands | female | 45.59(30.59,74.57) | 174.88(120.69,244.28) | 4.52(4.25,4.80) | 134.76(98.43,180.31) | 288.20(247.05,334.29) | 2.68(2.49,2.86) |
| Somalia | female | 319.54(189.63,538.70) | 1152.98(737.09,1740.82) | 3.84(3.50,4.18) | 56.75(50.57,63.50) | 58.45(55.11,61.93) | 0.13(0.08,0.19) |
| South Africa | female | 2934.35(2370.14,3643.26) | 2847.78(1831.19,3985.61) | 0.68(-0.34,1.70) | 73.88(71.23,76.60) | 62.25(59.98,64.58) | -0.01(-0.75,0.74) |
| South Sudan | female | 245.16(144.49,446.72) | 407.13(250.58,639.93) | 1.53(1.30,1.76) | 42.27(37.14,47.92) | 43.67(39.51,48.16) | 0.02(-0.15,0.20) |
| Spain | female | 344.02(195.23,593.61) | 563.08(265.53,1029.31) | 2.13(1.61,2.66) | 10.62(9.53,11.81) | 25.54(23.47,27.74) | 3.69(2.99,4.40) |
| Sri Lanka | female | 644.57(453.68,902.76) | 1100.35(780.21,1555.11) | 1.63(1.41,1.86) | 38.96(36.01,42.08) | 65.67(61.85,69.67) | 1.74(1.63,1.86) |
| Sudan | female | 510.49(287.65,903.63) | 1328.93(792.31,2114.61) | 3.70(3.48,3.92) | 25.83(23.64,28.17) | 32.86(31.11,34.67) | 1.23(1.05,1.41) |
| Suriname | female | 49.90(34.50,65.62) | 76.46(55.40,101.99) | 1.55(1.18,1.92) | 129.93(96.40,171.46) | 167.79(132.29,209.95) | 0.61(0.16,1.05) |
| Sweden | female | 82.11(29.25,162.06) | 98.91(39.35,199.02) | 0.98(0.39,1.57) | 14.09(11.20,17.51) | 17.95(14.58,21.86) | 0.55(0.06,1.04) |
| Switzerland | female | 51.49(18.12,102.04) | 81.80(32.40,154.79) | 1.65(1.02,2.28) | 10.28(7.65,13.59) | 16.82(13.36,20.94) | 1.22(0.66,1.78) |
| Syrian Arab Republic | female | 978.02(666.82,1338.74) | 845.08(573.23,1185.74) | -1.50(-2.10,-0.90) | 78.66(73.79,83.76) | 52.23(48.76,55.88) | -1.84(-2.18,-1.50) |
| Taiwan (Province of China) | female | 529.35(358.69,767.03) | 332.23(210.58,493.25) | -2.14(-2.53,-1.75) | 28.64(26.25,31.19) | 23.36(20.90,26.05) | -1.19(-1.46,-0.91) |
| Tajikistan | female | 305.72(226.82,409.79) | 740.54(492.95,995.66) | 2.97(2.60,3.35) | 58.87(52.45,65.86) | 86.05(79.96,92.49) | 0.71(0.21,1.22) |
| Thailand | female | 2257.00(1550.19,3116.28) | 1880.05(1293.93,2645.31) | -1.83(-2.34,-1.32) | 38.29(36.73,39.90) | 40.03(38.24,41.90) | -1.04(-1.55,-0.53) |
| Timor-Leste | female | 44.31(21.44,93.60) | 73.53(43.25,107.28) | 1.22(0.47,1.98) | 62.01(45.09,83.29) | 51.60(40.47,64.88) | -1.36(-2.02,-0.70) |
| Togo | female | 178.91(115.13,266.04) | 485.41(318.96,689.46) | 3.50(3.18,3.81) | 49.30(42.34,57.11) | 65.66(59.95,71.78) | 1.02(0.86,1.19) |
| Tokelau | female | 0.12(0.08,0.18) | 0.17(0.12,0.25) | 0.38(-0.09,0.85) | 97.76(0.00,3244.39) | 157.29(0.00,3789.18) | 1.59(1.41,1.77) |
| Tonga | female | 7.81(5.51,10.58) | 10.41(7.44,14.43) | 0.98(0.87,1.10) | 81.90(34.78,164.15) | 112.54(54.86,205.27) | 1.15(1.06,1.24) |
| Trinidad and Tobago | female | 104.24(75.36,143.66) | 94.85(67.38,134.37) | -0.45(-1.05,0.15) | 97.34(79.55,117.93) | 107.82(87.22,131.87) | 0.13(-0.03,0.30) |
| Tunisia | female | 150.25(88.79,235.73) | 235.41(131.79,368.22) | 1.83(1.42,2.24) | 18.02(15.25,21.14) | 28.66(25.12,32.58) | 1.71(1.64,1.78) |
| Turkey | female | 2273.60(1463.49,3165.09) | 1459.25(858.90,2347.85) | -1.76(-2.32,-1.20) | 39.22(37.62,40.86) | 22.14(21.02,23.31) | -2.02(-2.54,-1.50) |
| Turkmenistan | female | 145.33(93.59,200.86) | 303.70(202.52,437.23) | 3.11(2.64,3.59) | 40.87(34.49,48.09) | 78.86(70.23,88.26) | 2.57(2.28,2.87) |
| Tuvalu | female | 0.95(0.67,1.34) | 1.69(1.18,2.33) | 2.29(2.17,2.41) | 117.20(2.50,694.00) | 162.44(14.76,648.08) | 1.10(0.94,1.27) |
| Uganda | female | 688.84(405.84,1044.44) | 2199.72(1399.83,3278.13) | 3.45(3.03,3.88) | 38.74(35.90,41.75) | 50.47(48.38,52.63) | 0.26(-0.11,0.64) |
| Ukraine | female | 702.74(398.91,1112.57) | 369.95(201.70,603.51) | -2.84(-3.47,-2.21) | 20.06(18.61,21.60) | 17.06(15.36,18.90) | -1.50(-2.00,-0.98) |
| United Arab Emirates | female | 37.99(24.63,55.66) | 115.82(69.95,178.18) | 4.02(2.82,5.24) | 33.46(23.65,46.06) | 36.98(30.55,44.36) | 0.67(0.40,0.95) |
| United Kingdom | female | 963.63(512.82,1587.86) | 2007.03(1116.95,3240.86) | 6.26(4.96,7.58) | 22.24(20.85,23.70) | 49.85(47.69,52.09) | 5.96(4.67,7.27) |
| United Republic of Tanzania | female | 1014.77(637.39,1525.71) | 2554.60(1681.82,3703.84) | 3.01(2.77,3.24) | 37.66(35.37,40.06) | 43.91(42.22,45.65) | 0.47(0.27,0.68) |
| United States of America | female | 2194.23(1172.04,3741.03) | 7492.55(4058.04,12047.17) | 4.53(4.21,4.84) | 11.53(11.05,12.03) | 35.10(34.31,35.90) | 3.81(3.51,4.10) |
| United States Virgin Islands | female | 6.52(4.67,8.88) | 5.51(3.66,7.92) | -0.72(-0.83,-0.60) | 75.36(28.99,160.77) | 90.54(31.43,203.74) | 0.39(0.24,0.54) |
| Uruguay | female | 41.22(26.37,57.28) | 35.60(23.33,50.68) | -0.81(-1.27,-0.34) | 16.94(12.17,22.97) | 13.95(9.74,19.38) | -0.90(-1.39,-0.40) |
| Uzbekistan | female | 541.96(321.08,838.14) | 1437.16(972.34,2084.23) | 3.22(2.62,3.83) | 27.22(24.98,29.61) | 49.19(46.67,51.80) | 1.47(1.02,1.92) |
| Vanuatu | female | 8.84(5.81,12.78) | 33.01(22.31,45.53) | 4.78(4.57,4.99) | 62.54(28.34,119.68) | 118.47(81.55,166.43) | 2.23(2.10,2.36) |
| Venezuela (Bolivarian Republic of) | female | 1267.96(951.01,1650.36) | 2692.06(1922.60,3760.35) | 3.16(2.59,3.73) | 67.48(63.82,71.30) | 124.18(119.53,128.96) | 2.24(1.56,2.93) |
| Viet Nam | female | 2078.13(1357.40,3094.99) | 2371.99(1601.08,3348.85) | 0.89(0.68,1.10) | 30.07(28.79,31.40) | 33.92(32.57,35.32) | 0.44(0.11,0.77) |
| Yemen | female | 165.53(79.40,320.05) | 596.63(300.97,1021.35) | 4.39(4.04,4.74) | 15.34(13.08,17.87) | 20.87(19.23,22.62) | 0.88(0.44,1.33) |
| Zambia | female | 574.71(385.07,812.72) | 1018.82(654.92,1475.83) | 1.65(1.52,1.77) | 65.93(60.64,71.57) | 51.97(48.83,55.27) | -1.21(-1.38,-1.04) |
| Zimbabwe | female | 583.66(403.67,801.62) | 1388.33(941.45,1939.07) | 3.83(3.38,4.28) | 52.67(48.47,57.14) | 88.32(83.73,93.10) | 2.95(2.41,3.48) |
| Afghanistan | male | 220.83(122.28,351.41) | 1111.35(616.25,1812.15) | 4.82(4.08,5.57) | 19.93(17.37,22.78) | 27.58(25.99,29.26) | 1.19(1.12,1.27) |
| Albania | male | 21.87(10.18,40.52) | 15.91(5.74,29.55) | -1.49(-1.85,-1.13) | 6.80(4.26,10.32) | 6.93(3.95,11.40) | -0.65(-0.97,-0.32) |
| Algeria | male | 525.15(301.85,805.36) | 818.15(427.24,1394.86) | 1.74(1.23,2.25) | 20.07(18.39,21.86) | 24.66(23.00,26.42) | 0.70(0.59,0.82) |
| American Samoa | male | 5.92(3.95,8.57) | 9.85(6.34,14.88) | 1.29(0.99,1.60) | 122.06(44.43,267.15) | 183.56(87.32,340.27) | 1.37(1.07,1.67) |
| Andorra | male | 0.39(0.10,0.84) | 0.56(0.17,1.16) | 0.61(0.07,1.15) | 8.18(0.00,106.56) | 12.13(0.02,110.40) | 1.37(1.15,1.59) |
| Angola | male | 785.49(442.43,1279.39) | 2110.91(1370.64,3177.24) | 3.52(3.29,3.76) | 81.58(75.96,87.50) | 79.70(76.32,83.18) | 0.01(-0.19,0.21) |
| Antigua and Barbuda | male | 3.16(2.19,4.35) | 5.86(4.02,8.25) | 1.60(1.21,2.00) | 55.15(12.01,158.43) | 80.30(29.00,178.26) | 0.58(0.16,1.01) |
| Argentina | male | 102.93(60.35,157.90) | 389.26(196.79,651.73) | 4.39(4.05,4.73) | 3.86(3.15,4.68) | 10.61(9.59,11.72) | 3.35(3.03,3.66) |
| Armenia | male | 53.81(30.10,85.79) | 50.19(30.87,76.29) | -0.21(-0.82,0.40) | 18.89(14.19,24.67) | 25.97(19.28,34.33) | 0.56(0.21,0.90) |
| Australia | male | 50.88(23.51,93.99) | 57.54(14.48,155.56) | -0.14(-0.77,0.49) | 3.69(2.74,4.85) | 3.55(2.69,4.61) | -0.81(-1.29,-0.32) |
| Austria | male | 28.69(8.77,58.39) | 35.38(6.41,83.03) | 0.88(0.34,1.43) | 4.23(2.82,6.16) | 6.29(4.39,8.79) | 1.01(0.25,1.78) |
| Azerbaijan | male | 173.51(106.39,263.94) | 251.18(156.13,385.49) | 0.34(-0.53,1.23) | 24.73(21.19,28.70) | 31.59(27.79,35.78) | -0.93(-1.84,-0.02) |
| Bahamas | male | 13.54(9.51,18.68) | 25.99(17.94,36.53) | 2.72(2.50,2.95) | 50.24(27.14,85.15) | 83.44(54.49,122.54) | 2.03(1.82,2.23) |
| Bahrain | male | 15.24(10.32,21.80) | 59.24(41.51,84.45) | 6.03(5.09,6.98) | 33.22(18.47,55.91) | 69.17(52.69,89.19) | 2.98(2.68,3.28) |
| Bangladesh | male | 5001.15(2806.35,7573.59) | 6195.61(3889.60,9300.41) | 0.47(0.11,0.83) | 48.02(46.69,49.37) | 43.61(42.53,44.71) | -0.67(-0.94,-0.40) |
| Barbados | male | 14.05(10.10,19.10) | 15.83(10.76,22.07) | -0.11(-0.29,0.06) | 60.33(33.01,101.50) | 80.49(45.85,131.07) | 0.33(0.03,0.63) |
| Belarus | male | 59.92(32.42,101.54) | 23.53(6.38,48.29) | -3.27(-4.13,-2.39) | 8.24(6.29,10.61) | 4.84(3.09,7.27) | -2.41(-2.81,-2.00) |
| Belgium | male | 59.30(20.31,118.27) | 81.69(26.26,170.67) | 1.32(0.91,1.74) | 7.85(5.98,10.15) | 11.90(9.46,14.80) | 1.36(1.11,1.61) |
| Belize | male | 7.74(5.56,10.57) | 34.43(23.95,46.45) | 4.43(3.79,5.08) | 41.67(17.66,83.49) | 83.59(58.02,116.65) | 1.46(0.80,2.12) |
| Benin | male | 169.25(103.80,245.11) | 650.03(410.90,990.30) | 4.64(4.23,5.05) | 41.49(35.46,48.30) | 52.81(48.82,57.04) | 0.67(0.29,1.04) |
| Bermuda | male | 1.41(0.93,2.09) | 1.22(0.77,1.82) | -0.33(-0.50,-0.16) | 30.40(1.86,145.59) | 40.11(1.81,196.20) | 0.87(0.81,0.93) |
| Bhutan | male | 21.82(12.60,33.18) | 34.62(21.71,51.94) | 2.63(2.35,2.90) | 29.18(18.24,44.27) | 43.56(30.23,61.01) | 1.54(1.44,1.64) |
| Bolivia (Plurinational State of) | male | 143.38(93.51,205.12) | 334.93(213.45,501.34) | 3.03(2.80,3.26) | 24.56(20.70,28.95) | 30.81(27.60,34.30) | 0.67(0.61,0.73) |
| Bosnia and Herzegovina | male | 40.64(22.24,69.29) | 54.10(34.87,78.56) | 2.35(1.60,3.10) | 9.51(6.81,12.94) | 25.30(18.99,33.12) | 4.05(3.63,4.48) |
| Botswana | male | 88.49(59.01,129.78) | 198.39(126.39,307.34) | 1.75(0.85,2.67) | 70.35(56.40,86.79) | 94.40(81.72,108.50) | 0.06(-0.58,0.70) |
| Brazil | male | 6469.88(4674.76,8137.20) | 7018.54(4812.19,9840.38) | 0.54(0.35,0.74) | 44.95(43.87,46.06) | 40.08(39.14,41.03) | -0.13(-0.53,0.27) |
| Brunei Darussalam | male | 19.24(12.19,30.47) | 18.32(10.87,28.24) | -0.27(-0.62,0.08) | 71.93(43.43,112.58) | 43.66(25.99,69.20) | -2.06(-2.48,-1.63) |
| Bulgaria | male | 86.96(51.54,139.73) | 39.26(20.48,69.34) | -3.37(-3.75,-2.99) | 14.26(11.42,17.59) | 12.16(8.66,16.61) | -1.24(-1.49,-0.99) |
| Burkina Faso | male | 389.98(204.09,643.16) | 1352.83(732.93,2183.60) | 4.23(4.08,4.39) | 46.04(41.55,50.90) | 62.22(58.93,65.64) | 0.93(0.79,1.07) |
| Burundi | male | 337.57(201.73,534.53) | 566.84(350.61,843.57) | 2.01(1.83,2.20) | 71.07(63.68,79.09) | 53.37(49.06,57.97) | -1.35(-1.50,-1.21) |
| Cabo Verde | male | 6.52(3.69,10.03) | 22.05(14.57,31.91) | 4.04(3.15,4.92) | 18.83(7.26,40.06) | 43.19(27.07,65.50) | 2.23(1.74,2.72) |
| Cambodia | male | 792.17(412.43,1411.96) | 1002.67(694.14,1399.19) | 0.99(0.84,1.14) | 85.93(80.05,92.14) | 65.04(61.08,69.20) | -1.24(-1.41,-1.07) |
| Cameroon | male | 432.93(256.75,667.01) | 1949.08(1231.72,2877.15) | 5.15(4.79,5.51) | 44.57(40.46,49.01) | 64.52(61.68,67.45) | 1.16(0.91,1.41) |
| Canada | male | 22.23(10.22,32.75) | 57.86(13.19,233.96) | 1.51(0.50,2.52) | 1.10(0.69,1.66) | 2.60(1.97,3.36) | 0.90(-0.17,1.97) |
| Central African Republic | male | 239.55(149.07,359.71) | 507.88(320.89,747.03) | 2.43(2.19,2.67) | 94.69(83.07,107.50) | 98.48(90.08,107.45) | 0.10(0.02,0.18) |
| Chad | male | 204.92(126.47,308.01) | 857.54(547.14,1294.28) | 4.91(4.53,5.28) | 37.74(32.73,43.32) | 53.64(50.09,57.39) | 1.14(0.69,1.59) |
| Chile | male | 26.32(15.38,58.69) | 86.70(25.85,187.84) | 3.83(3.44,4.22) | 2.07(1.35,3.02) | 6.20(4.96,7.67) | 3.15(2.69,3.62) |
| China | male | 36750.42(23249.54,57119.45) | 31362.55(18863.33,49550.17) | 2.64(1.57,3.71) | 26.93(26.65,27.20) | 36.37(35.97,36.78) | 3.44(2.75,4.14) |
| Colombia | male | 1750.00(1265.03,2344.10) | 2518.33(1615.04,3710.86) | 0.78(0.61,0.95) | 54.61(52.08,57.23) | 60.24(57.91,62.64) | -0.23(-0.43,-0.03) |
| Comoros | male | 18.88(8.51,30.99) | 28.23(17.01,44.18) | 0.76(0.24,1.28) | 42.60(25.55,66.93) | 41.06(27.33,59.29) | -0.67(-1.16,-0.17) |
| Congo | male | 239.97(148.91,368.95) | 314.20(198.32,475.58) | 0.35(0.07,0.62) | 100.27(87.96,113.83) | 69.12(61.68,77.22) | -1.62(-1.87,-1.37) |
| Cook Islands | male | 1.44(0.99,2.12) | 1.52(1.04,2.15) | -0.30(-0.60,0.00) | 78.30(5.15,348.12) | 121.31(8.92,516.83) | 1.29(1.14,1.45) |
| Costa Rica | male | 92.86(57.45,138.90) | 243.38(152.03,363.91) | 3.52(3.18,3.86) | 31.83(25.68,39.00) | 63.13(55.43,71.62) | 2.30(2.24,2.36) |
| Croatia | male | 38.82(21.18,65.97) | 29.86(12.93,55.66) | -1.45(-1.86,-1.04) | 10.87(7.72,14.89) | 11.58(7.80,16.67) | -0.29(-0.61,0.02) |
| Cuba | male | 646.34(437.27,932.59) | 490.97(305.46,740.08) | -1.00(-1.58,-0.41) | 54.40(50.28,58.76) | 66.76(60.98,72.95) | 0.31(0.00,0.62) |
| Cyprus | male | 5.03(2.19,9.20) | 8.53(2.26,18.08) | 2.38(1.70,3.06) | 7.46(2.43,17.66) | 9.79(4.35,19.61) | 0.72(0.36,1.08) |
| Czechia | male | 101.92(53.92,169.30) | 92.69(45.16,160.27) | -0.70(-1.30,-0.10) | 13.95(11.37,16.95) | 18.70(15.09,22.93) | 0.89(0.65,1.13) |
| Côte d'Ivoire | male | 566.11(358.19,843.54) | 1396.13(884.51,2084.64) | 2.10(1.36,2.84) | 48.78(44.85,52.98) | 53.85(51.06,56.75) | -0.31(-0.79,0.17) |
| Democratic People's Republic of Korea | male | 740.43(423.51,1202.06) | 990.49(631.35,1515.54) | 1.56(1.33,1.80) | 42.80(39.77,46.01) | 47.83(44.89,50.92) | 0.27(0.19,0.35) |
| Democratic Republic of the Congo | male | 2538.49(1554.95,4079.74) | 6369.44(4105.22,9700.86) | 3.28(3.22,3.34) | 70.63(67.91,73.44) | 73.24(71.45,75.07) | 0.06(-0.01,0.13) |
| Denmark | male | 32.08(9.30,66.80) | 46.06(16.10,93.89) | 1.62(1.07,2.17) | 7.75(5.30,10.98) | 11.52(8.43,15.43) | 1.63(1.45,1.82) |
| Djibouti | male | 25.68(15.36,40.23) | 65.82(39.82,98.43) | 3.85(3.53,4.17) | 43.09(28.06,63.29) | 57.75(44.64,73.49) | 1.00(0.82,1.18) |
| Dominica | male | 4.90(3.43,6.80) | 6.15(4.16,8.59) | 1.05(0.67,1.44) | 65.78(21.01,155.67) | 108.17(40.31,233.54) | 1.71(1.23,2.20) |
| Dominican Republic | male | 420.94(308.40,565.94) | 963.92(625.74,1500.38) | 3.23(3.01,3.45) | 57.02(51.71,62.74) | 97.21(91.17,103.55) | 1.99(1.75,2.23) |
| Ecuador | male | 232.15(160.24,332.17) | 838.26(572.16,1173.21) | 4.04(3.48,4.60) | 23.67(20.72,26.93) | 50.64(47.27,54.19) | 2.27(1.71,2.82) |
| Egypt | male | 850.24(421.86,1383.56) | 3694.54(2114.45,5748.58) | 6.13(5.83,6.43) | 15.63(14.60,16.72) | 40.13(38.85,41.44) | 4.10(3.73,4.47) |
| El Salvador | male | 234.49(160.20,334.81) | 467.05(314.49,673.82) | 2.89(2.63,3.15) | 46.12(40.39,52.45) | 77.62(70.74,85.01) | 2.25(1.89,2.61) |
| Equatorial Guinea | male | 35.26(20.44,55.78) | 151.78(96.74,227.63) | 5.36(4.95,5.77) | 99.41(69.23,138.52) | 78.05(66.12,91.54) | -1.00(-1.42,-0.58) |
| Eritrea | male | 177.36(98.17,287.01) | 433.20(270.72,656.15) | 2.97(2.60,3.33) | 59.62(51.15,69.11) | 60.40(54.84,66.37) | -0.18(-0.40,0.05) |
| Estonia | male | 10.07(5.19,17.76) | 5.55(2.36,10.70) | -1.37(-2.14,-0.59) | 8.99(4.32,16.51) | 8.36(2.92,18.97) | -0.11(-0.54,0.32) |
| Eswatini | male | 61.39(41.07,85.94) | 219.74(127.33,344.37) | 4.52(3.21,5.84) | 81.35(62.14,104.82) | 189.13(164.93,215.89) | 3.16(2.09,4.23) |
| Ethiopia | male | 4063.09(2207.63,6244.34) | 4279.11(2769.15,6330.65) | -0.28(-0.49,-0.08) | 88.41(85.71,91.18) | 37.59(36.47,38.73) | -3.24(-3.43,-3.05) |
| Fiji | male | 69.29(47.39,97.53) | 129.23(92.41,173.35) | 2.09(1.64,2.55) | 93.21(72.56,117.92) | 166.23(138.80,197.51) | 1.89(1.63,2.15) |
| Finland | male | 36.16(14.24,67.25) | 98.23(46.74,175.00) | 3.16(1.65,4.68) | 10.17(7.12,14.14) | 30.23(24.55,36.88) | 3.04(1.48,4.63) |
| France | male | 138.63(54.70,270.85) | 126.59(21.71,365.96) | -2.56(-4.21,-0.88) | 3.06(2.57,3.62) | 3.15(2.63,3.75) | -2.14(-3.80,-0.46) |
| Gabon | male | 103.60(63.96,161.13) | 169.37(108.75,248.08) | 1.58(1.47,1.68) | 114.15(93.22,138.40) | 103.85(88.80,120.72) | -0.55(-0.69,-0.40) |
| Gambia | male | 30.29(16.19,51.86) | 117.14(67.46,178.47) | 4.28(3.91,4.66) | 32.30(21.83,46.17) | 49.31(40.77,59.15) | 1.22(0.91,1.53) |
| Georgia | male | 99.42(57.67,156.98) | 75.81(45.60,119.28) | -0.55(-0.92,-0.17) | 23.48(19.09,28.58) | 34.01(26.78,42.64) | 1.47(1.25,1.68) |
| Germany | male | 1074.20(574.43,1794.19) | 1409.73(658.35,2527.31) | 0.75(0.24,1.27) | 17.72(16.65,18.83) | 27.51(26.08,28.99) | 1.00(0.53,1.47) |
| Ghana | male | 516.99(327.69,756.19) | 2211.22(1409.25,3251.90) | 5.91(5.52,6.31) | 35.35(32.37,38.55) | 69.72(66.85,72.69) | 2.98(2.64,3.32) |
| Greece | male | 66.62(28.90,126.02) | 75.76(27.62,153.85) | 0.27(-0.10,0.64) | 8.19(6.35,10.42) | 14.35(11.30,17.98) | 2.13(2.03,2.24) |
| Greenland | male | 0.26(0.14,0.46) | 0.31(0.05,0.75) | 1.87(0.81,2.95) | 4.34(0.00,104.69) | 7.54(0.00,119.90) | 1.72(1.03,2.41) |
| Grenada | male | 4.74(3.34,6.41) | 8.76(6.24,12.03) | 2.25(1.88,2.63) | 59.24(18.45,142.16) | 88.73(39.96,172.13) | 1.66(1.49,1.84) |
| Guam | male | 9.30(5.89,13.91) | 13.24(8.20,20.20) | 1.60(1.22,1.99) | 56.51(26.03,109.60) | 87.83(47.01,150.92) | 1.62(1.53,1.70) |
| Guatemala | male | 285.76(193.32,405.91) | 1716.15(1227.20,2333.55) | 6.35(5.71,7.01) | 41.36(36.69,46.47) | 90.20(85.99,94.58) | 2.42(1.87,2.97) |
| Guinea | male | 177.21(107.06,267.32) | 605.39(383.16,908.15) | 4.20(3.96,4.45) | 33.28(28.55,38.60) | 50.95(46.96,55.20) | 1.54(1.31,1.77) |
| Guinea-Bissau | male | 70.95(42.26,112.26) | 133.60(81.74,197.88) | 2.06(1.73,2.39) | 71.65(55.92,90.67) | 68.73(57.56,81.47) | -0.27(-0.49,-0.04) |
| Guyana | male | 71.17(50.99,95.75) | 104.67(73.16,144.22) | 1.46(1.18,1.74) | 87.32(68.22,110.14) | 130.24(106.43,157.95) | 1.63(1.43,1.83) |
| Haiti | male | 280.60(175.13,418.58) | 636.92(400.49,956.56) | 3.11(2.98,3.24) | 51.20(45.37,57.58) | 55.95(51.68,60.47) | 0.41(0.13,0.70) |
| Honduras | male | 254.03(172.71,356.31) | 771.42(497.41,1139.14) | 3.80(3.55,4.05) | 61.75(54.34,69.91) | 76.73(71.41,82.35) | 0.69(0.55,0.82) |
| Hungary | male | 104.73(54.22,180.37) | 69.50(31.80,125.63) | -1.72(-2.10,-1.34) | 14.41(11.78,17.45) | 12.84(10.00,16.26) | -0.40(-0.56,-0.23) |
| Iceland | male | 1.75(0.47,3.70) | 3.70(1.41,7.30) | 3.08(2.76,3.39) | 7.95(0.77,31.56) | 14.78(3.75,40.86) | 2.30(2.05,2.54) |
| India | male | 25990.96(15923.52,39108.04) | 64406.27(40811.22,96819.09) | 3.02(2.75,3.28) | 31.26(30.88,31.64) | 46.97(46.61,47.34) | 1.07(0.86,1.28) |
| Indonesia | male | 9166.89(6915.63,11910.93) | 15699.95(11834.91,20584.77) | 1.50(1.19,1.80) | 50.96(49.92,52.01) | 69.70(68.61,70.80) | 0.94(0.65,1.23) |
| Iran (Islamic Republic of) | male | 859.82(483.26,1378.15) | 1380.45(824.86,2210.28) | 2.56(1.24,3.90) | 14.79(13.82,15.82) | 23.25(22.04,24.51) | 1.93(1.31,2.55) |
| Iraq | male | 892.21(586.35,1260.39) | 2357.89(1544.07,3490.78) | 2.95(2.67,3.23) | 53.08(49.64,56.70) | 51.96(49.88,54.10) | -0.22(-0.42,-0.02) |
| Ireland | male | 23.90(7.10,48.01) | 29.73(8.86,64.47) | 0.89(0.25,1.54) | 7.91(5.05,11.81) | 9.81(6.60,14.02) | 1.10(0.39,1.81) |
| Israel | male | 20.49(5.56,46.94) | 97.68(52.30,168.80) | 4.25(3.43,5.07) | 4.93(3.03,7.59) | 13.98(11.35,17.04) | 2.72(2.06,3.38) |
| Italy | male | 371.78(140.35,790.72) | 401.30(144.72,818.23) | -1.44(-3.74,0.92) | 7.90(7.12,8.75) | 12.82(11.60,14.14) | 0.00(-2.16,2.20) |
| Jamaica | male | 72.48(45.70,108.08) | 184.33(125.44,262.74) | 2.91(2.55,3.27) | 30.36(23.77,38.22) | 68.70(59.11,79.43) | 2.19(1.78,2.60) |
| Japan | male | 395.32(224.15,825.35) | 770.58(305.20,1539.11) | 1.29(0.67,1.91) | 4.07(3.68,4.49) | 11.92(11.10,12.80) | 3.31(2.78,3.84) |
| Jordan | male | 122.50(77.32,179.17) | 439.67(252.90,703.05) | 4.90(4.50,5.31) | 26.43(21.96,31.55) | 35.94(32.66,39.47) | 1.37(1.05,1.70) |
| Kazakhstan | male | 308.41(174.99,482.46) | 301.61(168.36,484.28) | 0.03(-0.46,0.52) | 22.04(19.65,24.65) | 23.80(21.18,26.65) | -0.27(-0.67,0.13) |
| Kenya | male | 699.37(431.75,986.91) | 2365.58(1605.30,3278.96) | 4.32(3.83,4.82) | 31.73(29.42,34.19) | 44.93(43.13,46.78) | 1.37(0.98,1.77) |
| Kiribati | male | 10.33(7.05,14.79) | 32.55(20.85,49.10) | 4.43(3.73,5.13) | 140.58(68.35,257.17) | 303.18(208.11,426.76) | 2.29(1.61,2.97) |
| Kuwait | male | 65.59(41.01,98.59) | 158.88(94.57,246.26) | 2.55(1.89,3.22) | 37.53(28.86,48.24) | 53.06(45.08,62.15) | -0.25(-0.87,0.37) |
| Kyrgyzstan | male | 94.43(58.78,139.75) | 119.05(67.92,187.33) | 0.64(0.37,0.92) | 23.46(18.96,28.71) | 20.76(17.19,24.87) | -0.97(-1.22,-0.72) |
| Lao People's Democratic Republic | male | 386.67(227.00,628.20) | 512.90(347.10,748.09) | 0.78(0.53,1.02) | 107.55(97.05,118.90) | 72.88(66.71,79.47) | -1.71(-1.90,-1.52) |
| Latvia | male | 23.01(14.44,36.25) | 11.87(6.42,18.00) | -1.71(-2.48,-0.94) | 12.08(7.65,18.15) | 13.16(6.77,23.10) | 0.44(0.12,0.77) |
| Lebanon | male | 66.84(37.44,105.33) | 116.18(63.46,192.48) | 2.88(2.47,3.29) | 22.29(17.26,28.34) | 30.61(25.29,36.74) | 1.36(1.25,1.48) |
| Lesotho | male | 90.52(60.71,129.51) | 260.46(163.36,390.35) | 4.48(3.91,5.05) | 52.53(42.22,64.65) | 121.16(106.89,136.80) | 3.91(3.47,4.35) |
| Liberia | male | 68.99(40.99,105.22) | 249.54(157.19,377.52) | 4.07(3.61,4.52) | 42.40(32.76,54.36) | 50.92(44.78,57.67) | 0.40(0.17,0.63) |
| Libya | male | 83.67(45.60,136.81) | 186.22(98.64,307.84) | 3.16(2.51,3.82) | 19.38(15.45,24.03) | 30.43(26.21,35.13) | 1.91(1.64,2.19) |
| Lithuania | male | 14.26(7.30,23.80) | 9.89(3.16,19.29) | -0.40(-1.18,0.38) | 4.91(2.70,8.23) | 5.88(2.80,11.09) | 1.09(0.47,1.71) |
| Luxembourg | male | 2.42(0.83,4.90) | 4.25(1.19,8.96) | 2.94(2.29,3.59) | 8.62(1.34,30.46) | 10.63(3.04,27.56) | 1.46(1.04,1.88) |
| Madagascar | male | 659.07(398.09,1018.74) | 1258.55(778.26,1867.76) | 2.23(1.95,2.50) | 59.84(55.34,64.60) | 46.84(44.28,49.50) | -0.83(-1.08,-0.58) |
| Malawi | male | 542.70(329.16,847.27) | 1324.39(864.68,1977.25) | 2.61(2.19,3.04) | 61.53(56.46,66.94) | 71.48(67.65,75.47) | 0.17(-0.29,0.63) |
| Malaysia | male | 652.51(457.53,896.28) | 1275.08(851.23,1868.95) | 1.74(1.35,2.13) | 38.81(35.89,41.91) | 43.05(40.71,45.48) | -0.47(-0.81,-0.13) |
| Maldives | male | 6.46(4.23,9.15) | 14.45(9.72,20.95) | 4.02(3.69,4.36) | 30.62(11.74,65.29) | 32.85(17.89,56.61) | 0.45(0.20,0.70) |
| Mali | male | 260.59(155.52,391.34) | 912.21(557.97,1348.80) | 4.13(3.89,4.38) | 34.63(30.54,39.14) | 40.70(38.09,43.44) | 0.30(0.16,0.44) |
| Malta | male | 4.84(2.16,8.65) | 3.65(1.39,7.13) | -1.20(-1.59,-0.81) | 18.58(5.89,44.15) | 14.12(3.53,40.07) | -1.27(-1.47,-1.08) |
| Marshall Islands | male | 5.34(3.40,7.95) | 9.56(6.12,14.29) | 2.02(1.55,2.49) | 131.68(44.57,301.86) | 186.50(87.66,348.04) | 1.36(1.21,1.51) |
| Mauritania | male | 69.86(40.68,109.58) | 108.56(61.28,177.00) | 1.45(1.35,1.55) | 35.37(27.56,44.79) | 26.64(21.86,32.20) | -1.00(-1.08,-0.92) |
| Mauritius | male | 28.99(18.04,44.88) | 63.09(44.44,85.96) | 3.96(3.57,4.35) | 26.69(17.86,38.45) | 65.89(50.63,84.35) | 4.36(3.90,4.82) |
| Mexico | male | 6470.99(4775.69,8540.57) | 9153.38(6430.23,12901.59) | 1.30(1.08,1.52) | 74.36(72.55,76.20) | 84.37(82.65,86.12) | 0.62(0.40,0.84) |
| Micronesia (Federated States of) | male | 7.66(5.09,11.01) | 19.35(12.05,30.62) | 2.93(2.22,3.64) | 84.71(35.48,171.29) | 187.36(113.35,291.76) | 2.68(2.13,3.24) |
| Monaco | male | 0.15(0.04,0.30) | 0.26(0.10,0.52) | 2.68(2.11,3.26) | 8.34(0.00,295.07) | 14.05(0.00,230.36) | 2.09(1.86,2.32) |
| Mongolia | male | 41.78(23.99,66.71) | 55.53(29.84,96.84) | 1.56(0.99,2.13) | 18.92(13.61,25.63) | 23.09(17.41,30.10) | 0.93(0.67,1.19) |
| Montenegro | male | 8.09(4.26,13.59) | 5.73(2.75,10.52) | -1.15(-1.42,-0.88) | 14.73(6.40,29.07) | 13.19(4.70,29.45) | -0.38(-0.57,-0.19) |
| Morocco | male | 397.60(203.90,646.51) | 764.41(402.46,1314.12) | 2.44(2.23,2.64) | 16.01(14.47,17.67) | 24.78(23.06,26.60) | 1.61(1.54,1.68) |
| Mozambique | male | 532.59(315.31,783.27) | 2457.20(1592.20,3728.88) | 5.56(5.33,5.79) | 53.10(48.64,57.87) | 91.76(88.15,95.47) | 2.37(2.17,2.58) |
| Myanmar | male | 3503.48(1968.87,5763.76) | 4915.37(3293.73,7084.25) | 0.97(0.82,1.13) | 87.44(84.56,90.38) | 103.68(100.80,106.62) | 0.44(0.29,0.59) |
| Namibia | male | 67.17(41.35,94.84) | 140.20(89.95,200.33) | 2.18(1.42,2.95) | 46.85(36.30,59.57) | 60.41(50.82,71.28) | 0.50(-0.09,1.10) |
| Nauru | male | 0.99(0.62,1.56) | 2.01(1.25,3.32) | 2.16(1.79,2.52) | 106.65(2.58,603.50) | 190.30(23.20,689.39) | 1.80(1.55,2.05) |
| Nepal | male | 474.88(280.98,733.97) | 1571.10(947.89,2417.91) | 4.04(3.84,4.24) | 27.74(25.30,30.36) | 52.02(49.48,54.66) | 2.11(1.89,2.33) |
| Netherlands | male | 82.60(27.83,168.24) | 113.16(31.73,245.64) | 2.12(1.24,3.02) | 6.39(5.08,7.94) | 10.32(8.50,12.41) | 2.50(1.93,3.06) |
| New Zealand | male | 6.02(2.70,17.68) | 24.60(5.84,57.50) | 4.89(4.39,5.39) | 2.09(0.77,4.54) | 8.21(5.29,12.17) | 4.47(4.03,4.90) |
| Nicaragua | male | 164.83(110.40,233.79) | 481.75(316.22,688.95) | 3.89(3.65,4.13) | 45.13(38.48,52.63) | 76.20(69.55,83.32) | 1.94(1.85,2.03) |
| Niger | male | 233.88(126.98,388.08) | 793.58(485.30,1219.68) | 3.89(3.54,4.25) | 32.63(28.58,37.13) | 34.66(32.28,37.18) | -0.08(-0.33,0.17) |
| Nigeria | male | 3039.97(1956.77,4266.80) | 7321.24(4712.98,10472.55) | 2.82(2.51,3.13) | 36.34(35.06,37.65) | 34.34(33.55,35.14) | -0.37(-0.60,-0.15) |
| Niue | male | 0.21(0.14,0.30) | 0.25(0.17,0.37) | 0.53(0.35,0.70) | 106.10(0.00,2132.77) | 204.95(0.00,3504.11) | 2.49(2.28,2.70) |
| North Macedonia | male | 17.46(7.87,31.45) | 17.50(8.86,31.11) | 0.23(-0.27,0.74) | 9.86(5.79,15.73) | 11.59(6.80,18.69) | 0.48(0.16,0.81) |
| Northern Mariana Islands | male | 2.72(1.71,4.02) | 3.73(2.31,5.68) | 1.00(0.50,1.51) | 61.18(11.13,199.79) | 98.80(25.22,263.22) | 1.68(1.51,1.85) |
| Norway | male | 70.99(32.81,127.43) | 90.28(40.46,162.68) | -0.63(-1.09,-0.17) | 20.57(16.06,25.99) | 25.48(20.49,31.36) | -0.97(-1.61,-0.32) |
| Oman | male | 26.35(13.13,45.27) | 116.52(63.24,194.70) | 5.76(5.30,6.23) | 13.93(9.12,20.45) | 23.05(18.88,28.09) | 1.67(1.36,1.98) |
| Pakistan | male | 4989.89(2908.24,7524.73) | 19530.20(12156.41,29031.61) | 4.70(4.60,4.81) | 45.54(44.28,46.82) | 84.86(83.67,86.06) | 2.11(2.03,2.18) |
| Palau | male | 1.48(0.96,2.13) | 2.27(1.51,3.26) | 1.46(0.89,2.03) | 89.53(6.24,389.10) | 169.89(24.77,592.27) | 2.17(1.92,2.41) |
| Palestine | male | 45.37(26.25,72.89) | 128.49(72.33,211.10) | 3.76(3.55,3.96) | 21.68(15.83,29.01) | 24.57(20.50,29.20) | 0.23(0.03,0.42) |
| Panama | male | 87.13(56.58,128.80) | 233.28(154.84,339.04) | 3.36(3.26,3.47) | 35.69(28.59,44.03) | 64.25(56.26,73.06) | 2.08(1.91,2.26) |
| Papua New Guinea | male | 612.98(368.88,974.98) | 2060.75(1339.55,3020.53) | 4.18(4.03,4.33) | 148.28(136.77,160.51) | 208.92(200.00,218.14) | 0.94(0.77,1.11) |
| Paraguay | male | 85.99(56.26,128.68) | 274.28(180.46,395.72) | 4.19(3.83,4.55) | 23.45(18.75,28.97) | 41.52(36.75,46.74) | 1.87(1.67,2.06) |
| Peru | male | 370.70(240.81,527.64) | 756.24(478.98,1135.44) | 2.88(2.71,3.04) | 17.16(15.46,19.00) | 25.08(23.32,26.93) | 1.75(1.55,1.95) |
| Philippines | male | 11026.43(8348.34,14886.28) | 7362.75(5434.05,10621.00) | -2.34(-2.88,-1.80) | 174.16(170.93,177.45) | 68.99(67.42,70.59) | -4.15(-4.69,-3.60) |
| Poland | male | 259.37(141.40,445.67) | 268.95(125.27,481.57) | 0.71(0.20,1.23) | 9.94(8.77,11.23) | 12.52(11.07,14.12) | 1.38(0.61,2.15) |
| Portugal | male | 158.65(83.86,259.15) | 121.77(54.28,222.88) | -1.88(-2.26,-1.49) | 19.15(16.28,22.37) | 20.61(17.11,24.62) | -0.23(-0.51,0.04) |
| Puerto Rico | male | 106.00(65.50,164.51) | 158.70(100.59,245.66) | 1.17(0.67,1.67) | 34.41(28.17,41.64) | 67.92(57.76,79.36) | 2.08(1.70,2.46) |
| Qatar | male | 13.45(8.00,21.13) | 125.31(67.89,206.55) | 10.78(9.58,11.99) | 30.13(15.83,53.50) | 30.92(24.87,38.62) | 0.79(0.17,1.42) |
| Republic of Korea | male | 315.68(194.49,487.06) | 516.59(244.50,904.35) | 1.74(1.22,2.27) | 6.72(6.00,7.50) | 14.56(13.33,15.89) | 2.69(2.04,3.35) |
| Republic of Moldova | male | 48.38(28.29,77.58) | 22.66(10.87,41.69) | -2.15(-2.75,-1.55) | 15.50(11.44,20.55) | 10.48(6.62,15.85) | -1.15(-1.34,-0.96) |
| Romania | male | 156.13(91.65,250.05) | 88.39(39.59,156.82) | -2.16(-2.58,-1.74) | 7.87(6.69,9.21) | 8.51(6.83,10.49) | 0.38(0.26,0.51) |
| Russian Federation | male | 1051.09(547.68,1822.92) | 628.10(293.46,1139.83) | -2.10(-2.79,-1.41) | 10.12(9.52,10.75) | 8.45(7.80,9.14) | -1.15(-1.43,-0.87) |
| Rwanda | male | 425.43(249.40,657.01) | 553.14(345.32,822.00) | 0.26(-0.19,0.72) | 65.92(59.79,72.52) | 43.57(40.01,47.36) | -2.71(-3.19,-2.23) |
| Saint Kitts and Nevis | male | 1.85(1.29,2.60) | 3.39(2.31,4.84) | 3.50(3.02,3.98) | 46.16(4.91,175.55) | 76.36(17.88,212.02) | 2.83(2.41,3.26) |
| Saint Lucia | male | 10.49(7.64,14.18) | 15.23(10.59,21.06) | 1.27(1.07,1.47) | 75.17(36.77,136.63) | 107.53(60.45,177.42) | 1.07(0.93,1.20) |
| Saint Vincent and the Grenadines | male | 7.11(5.12,9.58) | 10.14(7.28,13.87) | 0.73(0.45,1.02) | 60.74(24.60,125.17) | 107.61(51.90,197.57) | 1.58(1.28,1.87) |
| Samoa | male | 13.96(9.35,20.18) | 24.36(15.99,34.51) | 1.52(1.36,1.68) | 75.00(40.88,126.44) | 119.12(76.47,177.26) | 1.42(1.24,1.61) |
| San Marino | male | 0.17(0.04,0.36) | 0.29(0.11,0.58) | 1.99(1.53,2.46) | 8.40(0.00,217.27) | 13.89(0.00,210.80) | 1.89(1.64,2.14) |
| Sao Tome and Principe | male | 2.24(1.19,3.55) | 7.91(5.22,11.46) | 3.82(3.44,4.21) | 18.79(2.66,66.45) | 37.63(16.11,75.21) | 2.03(1.75,2.31) |
| Saudi Arabia | male | 493.80(313.93,712.81) | 1638.44(949.53,2600.05) | 5.09(4.68,5.50) | 27.94(25.53,30.52) | 45.73(43.52,48.02) | 2.24(1.93,2.54) |
| Senegal | male | 346.69(218.21,516.39) | 951.70(616.82,1391.95) | 3.49(3.25,3.74) | 48.76(43.74,54.21) | 58.92(55.23,62.80) | 0.77(0.55,1.00) |
| Serbia | male | 70.83(36.36,125.41) | 55.19(24.02,102.86) | -1.32(-1.56,-1.07) | 10.50(8.19,13.24) | 9.84(7.41,12.81) | -0.27(-0.33,-0.21) |
| Seychelles | male | 1.56(0.95,2.45) | 3.70(2.39,5.43) | 3.34(3.21,3.48) | 21.70(1.70,90.62) | 47.46(12.01,129.34) | 2.79(2.67,2.91) |
| Sierra Leone | male | 92.96(49.96,156.52) | 306.38(183.54,472.05) | 4.92(4.54,5.31) | 29.36(23.69,35.99) | 35.42(31.56,39.62) | 0.63(0.40,0.86) |
| Singapore | male | 18.78(7.18,38.61) | 66.10(29.42,117.11) | 4.89(4.41,5.36) | 5.84(3.50,9.21) | 24.42(18.89,31.06) | 4.66(4.29,5.02) |
| Slovakia | male | 26.22(12.85,46.33) | 20.20(5.94,41.81) | -1.02(-1.63,-0.40) | 6.70(4.38,9.80) | 6.48(3.97,10.08) | -0.23(-0.40,-0.06) |
| Slovenia | male | 12.18(6.05,21.92) | 8.70(3.59,16.79) | -1.28(-1.78,-0.78) | 8.00(4.16,14.00) | 8.45(3.80,16.41) | 0.06(-0.14,0.26) |
| Solomon Islands | male | 38.07(23.14,60.26) | 145.60(89.06,232.55) | 4.39(4.08,4.71) | 114.23(80.77,157.06) | 236.00(199.18,277.69) | 2.55(2.33,2.76) |
| Somalia | male | 307.02(189.45,466.64) | 1355.62(820.15,2017.73) | 4.19(3.56,4.82) | 50.87(45.19,57.09) | 64.08(60.71,67.60) | 0.63(0.45,0.81) |
| South Africa | male | 1470.41(1097.40,1916.72) | 3037.05(2356.99,3903.56) | 2.80(1.92,3.70) | 40.14(38.11,42.24) | 63.74(61.49,66.05) | 1.64(1.00,2.29) |
| South Sudan | male | 236.31(141.54,361.42) | 396.18(236.50,606.56) | 1.40(1.03,1.77) | 36.73(32.19,41.74) | 44.21(39.90,48.88) | 0.37(0.21,0.54) |
| Spain | male | 306.96(149.47,544.50) | 404.12(157.58,810.09) | 0.52(0.17,0.87) | 9.13(8.14,10.22) | 17.52(15.85,19.31) | 2.05(1.75,2.36) |
| Sri Lanka | male | 567.69(368.59,822.33) | 987.01(674.14,1399.33) | 1.77(1.40,2.14) | 34.21(31.45,37.14) | 59.81(56.13,63.66) | 1.99(1.67,2.31) |
| Sudan | male | 306.86(164.83,498.83) | 1110.12(595.18,1806.78) | 4.73(4.63,4.83) | 16.56(14.76,18.53) | 26.26(24.74,27.85) | 1.95(1.81,2.09) |
| Suriname | male | 27.40(18.38,38.60) | 48.32(33.98,67.27) | 2.25(1.88,2.61) | 64.66(42.74,94.04) | 103.58(76.46,137.23) | 1.58(1.28,1.87) |
| Sweden | male | 68.22(14.48,155.60) | 96.70(34.40,188.75) | 1.54(0.50,2.60) | 11.12(8.64,14.12) | 16.16(13.10,19.73) | 1.01(0.10,1.93) |
| Switzerland | male | 45.27(12.42,94.90) | 61.67(21.07,129.41) | 1.11(0.44,1.79) | 8.47(6.17,11.41) | 11.80(9.03,15.19) | 0.69(0.12,1.26) |
| Syrian Arab Republic | male | 607.70(418.95,839.40) | 518.18(320.88,790.79) | -1.40(-2.23,-0.56) | 46.80(43.14,50.69) | 36.53(33.41,39.87) | -1.02(-1.32,-0.73) |
| Taiwan (Province of China) | male | 491.88(301.28,755.60) | 387.97(219.11,618.18) | -1.22(-1.54,-0.90) | 25.16(22.98,27.49) | 25.04(22.60,27.69) | -0.39(-0.65,-0.14) |
| Tajikistan | male | 121.41(74.22,184.95) | 343.53(214.33,507.86) | 3.09(2.68,3.50) | 24.00(19.91,28.68) | 37.89(33.99,42.13) | 0.52(0.01,1.02) |
| Thailand | male | 1580.64(1009.17,2292.37) | 1981.35(1361.89,2869.06) | -0.50(-1.00,0.01) | 25.86(24.60,27.17) | 41.09(39.29,42.94) | 0.31(-0.20,0.82) |
| Timor-Leste | male | 30.60(16.02,51.73) | 68.52(43.78,100.99) | 2.40(1.74,3.06) | 39.60(26.83,56.35) | 47.94(37.23,60.82) | 0.24(-0.28,0.75) |
| Togo | male | 120.22(71.58,183.56) | 325.72(202.91,507.62) | 3.18(2.82,3.54) | 32.82(27.20,39.30) | 41.12(36.77,45.85) | 0.49(0.13,0.86) |
| Tokelau | male | 0.09(0.05,0.13) | 0.12(0.08,0.18) | 1.13(0.91,1.35) | 61.76(0.00,2936.97) | 108.67(0.00,3659.90) | 1.99(1.84,2.14) |
| Tonga | male | 5.09(3.17,7.71) | 9.58(6.29,13.59) | 2.34(2.14,2.54) | 51.46(16.83,120.52) | 102.87(48.30,192.49) | 2.66(2.51,2.81) |
| Trinidad and Tobago | male | 49.77(32.38,73.93) | 65.44(44.43,95.25) | 1.37(0.60,2.14) | 46.02(34.13,60.73) | 71.90(55.54,91.60) | 1.87(1.56,2.19) |
| Tunisia | male | 144.60(78.42,238.46) | 252.41(131.77,421.90) | 2.17(1.73,2.62) | 17.13(14.45,20.17) | 30.75(27.07,34.79) | 2.17(2.09,2.25) |
| Turkey | male | 1394.80(823.12,2081.14) | 1391.46(770.10,2308.30) | 0.00(-0.51,0.52) | 23.34(22.13,24.60) | 19.68(18.66,20.75) | -0.42(-0.89,0.06) |
| Turkmenistan | male | 72.52(42.44,110.80) | 146.58(87.37,226.97) | 2.57(2.07,3.08) | 20.29(15.88,25.55) | 33.40(28.20,39.29) | 1.52(1.24,1.80) |
| Tuvalu | male | 0.61(0.40,0.88) | 1.54(1.03,2.21) | 3.81(3.61,4.00) | 78.13(0.25,626.17) | 132.23(10.10,556.96) | 1.97(1.78,2.16) |
| Uganda | male | 786.05(484.75,1216.89) | 2773.95(1839.15,4124.36) | 3.77(3.36,4.19) | 49.26(45.86,52.84) | 67.38(64.88,69.95) | 0.43(0.03,0.83) |
| Ukraine | male | 503.20(274.34,816.35) | 352.55(198.60,581.71) | -1.66(-2.14,-1.16) | 14.09(12.88,15.37) | 15.43(13.86,17.14) | -0.43(-0.86,0.01) |
| United Arab Emirates | male | 29.78(15.05,50.60) | 78.15(36.91,136.13) | 5.09(2.37,7.88) | 16.11(10.75,23.56) | 22.54(17.82,28.12) | 1.32(1.06,1.57) |
| United Kingdom | male | 787.99(381.32,1353.09) | 2633.07(1479.69,4300.94) | 6.36(5.11,7.63) | 18.14(16.90,19.46) | 63.10(60.71,65.57) | 5.91(4.69,7.15) |
| United Republic of Tanzania | male | 1035.08(610.02,1597.54) | 2590.21(1615.53,4001.18) | 2.68(2.48,2.89) | 44.68(41.99,47.51) | 50.05(48.14,52.03) | 0.07(-0.07,0.21) |
| United States of America | male | 1281.31(552.61,3071.57) | 6138.45(2939.97,10549.01) | 6.82(6.21,7.44) | 6.45(6.10,6.81) | 27.57(26.89,28.27) | 6.10(5.58,6.62) |
| United States Virgin Islands | male | 4.11(2.69,5.89) | 4.45(2.87,6.51) | -0.91(-1.65,-0.17) | 49.60(13.71,127.72) | 73.65(21.86,181.32) | 0.06(-0.75,0.88) |
| Uruguay | male | 7.60(4.70,12.50) | 12.17(6.88,21.92) | 1.32(0.87,1.77) | 3.09(1.30,6.21) | 4.70(2.44,8.20) | 1.25(0.82,1.68) |
| Uzbekistan | male | 356.26(192.59,559.28) | 837.99(491.79,1340.66) | 2.88(2.23,3.52) | 18.07(16.24,20.05) | 27.59(25.75,29.53) | 0.95(0.45,1.45) |
| Vanuatu | male | 8.31(5.29,12.38) | 29.30(19.28,42.20) | 4.70(4.56,4.83) | 60.36(26.53,117.91) | 109.47(73.47,157.01) | 2.20(2.14,2.26) |
| Venezuela (Bolivarian Republic of) | male | 752.56(507.83,1053.52) | 1330.43(931.77,1889.03) | 2.20(1.76,2.65) | 40.37(37.54,43.37) | 63.39(60.02,66.89) | 1.24(1.01,1.47) |
| Viet Nam | male | 1734.23(1099.42,2646.39) | 2744.09(1853.23,3966.29) | 2.26(1.95,2.57) | 26.67(25.43,27.95) | 37.50(36.11,38.93) | 1.44(1.15,1.73) |
| Yemen | male | 119.15(49.20,211.50) | 421.52(194.60,758.82) | 3.82(3.03,4.61) | 11.07(9.16,13.26) | 14.62(13.26,16.09) | 0.47(-0.44,1.39) |
| Zambia | male | 426.28(255.99,624.48) | 1379.28(905.97,2007.15) | 3.83(3.52,4.14) | 54.53(49.45,60.00) | 76.10(72.14,80.24) | 0.83(0.37,1.29) |
| Zimbabwe | male | 1153.16(830.38,1539.22) | 3215.74(2178.64,4617.09) | 3.05(2.37,3.72) | 109.22(102.97,115.76) | 215.77(208.35,223.38) | 2.25(1.69,2.82) |

**Supplementary table 4. The incidence cases and age-standardized Incidence rate of type 2 diabetes among adolescents in 1990 and 2019, and its temporal trends from 1990 to 2019.**

|  |  | Incidence No.(95%UI) |  |  | ASIR (per 100000) No.95%UI) |  |  |
| --- | --- | --- | --- | --- | --- | --- | --- |
| nation | sex | 1990 | 2019 | 1990-2019 EAPC No.(95%CI) | 1990 | 2019 | 1990-2019 EAPC No.(95%CI) |
| Afghanistan | both | 2080.02(1531.82,2698.32) | 14282.64(10566.95,18767.57) | 6.22(5.57,6.87) | 91.00(87.10,95.04) | 179.63(176.69,182.60) | 2.41(2.31,2.51) |
| Albania | both | 206.33(133.21,289.21) | 222.17(146.61,310.02) | 0.31(0.03,0.60) | 32.52(28.23,37.27) | 51.71(45.11,59.03) | 1.47(1.28,1.65) |
| Algeria | both | 3358.01(2385.22,4583.55) | 7914.39(5688.30,10794.71) | 3.03(2.41,3.66) | 64.35(62.19,66.57) | 122.92(120.22,125.67) | 2.12(1.87,2.37) |
| American Samoa | both | 32.99(24.96,42.32) | 67.63(51.70,88.72) | 2.17(1.91,2.43) | 337.54(232.31,474.42) | 623.60(483.66,791.91) | 2.07(1.80,2.34) |
| Andorra | both | 3.35(1.95,4.91) | 8.61(5.73,12.08) | 2.71(2.35,3.07) | 38.56(8.83,110.42) | 100.05(44.75,193.72) | 3.32(3.28,3.35) |
| Angola | both | 1482.35(1076.60,1961.96) | 6266.61(4736.26,8109.18) | 5.17(5.06,5.29) | 76.93(73.06,80.96) | 112.53(109.75,115.35) | 1.40(1.29,1.51) |
| Antigua and Barbuda | both | 13.18(9.92,17.12) | 23.89(17.95,30.88) | 2.08(1.99,2.18) | 114.46(61.23,195.60) | 168.47(107.75,251.63) | 1.19(1.14,1.24) |
| Argentina | both | 1734.31(1263.67,2312.06) | 5611.12(4008.27,7489.41) | 4.60(4.39,4.82) | 32.46(30.95,34.03) | 77.49(75.47,79.54) | 3.59(3.33,3.85) |
| Armenia | both | 351.05(247.11,476.79) | 348.11(242.73,471.41) | 0.50(-0.15,1.15) | 61.90(55.59,68.73) | 94.85(85.11,105.43) | 1.44(1.29,1.59) |
| Australia | both | 457.25(233.40,715.89) | 1085.90(548.55,1714.64) | 2.84(2.26,3.42) | 16.77(15.27,18.39) | 34.72(32.68,36.86) | 2.19(1.75,2.64) |
| Austria | both | 380.60(250.67,539.07) | 698.21(446.75,1002.77) | 2.59(2.34,2.83) | 30.78(27.73,34.08) | 68.04(63.06,73.33) | 2.76(2.70,2.82) |
| Azerbaijan | both | 967.45(697.66,1283.90) | 1570.77(1136.71,2100.38) | 2.43(1.98,2.88) | 68.52(64.26,72.98) | 103.70(98.61,109.00) | 1.35(1.22,1.47) |
| Bahamas | both | 55.96(42.16,72.45) | 111.20(84.30,146.97) | 2.57(2.48,2.67) | 103.73(78.35,134.79) | 179.03(147.28,215.66) | 1.87(1.82,1.92) |
| Bahrain | both | 58.46(41.77,80.70) | 247.65(180.13,329.87) | 5.86(5.03,6.68) | 66.19(50.18,86.00) | 150.58(132.41,170.56) | 2.83(2.71,2.95) |
| Bangladesh | both | 14378.35(10550.75,19367.92) | 30097.60(22416.05,40105.19) | 2.10(1.91,2.30) | 66.66(65.58,67.76) | 100.49(99.36,101.63) | 0.99(0.86,1.12) |
| Barbados | both | 52.55(40.17,66.58) | 64.32(48.70,82.11) | 0.70(0.53,0.87) | 114.41(85.56,149.98) | 164.93(127.10,210.50) | 1.10(1.02,1.18) |
| Belarus | both | 686.66(460.53,939.34) | 432.49(265.07,624.54) | -1.49(-2.20,-0.78) | 46.92(43.48,50.57) | 46.14(41.89,50.71) | -0.41(-0.63,-0.18) |
| Belgium | both | 603.41(393.87,856.77) | 1283.47(869.80,1800.95) | 2.82(2.61,3.02) | 42.23(38.92,45.76) | 98.13(92.82,103.66) | 2.82(2.74,2.91) |
| Belize | both | 30.66(23.46,38.43) | 120.65(93.08,152.85) | 4.86(4.61,5.10) | 81.39(55.13,116.01) | 143.58(119.10,171.66) | 1.89(1.66,2.13) |
| Benin | both | 294.10(197.32,408.47) | 1718.43(1220.90,2289.53) | 6.57(6.06,7.07) | 35.41(31.48,39.71) | 69.30(66.05,72.66) | 2.60(2.12,3.09) |
| Bermuda | both | 6.10(4.58,7.99) | 6.55(4.87,8.52) | 0.37(0.25,0.50) | 68.30(25.13,151.34) | 106.28(41.13,224.90) | 1.42(1.34,1.50) |
| Bhutan | both | 91.62(65.81,123.36) | 151.91(108.77,203.24) | 2.56(2.30,2.83) | 66.65(53.70,81.80) | 101.64(86.08,119.27) | 1.53(1.47,1.59) |
| Bolivia (Plurinational State of) | both | 527.25(376.16,708.60) | 1393.01(995.64,1882.76) | 3.44(3.22,3.67) | 44.00(40.32,47.93) | 64.89(61.53,68.40) | 1.24(1.18,1.30) |
| Bosnia and Herzegovina | both | 376.85(255.48,525.58) | 342.17(228.78,481.60) | 0.35(-0.21,0.92) | 46.44(41.86,51.38) | 80.85(72.49,89.96) | 2.02(1.88,2.16) |
| Botswana | both | 143.29(101.02,196.33) | 343.71(248.38,462.73) | 2.92(2.54,3.29) | 53.74(45.27,63.36) | 81.47(73.08,90.56) | 1.39(1.30,1.47) |
| Brazil | both | 19384.56(13814.34,26606.68) | 29622.42(20780.80,40623.90) | 1.58(1.48,1.67) | 66.47(65.54,67.41) | 86.20(85.22,87.19) | 1.02(0.88,1.16) |
| Brunei Darussalam | both | 83.26(63.03,108.87) | 124.65(90.57,166.12) | 1.24(1.11,1.36) | 163.16(129.96,202.42) | 161.81(134.60,193.05) | -0.38(-0.59,-0.18) |
| Bulgaria | both | 675.87(480.11,925.70) | 484.28(337.80,661.95) | -1.94(-2.29,-1.58) | 56.37(52.20,60.79) | 77.54(70.79,84.77) | 0.35(0.08,0.63) |
| Burkina Faso | both | 661.27(444.86,915.59) | 2796.18(1985.80,3777.52) | 5.03(4.98,5.09) | 40.29(37.27,43.50) | 65.63(63.21,68.12) | 1.61(1.57,1.65) |
| Burundi | both | 616.51(439.94,810.36) | 1562.16(1139.02,2081.09) | 3.52(3.35,3.69) | 62.07(57.26,67.18) | 68.67(65.30,72.17) | 0.08(-0.02,0.17) |
| Cabo Verde | both | 28.01(18.37,39.43) | 79.07(55.11,108.80) | 4.22(3.58,4.87) | 40.33(26.79,58.35) | 77.37(61.25,96.49) | 2.33(2.09,2.57) |
| Cambodia | both | 917.86(706.86,1173.22) | 2736.79(2030.43,3578.08) | 4.16(3.99,4.33) | 47.97(44.92,51.18) | 92.59(89.15,96.13) | 2.15(1.96,2.35) |
| Cameroon | both | 602.26(394.28,837.32) | 3572.74(2518.64,4792.19) | 7.21(5.92,8.52) | 31.45(28.99,34.08) | 60.75(58.77,62.78) | 3.10(1.78,4.44) |
| Canada | both | 225.76(69.82,432.81) | 931.54(305.94,1799.57) | 4.10(3.51,4.69) | 5.78(5.05,6.59) | 22.00(20.60,23.46) | 3.52(2.82,4.22) |
| Central African Republic | both | 443.59(315.80,583.03) | 1464.44(1084.99,1916.93) | 4.14(3.87,4.40) | 84.50(76.82,92.75) | 135.65(128.79,142.79) | 1.64(1.52,1.76) |
| Chad | both | 381.47(255.57,533.70) | 1949.99(1374.78,2597.34) | 5.70(5.65,5.75) | 35.81(32.30,39.61) | 63.27(60.47,66.16) | 1.97(1.86,2.08) |
| Chile | both | 945.73(653.25,1297.04) | 1957.25(1373.72,2712.42) | 2.84(2.66,3.01) | 36.94(34.62,39.37) | 73.10(69.89,76.42) | 2.30(2.20,2.39) |
| China | both | 246041.87(177628.34,331383.51) | 223155.50(160950.90,301313.90) | 2.63(1.65,3.63) | 93.42(93.05,93.79) | 138.51(137.93,139.08) | 3.64(2.96,4.32) |
| Colombia | both | 6035.47(4544.30,8022.04) | 12245.44(9249.62,15985.69) | 2.27(2.11,2.42) | 91.14(88.85,93.47) | 149.95(147.30,152.63) | 1.45(1.32,1.58) |
| Comoros | both | 47.36(33.91,63.00) | 86.17(62.55,115.17) | 1.89(1.82,1.97) | 51.50(37.84,68.60) | 62.66(50.13,77.39) | 0.55(0.50,0.61) |
| Congo | both | 453.16(326.85,598.82) | 1082.83(812.64,1418.11) | 2.68(2.47,2.88) | 91.97(83.68,100.87) | 115.16(108.40,122.24) | 0.61(0.53,0.70) |
| Cook Islands | both | 6.48(5.07,8.25) | 9.19(7.07,11.73) | 1.02(0.82,1.21) | 176.20(67.65,375.52) | 343.79(158.70,648.72) | 2.32(2.27,2.37) |
| Costa Rica | both | 457.10(338.03,595.25) | 1248.75(952.73,1635.90) | 3.63(3.31,3.96) | 78.28(71.27,85.80) | 162.83(153.90,172.16) | 2.43(2.38,2.48) |
| Croatia | both | 351.24(245.87,483.27) | 374.15(249.74,532.82) | -0.16(-0.35,0.02) | 50.40(45.27,55.97) | 76.54(68.95,84.78) | 1.11(0.99,1.22) |
| Cuba | both | 2794.48(2050.56,3628.58) | 2357.42(1717.56,3108.23) | -0.64(-1.05,-0.23) | 120.88(116.44,125.45) | 167.43(160.73,174.34) | 0.68(0.45,0.92) |
| Cyprus | both | 51.91(34.40,72.22) | 139.75(89.11,203.91) | 3.94(3.48,4.40) | 41.02(30.62,53.89) | 90.02(75.57,106.57) | 2.67(2.56,2.78) |
| Czechia | both | 914.20(638.84,1270.54) | 1040.88(691.47,1487.40) | -0.04(-0.45,0.37) | 62.58(58.58,66.79) | 108.36(101.87,115.15) | 1.70(1.63,1.78) |
| Côte d'Ivoire | both | 863.68(576.54,1210.90) | 3318.91(2393.08,4394.05) | 4.37(3.90,4.83) | 36.99(34.56,39.54) | 66.98(64.72,69.30) | 1.92(1.73,2.10) |
| Democratic People's Republic of Korea | both | 3659.03(2832.39,4728.20) | 5581.45(4301.91,7250.28) | 1.89(1.68,2.10) | 96.33(93.23,99.51) | 141.93(138.22,145.73) | 1.21(1.09,1.32) |
| Democratic Republic of the Congo | both | 4863.46(3464.90,6453.34) | 18904.53(13751.37,24993.55) | 4.78(4.61,4.95) | 67.21(65.33,69.12) | 108.01(106.48,109.57) | 1.52(1.35,1.69) |
| Denmark | both | 322.77(208.00,463.14) | 691.80(478.74,944.76) | 2.98(2.56,3.39) | 41.22(36.84,46.00) | 98.11(90.91,105.73) | 3.10(3.03,3.17) |
| Djibouti | both | 53.15(37.61,71.93) | 152.61(110.60,202.25) | 4.21(3.94,4.48) | 49.85(37.36,65.18) | 74.04(62.76,86.78) | 1.38(1.35,1.41) |
| Dominica | both | 18.18(13.87,23.56) | 24.89(18.87,31.54) | 1.22(1.05,1.39) | 124.97(74.24,197.49) | 226.12(146.17,334.24) | 1.85(1.60,2.11) |
| Dominican Republic | both | 904.58(692.44,1159.15) | 2172.89(1676.60,2769.63) | 3.43(3.28,3.59) | 58.60(54.84,62.55) | 110.67(106.07,115.43) | 2.42(2.30,2.53) |
| Ecuador | both | 833.48(575.83,1182.40) | 2577.32(1854.84,3463.00) | 3.18(2.83,3.54) | 41.34(38.58,44.25) | 78.89(75.87,81.99) | 1.55(1.23,1.88) |
| Egypt | both | 4108.30(2778.44,5622.87) | 18894.21(13596.61,25057.63) | 5.78(5.61,5.95) | 39.27(38.08,40.49) | 104.91(103.42,106.42) | 3.67(3.42,3.91) |
| El Salvador | both | 936.10(713.23,1184.53) | 1930.23(1471.01,2489.93) | 2.72(2.57,2.86) | 86.71(81.23,92.47) | 158.99(151.97,166.26) | 2.25(2.08,2.42) |
| Equatorial Guinea | both | 60.66(43.88,80.30) | 382.02(283.05,493.90) | 6.72(6.67,6.78) | 79.59(60.79,102.46) | 113.51(102.40,125.51) | 1.15(1.04,1.26) |
| Eritrea | both | 358.45(252.50,475.87) | 1152.07(839.84,1534.69) | 4.12(3.89,4.34) | 60.78(54.64,67.44) | 81.11(76.49,85.94) | 0.91(0.82,1.01) |
| Estonia | both | 106.08(71.02,147.32) | 83.05(54.53,118.94) | -0.46(-1.05,0.13) | 49.14(40.24,59.44) | 64.33(51.23,79.82) | 0.90(0.65,1.15) |
| Eswatini | both | 84.04(58.66,113.97) | 204.33(147.69,267.89) | 3.13(2.34,3.92) | 52.09(41.51,64.60) | 87.11(75.57,99.91) | 1.89(1.39,2.40) |
| Ethiopia | both | 6519.77(4749.02,8498.65) | 12395.84(8791.39,16710.83) | 1.90(1.77,2.03) | 68.48(66.82,70.16) | 53.97(53.02,54.93) | -1.04(-1.11,-0.96) |
| Fiji | both | 298.72(229.48,378.23) | 553.51(433.64,697.06) | 2.00(1.68,2.33) | 202.02(179.76,226.29) | 365.15(335.36,396.89) | 1.90(1.76,2.04) |
| Finland | both | 457.89(280.64,682.74) | 1052.64(747.92,1419.08) | 2.96(2.68,3.24) | 68.26(62.13,74.86) | 170.28(160.14,180.92) | 2.95(2.62,3.29) |
| France | both | 1971.51(1296.73,2771.61) | 4355.39(2672.49,6417.08) | 2.12(1.11,3.14) | 22.26(21.29,23.27) | 54.96(53.34,56.62) | 2.45(1.45,3.45) |
| Gabon | both | 151.59(110.92,202.72) | 410.55(308.60,543.47) | 3.52(3.38,3.66) | 81.03(68.64,95.04) | 119.09(107.85,131.19) | 1.29(1.20,1.37) |
| Gambia | both | 56.91(37.46,80.23) | 274.06(191.17,366.03) | 5.39(5.28,5.51) | 29.91(22.65,38.79) | 57.85(51.19,65.14) | 2.33(2.26,2.39) |
| Georgia | both | 615.37(440.13,829.25) | 511.98(368.64,693.35) | -0.11(-0.57,0.34) | 73.19(67.52,79.22) | 122.60(112.19,133.73) | 2.14(1.91,2.36) |
| Germany | both | 7326.88(5135.16,10011.76) | 13490.95(9605.68,18472.09) | 1.91(1.72,2.10) | 65.38(63.86,66.93) | 146.33(143.86,148.84) | 2.26(2.05,2.46) |
| Ghana | both | 1111.16(742.25,1573.21) | 4629.27(3252.96,6230.54) | 4.93(4.60,5.26) | 39.19(36.92,41.57) | 72.77(70.69,74.90) | 1.89(1.54,2.24) |
| Greece | both | 667.38(446.54,928.89) | 1163.83(778.27,1625.77) | 1.32(0.98,1.66) | 42.05(38.92,45.37) | 113.22(106.80,119.92) | 3.24(3.12,3.35) |
| Greenland | both | 0.74(0.20,1.71) | 2.04(0.74,3.58) | 5.28(4.54,6.01) | 7.18(0.04,63.63) | 26.45(3.26,98.02) | 4.98(4.65,5.30) |
| Grenada | both | 21.35(16.48,27.34) | 34.45(25.67,44.36) | 1.47(1.14,1.81) | 133.50(82.94,203.89) | 186.22(129.14,260.64) | 1.08(1.01,1.15) |
| Guam | both | 47.87(37.83,60.88) | 83.53(65.15,106.54) | 2.24(1.96,2.52) | 171.80(126.37,228.90) | 301.64(240.35,374.14) | 2.02(1.90,2.14) |
| Guatemala | both | 1276.69(964.29,1617.90) | 6824.20(5106.31,8638.74) | 6.27(5.90,6.64) | 87.76(83.00,92.72) | 181.11(176.84,185.46) | 2.61(2.38,2.84) |
| Guinea | both | 301.72(196.10,419.54) | 1396.05(1007.26,1862.94) | 5.25(5.07,5.43) | 28.93(25.76,32.40) | 58.22(55.20,61.37) | 2.33(2.17,2.49) |
| Guinea-Bissau | both | 82.47(58.05,113.95) | 290.66(208.68,391.46) | 4.47(4.25,4.68) | 43.29(34.43,53.78) | 75.22(66.82,84.40) | 1.87(1.77,1.97) |
| Guyana | both | 246.14(184.03,312.96) | 357.27(264.33,456.42) | 1.04(0.78,1.30) | 147.74(129.86,167.41) | 228.11(205.00,253.18) | 1.28(1.15,1.42) |
| Haiti | both | 1814.98(1397.32,2291.41) | 4691.49(3569.36,6043.41) | 3.26(3.00,3.53) | 156.44(149.31,163.82) | 201.35(195.63,207.20) | 0.73(0.68,0.78) |
| Honduras | both | 1016.55(761.94,1325.72) | 4032.16(3055.69,5290.47) | 4.82(4.63,5.00) | 116.54(109.45,123.99) | 195.82(189.82,201.96) | 1.74(1.67,1.80) |
| Hungary | both | 875.29(613.82,1222.99) | 826.95(558.14,1168.77) | -0.58(-0.86,-0.30) | 61.20(57.21,65.40) | 79.79(74.43,85.44) | 0.87(0.81,0.94) |
| Iceland | both | 17.23(10.68,25.00) | 51.03(35.79,69.69) | 4.15(4.06,4.25) | 40.30(23.56,64.49) | 109.92(81.77,144.93) | 3.56(3.52,3.60) |
| India | both | 121024.82(84515.85,165020.65) | 339632.61(241401.49,466887.03) | 3.22(2.99,3.46) | 75.36(74.94,75.78) | 128.95(128.51,129.38) | 1.32(1.04,1.60) |
| Indonesia | both | 19488.09(14559.41,25361.47) | 32444.33(24080.50,42716.26) | 0.60(-0.47,1.67) | 52.71(51.98,53.46) | 72.88(72.09,73.68) | 0.18(-0.85,1.22) |
| Iran (Islamic Republic of) | both | 5991.71(3965.75,8450.65) | 10660.23(7137.76,15044.64) | 2.52(1.47,3.59) | 51.98(50.67,53.31) | 92.62(90.86,94.40) | 2.08(1.70,2.47) |
| Iraq | both | 3973.97(2978.47,5139.27) | 14820.79(11044.21,19395.81) | 4.38(4.06,4.70) | 120.03(116.31,123.84) | 168.22(165.53,170.96) | 1.14(0.90,1.38) |
| Ireland | both | 183.67(101.06,274.01) | 506.26(325.42,707.24) | 3.33(3.16,3.50) | 30.22(25.99,34.95) | 83.65(76.52,91.26) | 3.57(3.49,3.65) |
| Israel | both | 337.39(226.58,473.62) | 1323.84(946.46,1756.47) | 3.39(2.33,4.47) | 39.61(35.48,44.08) | 95.80(90.71,101.10) | 1.96(0.85,3.07) |
| Italy | both | 4394.92(2308.70,6866.32) | 4939.39(2849.41,7435.17) | -1.11(-2.88,0.69) | 48.18(46.76,49.63) | 83.57(81.25,85.94) | 0.36(-1.28,2.02) |
| Jamaica | both | 437.73(327.25,571.84) | 832.57(619.73,1095.75) | 2.32(1.91,2.73) | 89.67(81.46,98.48) | 159.77(149.08,171.06) | 1.80(1.41,2.18) |
| Japan | both | 8301.15(5747.64,11445.57) | 9082.46(6338.57,12605.61) | -0.45(-0.77,-0.13) | 43.36(42.43,44.31) | 73.89(72.38,75.43) | 1.55(1.37,1.73) |
| Jordan | both | 578.35(424.43,765.42) | 3049.91(2227.15,4105.57) | 6.06(5.72,6.40) | 67.36(61.97,73.09) | 134.31(129.59,139.17) | 2.54(2.32,2.76) |
| Kazakhstan | both | 2324.49(1690.97,3141.41) | 2982.49(2130.39,4025.25) | 2.13(1.56,2.70) | 84.27(80.88,87.77) | 119.80(115.53,124.18) | 1.89(1.59,2.20) |
| Kenya | both | 1833.22(1222.85,2548.47) | 6528.97(4584.60,8821.40) | 4.31(4.07,4.55) | 39.22(37.44,41.06) | 60.85(59.38,62.35) | 1.51(1.39,1.63) |
| Kiribati | both | 38.86(30.30,48.12) | 97.58(75.27,121.90) | 3.84(3.52,4.17) | 264.60(187.97,362.55) | 449.06(364.39,547.54) | 1.83(1.64,2.02) |
| Kuwait | both | 318.98(223.46,438.00) | 1112.16(808.89,1506.67) | 4.64(4.39,4.89) | 96.51(86.09,107.94) | 196.17(184.71,208.20) | 1.79(1.52,2.05) |
| Kyrgyzstan | both | 438.94(311.25,586.78) | 920.11(648.43,1247.86) | 2.94(2.66,3.21) | 54.76(49.75,60.14) | 81.98(76.76,87.47) | 1.34(1.30,1.38) |
| Lao People's Democratic Republic | both | 598.64(455.57,758.71) | 1537.16(1152.90,1990.54) | 3.29(3.04,3.55) | 79.27(73.03,85.91) | 109.73(104.32,115.36) | 0.92(0.85,0.99) |
| Latvia | both | 189.39(124.58,259.79) | 102.43(65.69,144.63) | -1.50(-2.16,-0.83) | 50.70(43.73,58.47) | 58.13(47.42,70.55) | 0.48(0.30,0.66) |
| Lebanon | both | 406.19(285.40,560.91) | 918.62(662.04,1255.24) | 3.60(3.25,3.95) | 69.77(63.14,76.91) | 129.62(121.36,138.30) | 2.25(2.16,2.34) |
| Lesotho | both | 153.71(104.22,209.97) | 358.42(258.49,477.12) | 3.01(2.70,3.32) | 43.57(36.93,51.08) | 83.72(75.27,92.85) | 2.52(2.39,2.65) |
| Liberia | both | 145.74(102.14,201.89) | 784.88(561.09,1060.41) | 5.63(5.20,6.07) | 47.57(39.99,56.24) | 80.92(75.34,86.79) | 1.80(1.75,1.84) |
| Libya | both | 645.65(458.74,881.13) | 1759.13(1235.42,2386.19) | 3.49(3.06,3.92) | 77.21(71.35,83.42) | 148.09(141.25,155.18) | 2.32(2.24,2.40) |
| Lithuania | both | 194.15(125.40,273.65) | 158.55(98.28,231.41) | -0.13(-0.63,0.36) | 34.13(29.50,39.30) | 48.59(41.29,56.87) | 1.45(1.09,1.80) |
| Luxembourg | both | 21.30(14.07,30.09) | 73.84(49.06,102.76) | 5.01(4.69,5.33) | 40.49(24.98,62.59) | 99.89(78.33,125.76) | 3.50(3.30,3.69) |
| Madagascar | both | 1244.63(887.82,1644.01) | 3825.37(2810.49,5137.13) | 3.80(3.71,3.89) | 54.45(51.46,57.57) | 70.26(68.05,72.53) | 0.77(0.71,0.83) |
| Malawi | both | 1159.58(831.88,1559.17) | 3596.70(2648.64,4813.58) | 3.56(3.37,3.76) | 62.62(59.06,66.33) | 90.82(87.86,93.86) | 1.03(0.89,1.17) |
| Malaysia | both | 2384.61(1768.21,3157.74) | 6813.70(5071.61,9019.13) | 3.69(3.42,3.96) | 70.86(68.05,73.77) | 119.47(116.65,122.35) | 1.60(1.49,1.70) |
| Maldives | both | 21.12(15.49,27.77) | 63.72(47.18,84.74) | 4.59(4.27,4.91) | 49.16(30.46,75.19) | 86.73(66.51,111.56) | 1.92(1.77,2.07) |
| Mali | both | 529.14(352.70,736.07) | 2594.03(1861.37,3484.09) | 5.59(5.50,5.68) | 35.85(32.85,39.05) | 59.49(57.22,61.84) | 1.65(1.60,1.69) |
| Malta | both | 33.04(21.47,47.59) | 58.39(40.06,80.30) | 1.96(1.49,2.44) | 64.26(44.23,90.35) | 124.56(94.47,161.62) | 2.21(2.04,2.38) |
| Marshall Islands | both | 28.81(22.00,37.18) | 66.20(50.08,86.07) | 2.61(2.03,3.18) | 343.42(229.25,495.62) | 651.16(503.74,828.54) | 2.20(1.99,2.41) |
| Mauritania | both | 95.89(60.13,137.53) | 320.70(216.72,444.89) | 4.04(3.90,4.18) | 25.04(20.28,30.61) | 39.55(35.33,44.14) | 1.43(1.31,1.55) |
| Mauritius | both | 190.43(143.19,250.17) | 320.44(238.82,413.42) | 2.91(2.54,3.29) | 89.75(77.44,103.50) | 168.86(150.87,188.43) | 3.30(2.85,3.76) |
| Mexico | both | 24547.83(18502.58,31919.94) | 42513.42(31874.24,54889.19) | 2.16(1.85,2.48) | 135.38(133.69,137.09) | 195.87(194.01,197.74) | 1.61(1.31,1.92) |
| Micronesia (Federated States of) | both | 32.98(25.61,41.33) | 73.44(57.65,92.18) | 2.56(1.73,3.38) | 172.16(118.07,243.24) | 360.35(282.60,453.08) | 2.58(1.87,3.29) |
| Monaco | both | 1.29(0.75,1.92) | 3.80(2.52,5.30) | 4.33(4.00,4.66) | 39.36(1.86,209.93) | 106.17(27.64,278.52) | 3.51(3.47,3.55) |
| Mongolia | both | 159.35(110.76,217.10) | 269.87(183.98,365.10) | 2.36(1.85,2.87) | 36.59(31.13,42.75) | 56.66(50.08,63.89) | 1.66(1.54,1.78) |
| Montenegro | both | 62.81(43.89,87.38) | 72.54(49.22,100.72) | 0.28(0.05,0.50) | 59.87(45.98,76.65) | 90.39(70.78,113.76) | 1.27(1.17,1.37) |
| Morocco | both | 2878.27(2027.73,4012.80) | 7555.99(5397.87,10258.89) | 3.50(3.27,3.73) | 56.98(54.91,59.10) | 123.68(120.91,126.50) | 2.79(2.74,2.84) |
| Mozambique | both | 1229.04(881.59,1624.61) | 5447.13(4074.30,7117.38) | 5.07(4.80,5.34) | 53.76(50.77,56.88) | 92.67(90.22,95.17) | 2.02(1.83,2.21) |
| Myanmar | both | 6032.21(4468.24,7755.60) | 9796.49(7404.24,12696.80) | 1.49(1.43,1.55) | 74.75(72.87,76.66) | 101.96(99.95,104.00) | 0.90(0.83,0.96) |
| Namibia | both | 146.98(102.98,202.44) | 339.04(240.59,457.45) | 2.79(2.54,3.04) | 50.21(42.41,59.05) | 72.13(64.65,80.23) | 1.12(1.03,1.22) |
| Nauru | both | 3.29(2.56,4.09) | 7.07(5.58,8.84) | 2.53(2.42,2.64) | 177.04(40.20,497.66) | 337.36(136.30,695.27) | 2.22(2.18,2.26) |
| Nepal | both | 2283.20(1632.61,3110.52) | 7828.35(5711.08,10452.14) | 3.93(3.63,4.23) | 64.10(61.49,66.79) | 123.45(120.73,126.21) | 1.89(1.57,2.22) |
| Netherlands | both | 872.47(554.59,1255.97) | 1735.27(1127.01,2446.05) | 3.17(2.54,3.80) | 35.79(33.44,38.26) | 82.24(78.42,86.21) | 3.42(3.07,3.77) |
| New Zealand | both | 106.25(37.20,192.24) | 260.36(135.39,413.05) | 2.37(1.51,3.24) | 18.46(15.11,22.32) | 44.37(39.14,50.11) | 1.98(1.15,2.82) |
| Nicaragua | both | 751.51(566.90,984.15) | 2090.63(1594.98,2726.14) | 3.57(3.31,3.82) | 98.18(91.27,105.49) | 167.85(160.73,175.20) | 1.85(1.83,1.88) |
| Niger | both | 242.54(143.44,359.45) | 1776.57(1207.21,2437.61) | 6.77(6.27,7.26) | 17.28(15.17,19.61) | 40.07(38.22,41.99) | 2.79(2.44,3.15) |
| Nigeria | both | 5090.72(3238.94,7306.76) | 16173.81(10413.80,22883.81) | 4.05(3.91,4.20) | 29.63(28.82,30.46) | 37.51(36.93,38.09) | 0.92(0.77,1.07) |
| Niue | both | 0.98(0.75,1.25) | 1.30(1.00,1.65) | 1.02(0.76,1.28) | 257.20(5.98,1500.02) | 531.10(27.78,2528.02) | 2.99(2.84,3.14) |
| North Macedonia | both | 171.50(113.85,243.01) | 218.03(145.94,312.85) | 0.98(0.68,1.28) | 49.97(42.77,58.05) | 77.18(67.23,88.26) | 1.38(1.30,1.47) |
| Northern Mariana Islands | both | 16.71(12.98,21.48) | 24.12(18.77,31.34) | 1.06(0.32,1.80) | 179.97(103.79,293.04) | 320.73(205.44,478.44) | 2.00(1.91,2.08) |
| Norway | both | 474.19(291.83,681.87) | 716.83(470.92,1005.14) | 0.04(-0.35,0.43) | 71.56(65.25,78.33) | 106.75(99.07,114.89) | -0.31(-0.79,0.18) |
| Oman | both | 179.58(127.96,246.00) | 815.81(577.77,1111.37) | 5.12(4.67,5.56) | 56.81(48.81,65.76) | 110.24(102.58,118.38) | 1.87(1.37,2.38) |
| Pakistan | both | 18181.55(13098.98,24656.79) | 61820.47(44734.54,82402.86) | 3.95(3.62,4.28) | 85.78(84.54,87.04) | 136.15(135.08,137.23) | 1.32(1.04,1.60) |
| Palau | both | 6.31(4.85,8.08) | 9.42(7.12,11.88) | 1.44(1.04,1.84) | 201.83(76.42,431.36) | 402.73(188.05,755.66) | 2.45(2.36,2.53) |
| Palestine | both | 251.92(176.09,343.77) | 1104.96(791.70,1505.04) | 5.44(5.33,5.54) | 62.12(54.68,70.31) | 108.29(102.00,114.87) | 1.79(1.68,1.90) |
| Panama | both | 400.50(303.36,520.52) | 1098.12(836.75,1429.00) | 3.47(3.39,3.56) | 82.27(74.41,90.74) | 155.33(146.27,164.80) | 2.24(2.22,2.26) |
| Papua New Guinea | both | 1836.48(1444.82,2297.06) | 7101.33(5538.20,8815.17) | 4.87(4.75,4.99) | 226.82(216.56,237.44) | 376.36(367.66,385.22) | 1.68(1.55,1.82) |
| Paraguay | both | 378.62(270.08,508.89) | 918.25(669.28,1228.72) | 3.09(2.83,3.35) | 50.98(45.97,56.39) | 71.00(66.48,75.75) | 0.94(0.74,1.14) |
| Peru | both | 1228.13(848.72,1703.46) | 3176.78(2270.59,4330.88) | 3.18(3.03,3.34) | 27.92(26.38,29.53) | 55.05(53.15,57.00) | 2.23(2.07,2.39) |
| Philippines | both | 8442.58(6382.24,10988.63) | 14805.71(11060.69,19557.33) | 1.42(1.27,1.58) | 66.24(64.83,67.67) | 70.72(69.59,71.87) | -0.36(-0.54,-0.17) |
| Poland | both | 2804.96(1873.02,4005.54) | 3414.44(2242.28,4858.57) | 0.93(0.59,1.27) | 54.14(52.16,56.19) | 83.41(80.63,86.27) | 1.82(1.23,2.40) |
| Portugal | both | 1016.31(698.30,1406.48) | 1556.46(1069.69,2175.08) | 0.72(0.42,1.03) | 61.65(57.92,65.57) | 135.26(128.62,142.16) | 2.45(2.29,2.60) |
| Puerto Rico | both | 691.18(499.16,943.29) | 917.67(673.57,1228.18) | 1.01(0.79,1.24) | 110.37(102.29,118.93) | 197.97(185.37,211.22) | 2.02(1.92,2.12) |
| Qatar | both | 55.50(38.19,76.39) | 691.95(459.31,973.88) | 11.63(10.52,12.75) | 81.13(60.99,106.21) | 145.06(133.29,157.83) | 2.68(2.35,3.01) |
| Republic of Korea | both | 4635.71(3355.67,6341.38) | 5688.01(4125.72,7641.00) | 1.16(0.69,1.63) | 50.84(49.38,52.32) | 90.66(88.30,93.08) | 2.41(1.82,3.00) |
| Republic of Moldova | both | 371.89(253.80,506.32) | 283.37(180.82,409.78) | -0.43(-1.05,0.20) | 59.11(53.25,65.44) | 67.07(59.48,75.39) | 0.65(0.48,0.82) |
| Romania | both | 1779.05(1233.45,2440.47) | 1214.10(789.03,1753.48) | -1.97(-2.24,-1.69) | 45.52(43.43,47.69) | 60.12(56.78,63.60) | 0.66(0.53,0.78) |
| Russian Federation | both | 8670.80(5735.59,12015.13) | 6550.23(4037.20,9568.94) | -1.12(-1.84,-0.39) | 42.43(41.54,43.33) | 45.05(43.97,46.16) | -0.02(-0.17,0.12) |
| Rwanda | both | 838.95(610.24,1121.15) | 1585.25(1144.86,2148.59) | 2.13(1.61,2.65) | 62.66(58.48,67.06) | 60.81(57.85,63.89) | -0.74(-0.99,-0.48) |
| Saint Kitts and Nevis | both | 9.14(6.82,11.99) | 14.00(10.45,18.53) | 1.52(1.46,1.57) | 114.15(52.55,216.27) | 156.78(85.68,263.69) | 0.87(0.75,1.00) |
| Saint Lucia | both | 47.64(35.90,60.86) | 64.62(48.49,84.66) | 1.09(1.00,1.18) | 167.07(123.01,221.90) | 232.57(179.30,296.98) | 1.06(1.00,1.11) |
| Saint Vincent and the Grenadines | both | 33.57(25.74,42.33) | 39.25(30.01,49.98) | 0.33(0.24,0.42) | 145.44(100.42,203.98) | 215.58(153.48,294.58) | 1.24(1.20,1.29) |
| Samoa | both | 64.97(50.01,82.91) | 135.98(105.20,175.02) | 2.32(2.14,2.51) | 184.59(142.28,235.79) | 333.31(279.41,394.78) | 1.98(1.87,2.10) |
| San Marino | both | 1.60(0.96,2.39) | 4.40(2.93,6.05) | 3.56(3.37,3.74) | 40.30(3.30,170.33) | 105.03(30.95,260.50) | 3.41(3.38,3.44) |
| Sao Tome and Principe | both | 9.02(5.90,12.87) | 27.79(19.29,38.40) | 3.76(3.59,3.92) | 39.01(17.78,74.83) | 67.41(44.67,97.85) | 1.88(1.82,1.93) |
| Saudi Arabia | both | 2500.82(1774.95,3292.64) | 11187.62(8078.83,15277.06) | 6.18(5.76,6.61) | 79.13(76.06,82.30) | 180.05(176.71,183.45) | 3.40(3.12,3.69) |
| Senegal | both | 767.29(525.39,1076.49) | 2687.76(1916.96,3581.73) | 4.42(3.92,4.92) | 53.85(50.09,57.81) | 87.68(84.40,91.07) | 1.82(1.40,2.25) |
| Serbia | both | 714.87(482.56,1005.55) | 799.90(529.50,1161.59) | -0.18(-0.42,0.05) | 53.77(49.90,57.87) | 74.24(69.18,79.57) | 0.97(0.92,1.01) |
| Seychelles | both | 9.44(6.89,12.59) | 21.08(15.35,28.52) | 2.99(2.88,3.10) | 64.95(30.36,121.52) | 144.85(89.69,222.11) | 2.84(2.80,2.88) |
| Sierra Leone | both | 121.41(70.68,176.92) | 696.27(491.34,930.51) | 7.14(6.78,7.50) | 18.60(15.43,22.22) | 40.16(37.23,43.26) | 2.89(2.78,3.01) |
| Singapore | both | 399.32(282.33,546.81) | 653.43(462.89,891.69) | 2.43(1.94,2.92) | 70.61(63.81,77.94) | 120.40(111.32,130.04) | 1.58(1.38,1.79) |
| Slovakia | both | 326.53(221.27,462.86) | 368.94(239.56,535.80) | 0.26(-0.19,0.71) | 41.76(37.35,46.55) | 62.37(56.15,69.12) | 1.31(1.26,1.35) |
| Slovenia | both | 127.39(87.59,178.86) | 131.40(86.76,190.81) | 0.02(-0.30,0.34) | 42.57(35.49,50.67) | 66.55(55.65,79.03) | 1.52(1.46,1.57) |
| Solomon Islands | both | 136.18(106.67,169.84) | 475.16(379.85,589.80) | 4.16(4.03,4.28) | 199.99(167.71,236.77) | 385.07(351.19,421.37) | 2.32(2.29,2.35) |
| Somalia | both | 693.42(501.00,915.36) | 3457.52(2571.67,4519.68) | 4.82(4.37,5.26) | 57.14(52.86,61.70) | 83.63(80.86,86.47) | 1.21(1.11,1.30) |
| South Africa | both | 4461.58(3073.81,6198.66) | 7668.24(5466.64,10298.63) | 2.14(1.61,2.67) | 58.29(56.59,60.02) | 82.80(80.96,84.68) | 1.26(1.04,1.49) |
| South Sudan | both | 575.48(406.59,763.65) | 1304.79(958.04,1715.44) | 2.75(2.48,3.02) | 46.70(42.96,50.68) | 69.37(65.62,73.29) | 1.37(1.34,1.40) |
| Spain | both | 2841.34(1875.68,4053.09) | 5425.83(3663.93,7562.25) | 1.95(1.66,2.24) | 43.28(41.71,44.91) | 121.16(117.96,124.43) | 3.60(3.33,3.86) |
| Sri Lanka | both | 2070.56(1537.02,2738.80) | 4248.26(3179.86,5586.11) | 2.13(1.79,2.47) | 62.46(59.80,65.21) | 127.53(123.73,131.43) | 2.29(2.08,2.50) |
| Sudan | both | 2351.60(1713.68,3208.16) | 10452.89(7566.81,13991.39) | 5.43(5.31,5.55) | 60.91(58.47,63.43) | 125.62(123.22,128.05) | 2.78(2.64,2.92) |
| Suriname | both | 104.93(77.92,137.24) | 208.09(157.06,269.60) | 2.92(2.74,3.10) | 130.44(106.67,157.99) | 225.54(195.94,258.37) | 2.10(1.99,2.21) |
| Sweden | both | 549.95(286.48,856.64) | 1015.89(638.84,1464.90) | 2.58(2.14,3.02) | 46.61(42.79,50.68) | 89.52(84.10,95.21) | 2.17(1.87,2.48) |
| Switzerland | both | 370.67(214.29,567.54) | 874.55(571.13,1204.81) | 3.23(2.87,3.60) | 37.50(33.74,41.58) | 91.31(85.33,97.61) | 2.85(2.54,3.16) |
| Syrian Arab Republic | both | 2070.23(1533.32,2762.27) | 3690.92(2682.89,4963.97) | 1.08(0.21,1.95) | 81.73(78.24,85.34) | 121.31(117.40,125.31) | 1.07(0.96,1.17) |
| Taiwan (Province of China) | both | 3049.01(2309.49,3972.27) | 2534.18(1902.38,3248.22) | -0.94(-1.37,-0.52) | 81.22(78.35,84.16) | 88.74(85.29,92.29) | 0.04(-0.30,0.38) |
| Tajikistan | both | 673.40(482.34,903.80) | 1927.59(1411.44,2559.88) | 4.22(3.99,4.45) | 65.63(60.76,70.78) | 108.89(104.08,113.86) | 1.77(1.73,1.81) |
| Thailand | both | 6074.45(4476.96,8043.01) | 7884.98(5816.24,10517.65) | -0.16(-0.56,0.23) | 50.59(49.32,51.88) | 83.42(81.58,85.29) | 0.66(0.27,1.04) |
| Timor-Leste | both | 69.45(52.70,88.93) | 237.99(181.21,307.85) | 4.26(3.97,4.54) | 46.61(36.29,58.97) | 83.34(73.06,94.68) | 1.82(1.71,1.94) |
| Togo | both | 172.30(108.48,243.90) | 632.46(432.43,861.83) | 4.28(4.13,4.42) | 24.53(20.99,28.49) | 42.17(38.94,45.59) | 1.64(1.50,1.77) |
| Tokelau | both | 0.47(0.37,0.61) | 0.75(0.58,0.96) | 1.23(0.94,1.52) | 175.51(0.11,1797.11) | 331.73(2.83,2304.18) | 2.27(2.21,2.33) |
| Tonga | both | 32.99(25.56,42.55) | 53.94(41.68,68.82) | 1.54(1.42,1.65) | 166.11(114.11,234.18) | 286.35(214.94,374.22) | 1.79(1.71,1.87) |
| Trinidad and Tobago | both | 257.07(186.37,338.13) | 307.83(225.24,406.65) | 0.77(0.18,1.37) | 119.11(104.99,134.60) | 172.39(153.67,192.78) | 1.39(1.25,1.53) |
| Tunisia | both | 1013.23(722.10,1382.88) | 2195.29(1554.51,2962.31) | 2.72(2.43,3.01) | 59.46(55.85,63.24) | 134.39(128.82,140.13) | 2.82(2.72,2.92) |
| Turkey | both | 5777.00(4018.16,8013.45) | 11309.22(8043.97,15361.76) | 3.03(2.72,3.33) | 49.05(47.79,50.33) | 83.56(82.02,85.11) | 2.71(2.34,3.07) |
| Turkmenistan | both | 436.77(315.23,593.22) | 1011.69(745.87,1327.25) | 3.37(3.01,3.72) | 60.93(55.35,66.93) | 123.52(116.02,131.39) | 2.61(2.50,2.71) |
| Tuvalu | both | 3.03(2.35,3.80) | 7.64(5.87,9.72) | 3.61(3.44,3.78) | 190.41(39.64,557.57) | 344.74(145.31,690.46) | 2.09(2.04,2.15) |
| Uganda | both | 1995.92(1414.36,2670.00) | 7807.93(5740.21,10231.79) | 4.71(4.52,4.91) | 58.53(55.99,61.17) | 90.68(88.67,92.72) | 1.39(1.24,1.54) |
| Ukraine | both | 4574.42(3294.99,6089.09) | 3118.74(2128.15,4239.27) | -1.56(-2.20,-0.91) | 64.59(62.73,66.48) | 70.58(68.12,73.11) | -0.18(-0.42,0.06) |
| United Arab Emirates | both | 187.97(131.34,260.72) | 866.35(608.59,1184.25) | 6.26(4.25,8.31) | 64.28(55.30,74.40) | 131.24(122.65,140.28) | 2.47(2.32,2.62) |
| United Kingdom | both | 5095.68(3045.09,7488.48) | 21221.63(15215.31,28614.24) | 8.02(6.74,9.32) | 59.77(58.13,61.44) | 281.93(278.14,285.76) | 7.72(6.53,8.93) |
| United Republic of Tanzania | both | 1787.41(1213.31,2478.13) | 6826.08(4952.16,9109.18) | 4.40(4.22,4.59) | 35.30(33.68,36.98) | 61.38(59.93,62.85) | 1.79(1.67,1.91) |
| United States of America | both | 14797.35(6830.65,23786.75) | 28739.49(17314.83,41775.71) | 3.03(2.73,3.34) | 38.93(38.30,39.56) | 66.62(65.86,67.40) | 2.34(2.12,2.56) |
| United States Virgin Islands | both | 16.33(12.36,21.30) | 20.96(15.66,27.12) | 0.78(0.69,0.86) | 95.30(54.68,154.98) | 172.09(106.45,263.46) | 1.86(1.79,1.94) |
| Uruguay | both | 63.19(28.10,103.22) | 219.79(136.83,320.62) | 4.51(4.04,4.98) | 13.00(9.99,16.63) | 42.65(37.19,48.69) | 4.41(3.89,4.94) |
| Uzbekistan | both | 2251.75(1595.12,3044.04) | 5927.67(4310.76,7727.04) | 3.64(3.14,4.15) | 56.57(54.25,58.95) | 100.18(97.64,102.77) | 1.86(1.55,2.16) |
| Vanuatu | both | 49.33(37.97,62.77) | 171.90(133.63,218.64) | 4.67(4.55,4.78) | 174.80(129.42,231.11) | 311.62(266.78,361.88) | 2.14(2.10,2.18) |
| Venezuela (Bolivarian Republic of) | both | 3152.41(2386.30,4111.52) | 6759.99(5210.18,8635.61) | 3.05(2.71,3.39) | 83.92(81.01,86.90) | 157.67(153.93,161.48) | 2.14(1.81,2.47) |
| Viet Nam | both | 5568.65(4046.19,7358.39) | 9422.37(7092.54,12345.01) | 2.15(1.92,2.38) | 41.49(40.40,42.59) | 65.95(64.62,67.30) | 1.52(1.20,1.85) |
| Yemen | both | 956.37(657.62,1333.92) | 4859.65(3470.94,6601.29) | 5.05(4.38,5.72) | 43.32(40.60,46.17) | 82.61(80.30,84.97) | 1.66(0.86,2.47) |
| Zambia | both | 1017.93(733.10,1371.20) | 2976.09(2199.42,3935.11) | 3.60(3.53,3.67) | 60.73(57.04,64.60) | 78.24(75.45,81.10) | 0.65(0.50,0.80) |
| Zimbabwe | both | 1205.86(854.83,1634.79) | 3059.14(2259.07,4128.48) | 2.92(2.40,3.44) | 56.63(53.46,59.94) | 100.46(96.93,104.10) | 2.05(1.67,2.44) |
| Afghanistan | female | 1377.29(1012.32,1756.62) | 8947.91(6568.87,11674.24) | 6.17(5.57,6.77) | 118.88(112.64,125.39) | 230.85(226.09,235.69) | 2.31(2.15,2.47) |
| Albania | female | 97.59(63.75,137.32) | 101.01(67.30,139.14) | 0.09(-0.16,0.34) | 31.27(25.37,38.13) | 49.31(40.14,60.03) | 1.46(1.26,1.67) |
| Algeria | female | 1712.14(1208.07,2339.34) | 3960.30(2777.57,5515.38) | 3.00(2.35,3.65) | 66.02(62.93,69.22) | 125.08(121.21,129.05) | 2.11(1.83,2.39) |
| American Samoa | female | 13.16(9.84,17.77) | 29.24(21.85,38.99) | 2.60(2.42,2.78) | 268.65(143.55,460.32) | 545.83(366.02,784.01) | 2.44(2.24,2.65) |
| Andorra | female | 1.69(1.04,2.49) | 4.53(3.06,6.27) | 2.78(2.44,3.12) | 41.58(3.72,172.37) | 110.48(33.29,269.94) | 3.40(3.36,3.44) |
| Angola | female | 653.65(460.52,881.80) | 2604.90(1936.46,3445.18) | 4.96(4.84,5.08) | 68.35(63.20,73.81) | 90.54(87.09,94.09) | 1.03(0.94,1.11) |
| Antigua and Barbuda | female | 7.50(5.73,9.98) | 12.24(9.18,15.82) | 1.66(1.52,1.80) | 129.15(53.86,261.63) | 175.90(91.47,307.35) | 0.91(0.84,0.97) |
| Argentina | female | 1001.34(740.81,1344.34) | 3046.33(2191.70,4002.41) | 4.36(4.13,4.60) | 37.19(34.92,39.57) | 84.61(81.63,87.68) | 3.40(3.13,3.67) |
| Armenia | female | 185.87(132.46,253.37) | 182.35(127.45,246.35) | 0.49(-0.22,1.20) | 66.02(56.86,76.25) | 103.85(89.25,120.27) | 1.49(1.33,1.65) |
| Australia | female | 240.57(128.88,372.88) | 581.93(315.35,896.03) | 3.29(2.47,4.11) | 17.87(15.68,20.28) | 38.07(35.03,41.31) | 2.69(2.02,3.36) |
| Austria | female | 202.50(138.41,281.57) | 361.73(230.21,516.90) | 2.52(2.25,2.80) | 33.95(29.39,39.05) | 74.43(66.91,82.59) | 2.76(2.70,2.81) |
| Azerbaijan | female | 517.84(377.44,682.84) | 831.20(604.39,1099.47) | 2.38(1.88,2.89) | 73.28(67.09,79.89) | 114.26(106.56,122.39) | 1.45(1.29,1.61) |
| Bahamas | female | 32.65(24.40,42.60) | 58.02(43.54,75.23) | 2.11(2.05,2.17) | 120.65(82.86,169.92) | 186.16(141.34,240.83) | 1.40(1.35,1.46) |
| Bahrain | female | 25.77(17.83,35.21) | 110.44(79.89,150.33) | 5.75(5.15,6.35) | 62.54(40.69,92.39) | 140.09(115.18,168.81) | 2.74(2.59,2.89) |
| Bangladesh | female | 7539.24(5395.95,10267.08) | 16556.78(12273.49,22203.51) | 2.20(1.99,2.42) | 68.09(66.56,69.65) | 105.35(103.75,106.97) | 1.09(0.96,1.22) |
| Barbados | female | 29.72(22.76,37.37) | 36.30(27.73,46.47) | 0.76(0.61,0.92) | 130.61(87.91,187.12) | 187.93(131.83,259.95) | 1.13(1.04,1.22) |
| Belarus | female | 371.85(252.89,512.50) | 213.92(132.86,304.31) | -1.95(-2.65,-1.24) | 50.52(45.51,55.93) | 47.11(41.00,53.89) | -0.72(-0.99,-0.45) |
| Belgium | female | 315.53(206.82,447.98) | 694.78(475.42,944.60) | 2.91(2.71,3.11) | 45.47(40.58,50.81) | 108.80(100.85,117.23) | 2.89(2.80,2.98) |
| Belize | female | 19.05(14.59,24.03) | 73.49(56.11,93.72) | 4.75(4.43,5.07) | 100.68(60.60,157.65) | 172.52(135.34,216.84) | 1.79(1.51,2.08) |
| Benin | female | 158.53(108.63,222.39) | 905.38(649.83,1224.86) | 6.55(5.96,7.14) | 35.31(30.02,41.25) | 70.52(65.99,75.27) | 2.76(2.21,3.33) |
| Bermuda | female | 3.36(2.52,4.42) | 3.69(2.77,4.75) | 0.43(0.31,0.55) | 76.25(17.46,219.30) | 118.55(30.06,317.30) | 1.37(1.26,1.48) |
| Bhutan | female | 44.61(31.87,60.90) | 70.91(51.29,96.05) | 2.27(2.03,2.51) | 71.45(52.02,95.81) | 99.78(77.89,126.00) | 1.17(1.11,1.24) |
| Bolivia (Plurinational State of) | female | 285.07(202.34,390.18) | 690.71(491.73,958.14) | 3.09(2.89,3.28) | 46.74(41.47,52.51) | 64.96(60.21,70.00) | 1.01(0.94,1.09) |
| Bosnia and Herzegovina | female | 165.05(112.12,232.53) | 154.28(106.28,222.33) | 0.29(-0.23,0.81) | 42.68(36.42,49.73) | 74.97(63.57,87.90) | 1.98(1.81,2.16) |
| Botswana | female | 73.97(50.76,103.79) | 187.81(135.71,259.33) | 3.28(3.03,3.53) | 52.42(41.13,65.89) | 88.74(76.50,102.39) | 1.93(1.88,1.98) |
| Brazil | female | 10010.60(7160.78,13717.25) | 14194.84(9684.58,19670.12) | 1.45(1.32,1.58) | 67.93(66.60,69.27) | 83.44(82.07,84.83) | 0.97(0.76,1.18) |
| Brunei Darussalam | female | 40.86(30.93,53.36) | 58.25(43.13,77.29) | 1.11(0.94,1.29) | 166.55(119.40,226.46) | 162.74(123.58,210.66) | -0.35(-0.61,-0.09) |
| Bulgaria | female | 327.67(233.42,436.58) | 231.84(158.70,315.31) | -2.17(-2.59,-1.74) | 55.71(49.84,62.09) | 76.67(67.12,87.21) | 0.17(-0.15,0.49) |
| Burkina Faso | female | 375.34(251.12,514.01) | 1448.10(1020.84,1971.79) | 4.68(4.61,4.75) | 43.07(38.82,47.68) | 65.71(62.36,69.19) | 1.35(1.30,1.40) |
| Burundi | female | 298.33(211.02,396.04) | 766.63(544.09,1049.74) | 3.44(3.29,3.58) | 58.08(51.68,65.07) | 64.26(59.78,68.99) | -0.06(-0.19,0.08) |
| Cabo Verde | female | 14.99(9.84,20.94) | 41.10(28.88,55.85) | 4.02(3.34,4.70) | 42.59(23.83,70.36) | 81.96(58.83,111.27) | 2.26(1.96,2.57) |
| Cambodia | female | 494.33(380.39,626.95) | 1303.98(974.48,1698.13) | 3.59(3.47,3.71) | 49.85(45.55,54.45) | 90.08(85.25,95.11) | 1.83(1.60,2.06) |
| Cameroon | female | 310.56(206.28,436.56) | 1782.94(1276.33,2357.01) | 7.21(5.79,8.66) | 30.71(27.39,34.33) | 59.76(57.02,62.60) | 3.24(1.78,4.72) |
| Canada | female | 128.81(37.94,244.55) | 527.50(176.60,984.71) | 3.94(3.27,4.61) | 6.72(5.61,7.99) | 25.51(23.37,27.79) | 3.39(2.61,4.17) |
| Central African Republic | female | 197.50(139.46,265.85) | 657.07(478.72,871.80) | 4.21(3.94,4.49) | 73.07(63.24,84.01) | 117.84(109.00,127.22) | 1.63(1.48,1.78) |
| Chad | female | 204.48(136.82,286.93) | 1055.23(742.52,1424.81) | 5.75(5.69,5.82) | 36.70(31.83,42.12) | 66.28(62.32,70.43) | 2.06(1.96,2.17) |
| Chile | female | 538.41(378.63,739.94) | 1053.34(745.23,1465.03) | 2.72(2.50,2.95) | 41.85(38.39,45.54) | 80.31(75.52,85.33) | 2.24(2.10,2.39) |
| China | female | 106103.59(76263.91,142849.35) | 82231.39(58387.48,112399.03) | 2.09(1.11,3.09) | 82.62(82.12,83.12) | 107.82(107.08,108.56) | 3.14(2.47,3.81) |
| Colombia | female | 3041.66(2285.04,4076.77) | 6146.81(4618.93,8096.10) | 2.18(1.98,2.37) | 89.17(86.03,92.40) | 152.90(149.10,156.78) | 1.55(1.36,1.73) |
| Comoros | female | 20.96(14.58,28.32) | 36.58(25.74,50.79) | 1.64(1.53,1.74) | 45.11(27.87,69.28) | 53.45(37.55,73.84) | 0.35(0.26,0.44) |
| Congo | female | 208.65(149.99,281.17) | 516.74(382.96,708.99) | 2.85(2.52,3.18) | 83.53(72.56,95.72) | 107.57(98.48,117.27) | 0.72(0.54,0.89) |
| Cook Islands | female | 3.04(2.34,3.91) | 4.48(3.39,5.73) | 1.08(0.90,1.26) | 169.20(35.31,495.97) | 316.65(94.59,776.77) | 2.15(2.10,2.19) |
| Costa Rica | female | 218.03(160.73,284.43) | 617.43(469.44,810.08) | 3.68(3.42,3.94) | 74.50(64.94,85.09) | 159.87(147.46,173.06) | 2.44(2.33,2.55) |
| Croatia | female | 153.47(105.49,214.18) | 169.41(112.55,231.78) | -0.13(-0.33,0.07) | 44.84(38.02,52.55) | 71.09(60.74,82.79) | 1.17(1.03,1.30) |
| Cuba | female | 1311.98(975.27,1696.60) | 1113.55(826.92,1464.74) | -0.43(-0.84,-0.02) | 116.27(110.06,122.75) | 164.07(154.56,174.01) | 0.91(0.71,1.12) |
| Cyprus | female | 27.49(18.47,38.40) | 73.84(48.40,103.13) | 3.78(3.40,4.16) | 45.41(30.03,66.01) | 100.12(78.39,126.32) | 2.61(2.53,2.69) |
| Czechia | female | 387.37(267.44,540.14) | 454.51(308.55,643.70) | 0.00(-0.39,0.39) | 53.97(48.72,59.65) | 97.19(88.45,106.56) | 1.78(1.68,1.88) |
| Côte d'Ivoire | female | 451.35(303.61,629.80) | 1784.49(1304.79,2386.79) | 4.58(4.07,5.09) | 37.90(34.48,41.56) | 73.37(70.00,76.85) | 2.17(1.93,2.40) |
| Democratic People's Republic of Korea | female | 1707.54(1276.57,2236.82) | 2267.23(1720.95,2962.62) | 1.33(1.12,1.55) | 81.62(77.79,85.59) | 117.77(112.96,122.75) | 1.16(1.03,1.29) |
| Democratic Republic of the Congo | female | 2106.42(1463.37,2855.83) | 8165.72(5865.49,11077.23) | 4.81(4.55,5.07) | 58.37(55.90,60.92) | 93.83(91.80,95.89) | 1.55(1.31,1.80) |
| Denmark | female | 170.96(113.49,239.87) | 380.72(267.28,522.11) | 2.92(2.52,3.32) | 45.22(38.68,52.57) | 109.79(99.00,121.45) | 2.99(2.90,3.07) |
| Djibouti | female | 19.81(13.46,27.96) | 58.42(40.67,79.80) | 4.22(3.88,4.57) | 42.16(25.68,65.27) | 63.39(48.18,81.94) | 1.36(1.32,1.40) |
| Dominica | female | 9.88(7.49,12.68) | 11.16(8.47,14.08) | 0.40(0.14,0.66) | 139.97(66.73,259.20) | 209.93(105.40,374.56) | 0.98(0.67,1.30) |
| Dominican Republic | female | 523.25(402.70,667.59) | 1138.42(877.42,1441.62) | 3.05(2.88,3.21) | 65.16(59.69,70.99) | 116.59(109.91,123.57) | 2.23(2.11,2.35) |
| Ecuador | female | 426.21(288.75,600.37) | 1270.17(903.49,1683.81) | 2.89(2.49,3.29) | 41.48(37.63,45.62) | 78.67(74.40,83.12) | 1.37(1.01,1.74) |
| Egypt | female | 1997.79(1386.76,2772.71) | 7724.76(5445.39,10494.36) | 5.17(4.99,5.35) | 39.44(37.73,41.21) | 87.79(85.84,89.77) | 3.01(2.80,3.22) |
| El Salvador | female | 463.75(349.77,590.63) | 925.27(697.64,1176.56) | 2.33(2.18,2.48) | 82.75(75.38,90.65) | 150.22(140.68,160.27) | 1.97(1.85,2.08) |
| Equatorial Guinea | female | 26.64(18.70,36.04) | 132.90(94.94,180.00) | 5.74(5.64,5.85) | 66.39(43.60,97.01) | 94.90(79.44,112.52) | 1.15(1.10,1.20) |
| Eritrea | female | 148.48(100.83,203.30) | 498.07(354.07,684.87) | 4.27(4.12,4.43) | 51.68(43.69,60.73) | 71.52(65.37,78.09) | 1.02(0.98,1.05) |
| Estonia | female | 46.80(30.40,66.01) | 41.35(27.38,58.51) | -0.32(-0.89,0.24) | 45.04(33.07,59.93) | 65.65(47.17,89.14) | 1.08(0.82,1.34) |
| Eswatini | female | 46.24(32.40,63.76) | 99.13(70.95,132.93) | 2.84(2.01,3.68) | 53.42(39.12,71.31) | 83.73(68.06,101.93) | 1.74(1.23,2.25) |
| Ethiopia | female | 3508.43(2557.06,4640.52) | 6051.87(4180.90,8331.94) | 1.39(1.22,1.57) | 72.28(69.91,74.71) | 53.11(51.78,54.47) | -1.45(-1.57,-1.32) |
| Fiji | female | 140.82(107.78,179.28) | 252.61(193.29,319.11) | 1.80(1.39,2.21) | 192.80(162.27,227.41) | 340.84(300.09,385.60) | 1.71(1.46,1.96) |
| Finland | female | 240.96(150.92,355.25) | 551.75(389.13,747.56) | 3.10(2.86,3.34) | 73.99(64.90,84.03) | 184.43(169.34,200.53) | 3.12(2.84,3.40) |
| France | female | 1010.76(663.78,1416.81) | 2415.41(1539.66,3519.19) | 2.71(1.86,3.57) | 23.22(21.81,24.70) | 62.14(59.69,64.67) | 3.04(2.20,3.88) |
| Gabon | female | 58.92(41.72,80.93) | 174.22(126.22,236.53) | 3.76(3.58,3.93) | 61.50(46.80,79.42) | 96.08(82.34,111.46) | 1.43(1.30,1.57) |
| Gambia | female | 30.11(20.05,43.27) | 144.80(102.78,190.98) | 5.43(5.31,5.56) | 30.53(20.61,43.58) | 59.12(49.88,69.61) | 2.34(2.26,2.41) |
| Georgia | female | 320.25(223.33,434.46) | 243.44(174.89,330.14) | -0.42(-0.86,0.03) | 77.52(69.26,86.51) | 123.66(108.58,140.32) | 1.97(1.80,2.13) |
| Germany | female | 3660.45(2594.76,4960.82) | 6744.40(4840.57,9067.64) | 1.93(1.77,2.08) | 68.12(65.89,70.42) | 157.75(153.99,161.58) | 2.36(2.17,2.54) |
| Ghana | female | 646.11(436.48,892.75) | 2385.08(1675.21,3197.46) | 4.44(4.03,4.85) | 44.44(41.08,48.00) | 73.58(70.66,76.60) | 1.41(0.99,1.83) |
| Greece | female | 358.96(245.57,490.48) | 627.27(419.39,879.52) | 1.34(1.11,1.56) | 46.08(41.43,51.11) | 124.25(114.71,134.38) | 3.24(3.11,3.37) |
| Greenland | female | 0.30(0.05,0.72) | 1.04(0.39,1.77) | 5.82(4.92,6.72) | 6.36(0.00,120.60) | 27.32(0.77,154.56) | 5.33(4.68,5.98) |
| Grenada | female | 13.50(10.32,17.30) | 18.53(13.88,23.49) | 0.89(0.51,1.27) | 170.77(92.09,290.30) | 212.19(126.59,335.32) | 0.64(0.55,0.73) |
| Guam | female | 18.46(14.35,23.72) | 33.48(25.81,42.83) | 2.15(1.98,2.31) | 152.32(90.87,240.37) | 258.34(178.29,362.56) | 1.82(1.70,1.95) |
| Guatemala | female | 656.55(492.10,848.62) | 3697.54(2771.62,4786.61) | 6.35(6.00,6.70) | 87.34(80.77,94.30) | 197.88(191.55,204.36) | 2.89(2.69,3.09) |
| Guinea | female | 171.38(110.89,238.35) | 761.93(549.67,1012.59) | 5.20(5.05,5.36) | 31.33(26.82,36.40) | 59.83(55.66,64.24) | 2.15(1.96,2.34) |
| Guinea-Bissau | female | 48.47(34.19,66.36) | 168.14(121.46,224.56) | 4.48(4.22,4.75) | 48.73(35.97,64.61) | 84.88(72.53,98.74) | 1.90(1.77,2.04) |
| Guyana | female | 145.77(109.40,185.62) | 191.38(141.47,242.73) | 0.66(0.44,0.88) | 171.88(145.11,202.17) | 247.85(213.87,285.85) | 1.00(0.81,1.18) |
| Haiti | female | 1316.60(1017.65,1660.92) | 3140.42(2382.84,4002.50) | 2.97(2.65,3.28) | 216.94(205.36,229.00) | 264.84(255.66,274.27) | 0.55(0.50,0.60) |
| Honduras | female | 478.00(361.75,632.61) | 2059.05(1555.40,2691.92) | 5.09(4.94,5.24) | 106.15(96.81,116.16) | 196.28(187.89,204.94) | 1.99(1.91,2.08) |
| Hungary | female | 390.81(271.29,540.56) | 365.32(249.12,508.91) | -0.58(-0.82,-0.35) | 55.85(50.45,61.68) | 72.71(65.44,80.60) | 0.91(0.83,0.98) |
| Iceland | female | 9.32(6.11,13.26) | 27.68(19.68,37.79) | 4.10(4.00,4.21) | 44.58(20.70,84.11) | 122.85(81.33,178.57) | 3.57(3.53,3.61) |
| India | female | 59208.78(42441.96,80138.50) | 156641.04(111712.09,214036.52) | 2.85(2.56,3.14) | 76.65(76.03,77.27) | 123.70(123.09,124.31) | 0.99(0.68,1.31) |
| Indonesia | female | 10417.52(7835.79,13493.91) | 15418.84(11402.14,20340.10) | 0.15(-0.90,1.22) | 54.99(53.94,56.05) | 70.12(69.01,71.23) | -0.13(-1.16,0.90) |
| Iran (Islamic Republic of) | female | 2952.40(1973.50,4140.16) | 5025.71(3332.75,7095.08) | 2.32(1.30,3.35) | 51.96(50.10,53.87) | 89.20(86.75,91.71) | 1.93(1.57,2.28) |
| Iraq | female | 2363.09(1755.14,3060.00) | 7832.24(5889.46,10010.71) | 4.05(3.70,4.40) | 144.82(139.00,150.82) | 183.05(179.02,187.15) | 0.79(0.53,1.05) |
| Ireland | female | 98.02(58.27,146.48) | 265.60(172.73,372.45) | 3.37(3.23,3.51) | 32.75(26.57,39.97) | 88.99(78.61,100.37) | 3.58(3.50,3.67) |
| Israel | female | 187.83(128.02,265.88) | 761.36(540.68,993.34) | 3.55(2.39,4.71) | 44.79(38.60,51.71) | 112.41(104.56,120.68) | 2.11(0.92,3.32) |
| Italy | female | 2078.30(1070.85,3258.55) | 2336.68(1348.14,3479.74) | -1.56(-3.98,0.92) | 46.49(44.51,48.54) | 82.50(79.19,85.92) | -0.04(-2.35,2.33) |
| Jamaica | female | 268.85(201.04,347.24) | 480.79(364.27,635.22) | 2.11(1.64,2.59) | 108.20(95.65,121.94) | 188.12(171.64,205.81) | 1.74(1.28,2.20) |
| Japan | female | 3358.35(2323.17,4608.50) | 3845.67(2721.49,5262.72) | -0.33(-0.66,0.01) | 35.77(34.57,37.00) | 64.40(62.38,66.47) | 1.69(1.49,1.89) |
| Jordan | female | 253.55(190.22,334.60) | 1208.99(882.77,1657.33) | 5.31(5.02,5.60) | 64.15(56.48,72.58) | 115.23(108.82,121.92) | 1.87(1.77,1.96) |
| Kazakhstan | female | 1357.76(978.70,1822.59) | 1809.68(1309.51,2449.90) | 2.84(2.11,3.58) | 100.30(95.03,105.78) | 146.62(139.93,153.55) | 2.56(2.10,3.04) |
| Kenya | female | 873.54(571.74,1237.44) | 2885.55(1965.68,3979.19) | 3.90(3.67,4.12) | 36.26(33.89,38.75) | 53.72(51.77,55.72) | 1.20(1.11,1.30) |
| Kiribati | female | 18.78(14.60,23.97) | 42.66(32.12,53.70) | 3.35(3.09,3.61) | 253.77(152.15,399.23) | 388.01(280.41,523.47) | 1.39(1.24,1.54) |
| Kuwait | female | 141.68(100.47,193.97) | 467.28(333.87,648.93) | 4.57(4.29,4.85) | 90.12(75.80,106.50) | 170.23(155.01,186.64) | 1.62(1.35,1.90) |
| Kyrgyzstan | female | 227.91(159.18,305.60) | 494.08(352.39,684.97) | 3.10(2.81,3.39) | 57.20(50.01,65.14) | 89.38(81.65,97.66) | 1.53(1.48,1.57) |
| Lao People's Democratic Republic | female | 331.75(248.70,429.36) | 797.39(603.41,1026.75) | 3.10(2.83,3.38) | 83.28(74.54,92.77) | 114.41(106.61,122.64) | 0.95(0.85,1.04) |
| Latvia | female | 87.73(59.49,122.72) | 49.90(32.45,69.50) | -1.38(-2.04,-0.73) | 48.29(38.72,59.55) | 58.49(43.40,77.17) | 0.63(0.47,0.79) |
| Lebanon | female | 195.17(136.48,270.68) | 406.24(288.09,550.21) | 3.29(2.92,3.65) | 69.72(60.28,80.24) | 122.05(110.46,134.55) | 2.03(1.93,2.12) |
| Lesotho | female | 79.73(54.16,110.85) | 178.88(127.48,243.14) | 2.90(2.62,3.18) | 43.90(34.78,54.72) | 83.92(72.08,97.17) | 2.53(2.42,2.65) |
| Liberia | female | 86.64(61.21,116.47) | 405.84(290.49,541.80) | 5.17(4.78,5.55) | 51.21(40.84,63.55) | 82.36(74.53,90.79) | 1.58(1.51,1.66) |
| Libya | female | 300.41(209.00,418.30) | 806.11(560.92,1084.20) | 3.25(2.83,3.67) | 75.09(66.81,84.13) | 139.08(129.64,149.03) | 2.11(2.03,2.19) |
| Lithuania | female | 88.20(57.40,123.11) | 71.06(44.24,102.60) | -0.15(-0.65,0.35) | 31.76(25.47,39.14) | 44.88(35.03,56.82) | 1.50(1.15,1.86) |
| Luxembourg | female | 11.84(8.10,16.31) | 40.56(27.53,56.07) | 4.93(4.62,5.24) | 46.30(23.52,83.12) | 113.73(81.33,155.17) | 3.44(3.26,3.62) |
| Madagascar | female | 603.60(419.31,815.61) | 1804.06(1292.09,2495.69) | 3.65(3.56,3.75) | 51.87(47.81,56.19) | 66.30(63.27,69.44) | 0.69(0.62,0.76) |
| Malawi | female | 561.37(389.91,772.53) | 1533.59(1071.91,2118.43) | 2.99(2.73,3.24) | 58.36(53.63,63.40) | 74.90(71.18,78.77) | 0.49(0.32,0.66) |
| Malaysia | female | 1200.62(872.97,1627.55) | 3286.02(2454.30,4369.67) | 3.54(3.30,3.78) | 71.31(67.34,75.47) | 119.14(115.10,123.29) | 1.60(1.51,1.70) |
| Maldives | female | 10.99(8.09,14.60) | 26.76(19.88,35.57) | 3.59(3.20,3.97) | 50.24(25.06,90.12) | 87.85(57.68,128.73) | 1.86(1.69,2.03) |
| Mali | female | 325.08(221.21,445.83) | 1477.21(1075.46,1987.06) | 5.32(5.26,5.37) | 41.94(37.50,46.77) | 66.36(63.01,69.84) | 1.40(1.32,1.48) |
| Malta | female | 17.52(11.80,24.93) | 35.38(24.70,47.53) | 2.34(1.98,2.71) | 69.78(41.00,111.21) | 156.89(109.24,219.06) | 2.65(2.57,2.73) |
| Marshall Islands | female | 12.26(9.09,16.15) | 28.87(21.28,37.70) | 2.59(1.96,3.23) | 294.19(152.88,514.09) | 578.32(386.80,832.15) | 2.26(1.98,2.54) |
| Mauritania | female | 55.12(36.42,76.91) | 187.94(126.81,263.13) | 4.14(3.97,4.30) | 28.08(21.16,36.58) | 44.66(38.50,51.55) | 1.51(1.36,1.66) |
| Mauritius | female | 90.97(67.97,119.33) | 168.36(124.87,217.76) | 3.17(2.78,3.55) | 87.42(70.37,107.44) | 178.77(152.78,207.99) | 3.55(3.07,4.03) |
| Mexico | female | 13012.37(9854.57,17015.81) | 22734.96(17177.28,29459.34) | 2.15(1.72,2.57) | 139.59(137.20,142.01) | 209.00(206.30,211.74) | 1.69(1.28,2.11) |
| Micronesia (Federated States of) | female | 15.61(11.96,19.90) | 32.21(24.72,40.64) | 2.22(1.44,3.00) | 162.43(91.70,267.66) | 325.58(222.91,459.72) | 2.36(1.67,3.05) |
| Monaco | female | 0.69(0.42,1.02) | 2.04(1.38,2.80) | 4.32(3.99,4.66) | 42.97(0.21,369.59) | 116.87(14.57,419.13) | 3.49(3.45,3.53) |
| Mongolia | female | 84.88(60.27,115.44) | 137.64(95.99,186.48) | 2.08(1.73,2.43) | 39.10(31.22,48.37) | 58.91(49.44,69.71) | 1.43(1.36,1.51) |
| Montenegro | female | 27.93(19.19,38.66) | 34.15(23.29,46.24) | 0.39(0.09,0.70) | 55.54(36.88,80.31) | 88.26(61.16,123.31) | 1.41(1.27,1.56) |
| Morocco | female | 1459.79(1026.93,1997.59) | 3760.13(2706.80,5105.83) | 3.44(3.13,3.74) | 57.16(54.26,60.17) | 124.29(120.35,128.33) | 2.81(2.71,2.90) |
| Mozambique | female | 584.28(402.21,792.51) | 2349.37(1690.70,3212.18) | 4.50(4.15,4.85) | 46.77(43.04,50.74) | 74.85(71.85,77.95) | 1.56(1.32,1.79) |
| Myanmar | female | 3519.93(2603.72,4519.89) | 4756.35(3512.74,6231.55) | 0.70(0.60,0.79) | 86.53(83.69,89.44) | 97.76(95.00,100.58) | 0.06(-0.04,0.16) |
| Namibia | female | 80.49(56.09,112.64) | 187.48(134.08,252.48) | 2.85(2.66,3.04) | 53.73(42.62,66.88) | 78.78(67.91,90.91) | 1.20(1.12,1.29) |
| Nauru | female | 1.42(1.10,1.78) | 2.94(2.30,3.71) | 2.36(2.16,2.56) | 153.74(9.84,686.11) | 286.48(57.63,851.42) | 2.09(1.95,2.22) |
| Nepal | female | 1155.33(843.31,1555.09) | 3792.63(2798.14,5125.27) | 3.69(3.38,4.00) | 62.58(59.02,66.29) | 114.71(111.08,118.42) | 1.57(1.21,1.93) |
| Netherlands | female | 433.65(278.32,616.75) | 875.92(590.92,1197.07) | 3.20(2.58,3.83) | 36.70(33.31,40.35) | 85.06(79.52,90.90) | 3.42(3.05,3.78) |
| New Zealand | female | 57.63(20.57,101.47) | 145.36(78.70,221.90) | 2.69(1.85,3.55) | 20.23(15.35,26.18) | 50.29(42.45,59.18) | 2.33(1.52,3.15) |
| Nicaragua | female | 391.36(292.08,517.06) | 1080.32(806.87,1415.78) | 3.46(3.27,3.64) | 98.80(89.24,109.13) | 175.92(165.58,186.74) | 1.92(1.83,2.01) |
| Niger | female | 136.98(81.95,201.25) | 960.77(662.51,1302.91) | 6.53(6.07,6.99) | 18.41(15.45,21.78) | 41.98(39.36,44.74) | 2.70(2.38,3.02) |
| Nigeria | female | 2720.59(1737.73,3898.26) | 8231.19(5180.66,11729.96) | 3.66(3.54,3.78) | 29.95(28.84,31.10) | 36.04(35.27,36.83) | 0.57(0.46,0.67) |
| Niue | female | 0.36(0.27,0.47) | 0.50(0.38,0.64) | 1.01(0.71,1.31) | 205.80(0.01,2608.48) | 425.08(0.42,4000.24) | 2.92(2.79,3.05) |
| North Macedonia | female | 82.89(55.98,116.93) | 101.79(68.57,143.12) | 0.81(0.54,1.08) | 49.66(39.55,61.59) | 75.38(61.40,91.75) | 1.28(1.21,1.36) |
| Northern Mariana Islands | female | 7.92(6.03,10.29) | 9.88(7.54,12.81) | 0.36(-0.93,1.65) | 158.06(67.33,319.99) | 271.57(129.22,504.69) | 1.82(1.77,1.88) |
| Norway | female | 208.76(129.05,300.56) | 324.34(209.12,460.05) | -0.04(-0.58,0.50) | 64.79(56.28,74.25) | 100.23(89.60,111.79) | -0.36(-0.86,0.14) |
| Oman | female | 80.30(57.28,108.84) | 297.87(214.55,403.29) | 4.02(3.52,4.52) | 62.13(49.26,77.39) | 107.81(95.88,120.87) | 1.47(0.93,2.01) |
| Pakistan | female | 8367.47(6091.58,11101.65) | 30711.27(22411.70,40165.12) | 4.21(3.92,4.49) | 83.01(81.23,84.81) | 138.42(136.88,139.98) | 1.51(1.27,1.74) |
| Palau | female | 2.53(1.91,3.29) | 3.47(2.58,4.46) | 1.02(0.62,1.42) | 170.66(28.81,545.10) | 337.39(80.60,927.29) | 2.36(2.29,2.43) |
| Palestine | female | 119.70(84.97,164.31) | 526.53(373.52,720.04) | 5.43(5.32,5.55) | 60.77(50.36,72.73) | 105.78(96.93,115.21) | 1.75(1.58,1.91) |
| Panama | female | 194.97(145.40,253.54) | 563.33(420.88,737.42) | 3.56(3.41,3.71) | 80.56(69.64,92.69) | 162.78(149.60,176.81) | 2.37(2.29,2.44) |
| Papua New Guinea | female | 638.92(488.79,836.81) | 2628.72(1992.49,3399.96) | 5.12(5.02,5.23) | 162.78(150.40,175.92) | 291.73(280.68,303.11) | 2.00(1.88,2.11) |
| Paraguay | female | 190.74(130.89,264.75) | 417.48(282.90,587.24) | 2.88(2.61,3.15) | 51.11(44.11,58.92) | 65.79(59.62,72.42) | 0.83(0.60,1.07) |
| Peru | female | 630.58(436.65,873.68) | 1562.74(1094.75,2120.93) | 3.06(2.91,3.21) | 28.27(26.10,30.56) | 56.52(53.75,59.40) | 2.26(2.09,2.44) |
| Philippines | female | 3797.54(2793.37,5024.79) | 7970.61(6005.33,10384.25) | 2.09(1.92,2.26) | 59.42(57.55,61.34) | 77.68(75.99,79.41) | 0.39(0.20,0.58) |
| Poland | female | 1310.76(887.34,1849.27) | 1555.32(1031.68,2197.83) | 0.79(0.48,1.11) | 51.52(48.77,54.39) | 78.08(74.23,82.08) | 1.72(1.14,2.31) |
| Portugal | female | 551.49(380.75,759.57) | 871.13(611.43,1185.72) | 0.89(0.60,1.17) | 67.57(62.05,73.46) | 154.56(144.46,165.18) | 2.62(2.49,2.76) |
| Puerto Rico | female | 401.81(294.41,543.66) | 508.89(375.25,666.62) | 0.81(0.72,0.91) | 127.59(115.41,140.70) | 220.72(201.95,240.79) | 1.87(1.77,1.96) |
| Qatar | female | 18.43(12.98,25.50) | 130.65(91.69,180.79) | 8.64(7.96,9.32) | 79.98(47.71,125.99) | 145.93(121.95,173.35) | 2.78(2.48,3.07) |
| Republic of Korea | female | 2288.53(1674.71,3048.64) | 2654.37(1938.53,3555.17) | 0.93(0.50,1.36) | 52.06(49.95,54.24) | 89.46(86.05,92.97) | 2.28(1.72,2.85) |
| Republic of Moldova | female | 189.22(128.51,261.09) | 141.64(91.01,203.05) | -0.52(-1.15,0.11) | 59.56(51.38,68.69) | 68.63(57.78,80.99) | 0.64(0.46,0.81) |
| Romania | female | 841.81(586.80,1153.22) | 551.32(360.77,807.29) | -2.15(-2.41,-1.89) | 43.59(40.69,46.64) | 56.14(51.55,61.03) | 0.53(0.38,0.68) |
| Russian Federation | female | 4095.61(2709.59,5752.56) | 3016.10(1814.58,4439.22) | -1.30(-2.11,-0.48) | 40.76(39.53,42.03) | 42.38(40.89,43.92) | -0.17(-0.38,0.03) |
| Rwanda | female | 417.06(298.12,564.62) | 731.60(502.64,1009.87) | 1.70(1.16,2.24) | 60.84(55.13,67.00) | 55.33(51.39,59.50) | -1.06(-1.37,-0.76) |
| Saint Kitts and Nevis | female | 5.49(4.11,7.14) | 7.60(5.60,9.98) | 1.08(1.00,1.16) | 137.87(47.73,311.60) | 169.13(71.08,340.45) | 0.41(0.24,0.59) |
| Saint Lucia | female | 30.21(22.65,38.31) | 35.95(26.72,46.87) | 0.76(0.65,0.87) | 208.85(141.10,298.07) | 262.25(183.53,363.93) | 0.85(0.69,1.00) |
| Saint Vincent and the Grenadines | female | 21.27(16.32,26.80) | 21.53(16.38,27.53) | -0.28(-0.41,-0.15) | 186.82(115.96,285.51) | 244.58(152.40,372.27) | 0.69(0.60,0.78) |
| Samoa | female | 25.06(18.89,31.89) | 59.29(44.79,77.03) | 2.79(2.66,2.93) | 155.56(100.47,230.70) | 301.14(229.11,389.06) | 2.26(2.11,2.41) |
| San Marino | female | 0.88(0.55,1.28) | 2.42(1.64,3.34) | 3.52(3.38,3.66) | 43.64(0.69,277.22) | 114.88(18.26,380.69) | 3.42(3.39,3.46) |
| Sao Tome and Principe | female | 4.77(3.18,6.85) | 14.21(9.96,19.68) | 3.59(3.40,3.77) | 41.31(12.88,99.97) | 68.74(37.71,115.51) | 1.71(1.64,1.78) |
| Saudi Arabia | female | 1053.23(741.74,1401.35) | 4648.43(3287.75,6354.25) | 6.11(5.65,6.58) | 75.50(71.01,80.21) | 171.34(166.42,176.38) | 3.37(3.08,3.66) |
| Senegal | female | 408.42(282.49,574.45) | 1340.68(966.70,1792.57) | 4.22(3.68,4.77) | 54.50(49.34,60.08) | 89.49(84.76,94.42) | 1.84(1.39,2.28) |
| Serbia | female | 347.03(234.00,477.23) | 365.61(242.86,518.84) | -0.47(-0.71,-0.22) | 53.24(47.78,59.15) | 70.22(63.20,77.82) | 0.71(0.63,0.78) |
| Seychelles | female | 4.58(3.36,6.10) | 9.78(7.13,13.01) | 2.74(2.66,2.82) | 62.60(19.05,151.36) | 142.82(67.81,265.34) | 2.96(2.92,3.00) |
| Sierra Leone | female | 66.47(39.31,95.57) | 402.14(286.17,532.13) | 7.27(6.92,7.62) | 19.17(14.84,24.37) | 44.94(40.65,49.56) | 3.27(3.09,3.45) |
| Singapore | female | 210.88(148.29,286.04) | 318.87(226.86,428.42) | 2.53(1.98,3.09) | 78.96(68.57,90.52) | 117.53(104.86,131.39) | 1.38(1.16,1.60) |
| Slovakia | female | 153.29(105.64,211.30) | 173.34(112.88,245.27) | 0.26(-0.16,0.69) | 39.80(33.74,46.63) | 60.35(51.68,70.12) | 1.37(1.34,1.39) |
| Slovenia | female | 59.83(40.86,82.92) | 61.51(40.97,88.02) | 0.03(-0.26,0.31) | 40.38(30.79,52.09) | 64.43(49.32,82.84) | 1.61(1.56,1.65) |
| Solomon Islands | female | 62.60(48.02,78.80) | 225.55(176.95,282.63) | 4.30(4.17,4.42) | 185.76(142.52,238.18) | 372.10(325.10,424.02) | 2.45(2.43,2.46) |
| Somalia | female | 315.45(226.69,421.40) | 1490.32(1069.06,2052.35) | 4.60(4.19,5.01) | 54.79(48.78,61.36) | 75.12(71.34,79.04) | 0.89(0.79,0.98) |
| South Africa | female | 2709.72(1920.41,3737.07) | 4111.85(2910.17,5551.28) | 1.70(1.08,2.32) | 68.16(65.62,70.78) | 90.32(87.58,93.13) | 1.01(0.70,1.33) |
| South Sudan | female | 247.35(170.38,342.56) | 593.12(417.16,799.98) | 2.85(2.63,3.08) | 42.54(37.40,48.20) | 63.03(58.03,68.35) | 1.29(1.23,1.35) |
| Spain | female | 1377.06(889.20,1989.82) | 2788.86(1900.01,3900.98) | 2.61(2.27,2.96) | 42.82(40.59,45.15) | 127.79(123.09,132.62) | 4.30(3.88,4.72) |
| Sri Lanka | female | 1035.30(761.71,1396.00) | 2196.76(1640.65,2909.11) | 2.34(2.00,2.67) | 62.58(58.82,66.51) | 131.10(125.67,136.70) | 2.45(2.25,2.65) |
| Sudan | female | 1218.32(882.62,1677.14) | 4995.68(3600.65,6637.00) | 5.16(4.98,5.34) | 61.48(58.08,65.04) | 123.11(119.72,126.57) | 2.66(2.52,2.81) |
| Suriname | female | 57.09(43.15,72.82) | 113.33(84.65,146.41) | 2.81(2.68,2.95) | 148.85(112.75,192.92) | 248.91(205.19,299.25) | 1.87(1.75,1.98) |
| Sweden | female | 269.43(154.93,406.15) | 469.48(292.49,674.13) | 2.39(2.06,2.73) | 46.96(41.51,52.94) | 86.34(78.70,94.52) | 2.05(1.87,2.22) |
| Switzerland | female | 186.73(113.08,280.65) | 442.42(298.20,605.99) | 3.26(2.92,3.60) | 39.58(34.05,45.79) | 96.80(87.94,106.34) | 2.88(2.57,3.19) |
| Syrian Arab Republic | female | 1048.37(760.75,1413.29) | 1897.12(1359.19,2546.55) | 1.15(0.41,1.89) | 84.12(79.09,89.39) | 117.21(111.98,122.62) | 0.81(0.68,0.93) |
| Taiwan (Province of China) | female | 1320.87(993.78,1754.97) | 1065.12(797.84,1386.64) | -1.13(-1.52,-0.73) | 72.21(68.37,76.23) | 77.85(73.21,82.72) | -0.07(-0.35,0.22) |
| Tajikistan | female | 358.53(257.37,481.33) | 999.16(742.20,1325.66) | 4.18(3.95,4.41) | 69.07(62.10,76.61) | 115.47(108.42,122.88) | 1.85(1.80,1.91) |
| Thailand | female | 2868.28(2112.69,3824.36) | 3812.81(2830.81,5125.06) | -0.26(-0.71,0.19) | 48.57(46.81,50.38) | 81.71(79.12,84.36) | 0.56(0.11,1.01) |
| Timor-Leste | female | 32.39(24.26,42.05) | 111.67(84.08,146.19) | 4.32(4.04,4.60) | 45.22(31.00,63.85) | 78.56(64.64,94.61) | 1.66(1.53,1.80) |
| Togo | female | 88.24(56.93,124.37) | 310.25(212.16,422.83) | 4.10(3.94,4.27) | 24.44(19.61,30.13) | 42.03(37.48,46.98) | 1.60(1.42,1.77) |
| Tokelau | female | 0.21(0.16,0.27) | 0.34(0.26,0.45) | 1.05(0.59,1.51) | 162.09(0.00,3369.53) | 310.40(0.01,4083.07) | 2.26(2.20,2.31) |
| Tonga | female | 14.97(11.51,19.22) | 23.64(17.90,30.23) | 1.29(1.18,1.41) | 155.58(86.80,258.32) | 253.80(161.89,379.56) | 1.46(1.38,1.55) |
| Trinidad and Tobago | female | 148.36(107.96,196.25) | 159.18(116.86,214.89) | 0.32(-0.26,0.90) | 138.40(117.03,162.56) | 181.29(154.22,211.79) | 0.96(0.85,1.06) |
| Tunisia | female | 451.90(317.99,610.56) | 950.93(672.43,1312.05) | 2.57(2.31,2.84) | 53.76(48.91,58.95) | 116.77(109.45,124.45) | 2.59(2.46,2.73) |
| Turkey | female | 2888.04(1989.85,4048.11) | 5283.26(3736.95,7182.93) | 2.91(2.56,3.25) | 49.80(48.00,51.65) | 80.72(78.55,82.92) | 2.66(2.24,3.08) |
| Turkmenistan | female | 237.12(168.06,322.11) | 543.01(403.51,704.14) | 3.39(3.05,3.73) | 66.60(58.39,75.64) | 141.40(129.75,153.82) | 2.87(2.75,2.99) |
| Tuvalu | female | 1.44(1.12,1.84) | 3.20(2.35,4.06) | 3.03(2.83,3.22) | 178.22(11.78,796.44) | 306.89(67.67,871.59) | 1.83(1.77,1.89) |
| Uganda | female | 964.02(662.99,1303.41) | 3501.24(2473.46,4722.34) | 4.38(4.18,4.59) | 53.99(50.63,57.52) | 79.72(77.10,82.42) | 1.16(1.01,1.30) |
| Ukraine | female | 2443.72(1746.38,3252.03) | 1474.00(1007.40,2044.74) | -2.04(-2.72,-1.37) | 69.75(67.01,72.57) | 68.51(65.05,72.11) | -0.61(-0.89,-0.32) |
| United Arab Emirates | female | 77.72(53.68,108.22) | 415.33(292.34,568.62) | 5.75(4.60,6.91) | 67.88(53.61,84.90) | 132.48(120.04,145.86) | 2.33(2.20,2.46) |
| United Kingdom | female | 2432.47(1478.14,3592.08) | 8216.82(5795.64,11109.78) | 7.93(6.55,9.33) | 57.77(55.48,60.12) | 221.27(216.49,226.12) | 7.71(6.41,9.03) |
| United Republic of Tanzania | female | 865.24(560.15,1239.99) | 3339.06(2369.98,4582.21) | 4.51(4.32,4.71) | 32.15(30.04,34.37) | 57.22(55.30,59.20) | 1.93(1.79,2.07) |
| United States of America | female | 7878.39(4070.79,12300.50) | 16270.73(10383.50,22919.24) | 3.06(2.80,3.33) | 42.32(41.39,43.27) | 77.01(75.83,78.20) | 2.35(2.15,2.55) |
| United States Virgin Islands | female | 8.96(6.77,11.69) | 10.61(7.94,13.93) | 0.51(0.41,0.62) | 102.72(46.73,196.98) | 174.06(85.59,314.95) | 1.67(1.53,1.80) |
| Uruguay | female | 37.43(16.86,60.91) | 111.24(68.88,160.33) | 3.94(3.64,4.25) | 15.45(10.90,21.26) | 43.57(35.85,52.51) | 3.85(3.49,4.20) |
| Uzbekistan | female | 1110.23(791.56,1505.91) | 3069.52(2272.79,3925.76) | 3.95(3.45,4.45) | 55.57(52.35,58.94) | 105.80(102.08,109.61) | 2.24(1.98,2.51) |
| Vanuatu | female | 20.90(15.88,26.27) | 74.27(56.48,96.72) | 4.73(4.60,4.85) | 147.49(91.15,225.97) | 268.05(210.58,336.41) | 2.20(2.17,2.23) |
| Venezuela (Bolivarian Republic of) | female | 1630.62(1222.26,2146.27) | 3935.68(3057.10,4951.53) | 3.49(3.11,3.87) | 86.62(82.47,90.93) | 181.44(175.82,187.20) | 2.60(2.16,3.05) |
| Viet Nam | female | 2954.73(2148.93,4006.85) | 4619.99(3504.77,6056.49) | 1.86(1.62,2.10) | 42.78(41.25,44.35) | 66.14(64.24,68.08) | 1.41(1.11,1.70) |
| Yemen | female | 469.14(323.77,650.00) | 2360.63(1667.89,3254.88) | 5.14(4.56,5.72) | 42.84(39.03,46.91) | 81.31(78.06,84.67) | 1.66(0.95,2.38) |
| Zambia | female | 533.74(378.12,728.86) | 1315.94(931.51,1794.36) | 2.79(2.64,2.95) | 60.89(55.82,66.30) | 66.92(63.35,70.64) | -0.09(-0.26,0.09) |
| Zimbabwe | female | 616.28(423.91,850.29) | 1596.80(1149.52,2192.20) | 3.14(2.65,3.63) | 56.30(51.93,60.94) | 101.39(96.48,106.50) | 2.21(1.87,2.56) |
| Afghanistan | male | 702.73(483.72,964.99) | 5334.74(3778.05,7240.01) | 6.30(5.56,7.04) | 62.45(57.87,67.31) | 130.93(127.44,134.50) | 2.54(2.52,2.56) |
| Albania | male | 108.74(69.97,154.40) | 121.16(77.54,171.39) | 0.51(0.17,0.85) | 33.74(27.69,40.71) | 53.91(44.71,64.51) | 1.45(1.28,1.62) |
| Algeria | male | 1645.86(1159.70,2274.19) | 3954.10(2813.18,5480.97) | 3.07(2.47,3.68) | 62.72(59.72,65.82) | 120.83(117.09,124.67) | 2.13(1.92,2.35) |
| American Samoa | male | 19.83(14.78,25.58) | 38.39(28.99,50.23) | 1.88(1.56,2.20) | 407.09(248.06,630.15) | 699.65(495.60,960.74) | 1.82(1.52,2.13) |
| Andorra | male | 1.66(0.89,2.52) | 4.08(2.57,6.00) | 2.63(2.24,3.01) | 35.79(3.08,152.93) | 90.20(24.93,232.74) | 3.23(3.20,3.26) |
| Angola | male | 828.70(600.98,1098.30) | 3661.71(2740.00,4750.61) | 5.34(5.18,5.51) | 85.37(79.64,91.40) | 136.04(131.66,140.54) | 1.74(1.60,1.87) |
| Antigua and Barbuda | male | 5.68(4.14,7.39) | 11.66(8.54,15.35) | 2.58(2.51,2.66) | 99.59(35.29,222.11) | 161.33(82.36,285.56) | 1.55(1.47,1.62) |
| Argentina | male | 732.97(514.58,993.20) | 2564.79(1771.69,3511.14) | 4.91(4.72,5.10) | 27.68(25.71,29.76) | 70.45(67.75,73.23) | 3.84(3.60,4.08) |
| Armenia | male | 165.17(113.05,226.53) | 165.76(112.14,228.59) | 0.51(-0.08,1.10) | 57.92(49.43,67.47) | 86.66(73.94,101.01) | 1.39(1.26,1.52) |
| Australia | male | 216.68(103.49,347.09) | 503.97(224.32,840.13) | 2.36(1.91,2.81) | 15.70(13.68,17.94) | 31.51(28.82,34.40) | 1.67(1.30,2.04) |
| Austria | male | 178.10(107.11,265.62) | 336.49(194.74,500.84) | 2.66(2.44,2.88) | 27.73(23.77,32.20) | 62.25(55.75,69.34) | 2.78(2.63,2.93) |
| Azerbaijan | male | 449.61(309.52,609.67) | 739.57(525.73,1005.37) | 2.48(2.10,2.87) | 63.95(58.17,70.14) | 94.04(87.36,101.12) | 1.25(1.17,1.33) |
| Bahamas | male | 23.31(17.22,31.16) | 53.18(39.60,69.98) | 3.13(2.99,3.27) | 86.72(55.15,129.89) | 171.79(128.73,224.83) | 2.43(2.35,2.51) |
| Bahrain | male | 32.69(22.90,45.76) | 137.21(98.48,179.54) | 5.93(4.95,6.93) | 69.28(47.28,98.84) | 160.19(134.51,189.35) | 2.90(2.81,2.98) |
| Bangladesh | male | 6839.11(4917.31,9188.01) | 13540.82(9788.52,18354.76) | 1.99(1.80,2.18) | 65.17(63.63,66.74) | 95.19(93.59,96.81) | 0.88(0.75,1.00) |
| Barbados | male | 22.83(17.17,29.48) | 28.02(20.68,37.17) | 0.63(0.43,0.82) | 98.49(62.30,148.31) | 142.46(94.68,205.88) | 1.06(0.98,1.15) |
| Belarus | male | 314.81(202.38,436.29) | 218.57(128.71,328.27) | -0.99(-1.71,-0.27) | 43.31(38.66,48.37) | 45.23(39.43,51.66) | -0.05(-0.24,0.15) |
| Belgium | male | 287.88(180.17,416.63) | 588.69(372.79,868.18) | 2.71(2.49,2.92) | 39.14(34.73,43.96) | 87.90(80.93,95.32) | 2.75(2.66,2.83) |
| Belize | male | 11.61(8.55,14.93) | 47.16(35.06,60.93) | 5.02(4.89,5.16) | 62.00(31.58,109.84) | 113.92(83.73,151.51) | 2.06(1.92,2.20) |
| Benin | male | 135.57(89.17,193.37) | 813.05(556.75,1123.93) | 6.59(6.18,7.00) | 35.68(29.91,42.28) | 68.02(63.42,72.88) | 2.41(2.01,2.81) |
| Bermuda | male | 2.74(2.00,3.67) | 2.86(2.10,3.83) | 0.29(0.15,0.44) | 60.53(11.12,192.93) | 93.81(18.34,281.46) | 1.45(1.39,1.51) |
| Bhutan | male | 47.01(33.52,64.02) | 81.00(57.40,108.13) | 2.83(2.53,3.13) | 62.63(46.02,83.32) | 103.23(81.89,128.62) | 1.86(1.79,1.92) |
| Bolivia (Plurinational State of) | male | 242.18(170.31,330.34) | 702.31(501.53,938.05) | 3.83(3.55,4.11) | 41.17(36.13,46.72) | 64.83(60.12,69.81) | 1.50(1.41,1.59) |
| Bosnia and Herzegovina | male | 211.80(142.42,292.92) | 187.89(123.93,263.84) | 0.41(-0.20,1.02) | 49.84(43.35,57.05) | 86.45(74.50,99.86) | 2.05(1.93,2.17) |
| Botswana | male | 69.32(49.58,94.22) | 155.90(110.97,210.15) | 2.53(2.02,3.04) | 55.26(42.97,70.05) | 74.17(62.98,86.77) | 0.81(0.59,1.02) |
| Brazil | male | 9373.96(6646.48,12876.77) | 15427.58(10879.89,21054.56) | 1.70(1.56,1.84) | 65.01(63.70,66.34) | 88.93(87.54,90.35) | 1.07(0.96,1.17) |
| Brunei Darussalam | male | 42.39(30.53,56.38) | 66.39(46.25,92.44) | 1.36(1.28,1.44) | 159.98(115.43,216.44) | 160.97(124.52,205.04) | -0.41(-0.58,-0.24) |
| Bulgaria | male | 348.19(239.05,491.37) | 252.44(169.91,352.22) | -1.72(-2.01,-1.43) | 57.01(51.18,63.33) | 78.35(68.98,88.64) | 0.53(0.28,0.78) |
| Burkina Faso | male | 285.93(188.00,405.24) | 1348.08(947.11,1813.34) | 5.46(5.42,5.50) | 37.20(32.97,41.82) | 65.59(62.12,69.21) | 1.93(1.88,1.98) |
| Burundi | male | 318.18(226.73,419.66) | 795.53(578.45,1060.72) | 3.60(3.40,3.81) | 66.35(59.25,74.08) | 73.55(68.51,78.87) | 0.22(0.15,0.28) |
| Cabo Verde | male | 13.02(8.41,18.84) | 37.97(25.84,53.50) | 4.47(3.86,5.07) | 38.04(20.25,65.22) | 72.89(51.55,100.24) | 2.42(2.24,2.60) |
| Cambodia | male | 423.53(319.29,554.15) | 1432.81(1044.43,1909.17) | 4.74(4.51,4.97) | 46.00(41.72,50.60) | 94.98(90.13,100.04) | 2.48(2.32,2.64) |
| Cameroon | male | 291.70(184.23,412.53) | 1789.79(1235.59,2447.99) | 7.21(6.05,8.38) | 32.31(28.69,36.27) | 61.79(58.95,64.73) | 2.96(1.77,4.16) |
| Canada | male | 96.95(20.90,201.00) | 404.04(94.35,812.54) | 4.30(3.80,4.80) | 4.87(3.95,5.95) | 18.65(16.87,20.57) | 3.69(3.08,4.30) |
| Central African Republic | male | 246.09(174.12,324.82) | 807.37(597.71,1049.53) | 4.07(3.81,4.33) | 96.74(85.02,109.64) | 154.80(144.29,165.89) | 1.67(1.57,1.77) |
| Chad | male | 176.99(116.75,250.60) | 894.77(612.72,1214.28) | 5.63(5.58,5.67) | 34.86(29.90,40.44) | 60.08(56.18,64.19) | 1.86(1.75,1.97) |
| Chile | male | 407.33(270.37,567.04) | 903.91(614.31,1269.87) | 2.98(2.84,3.12) | 31.99(28.96,35.26) | 66.16(61.91,70.64) | 2.38(2.28,2.48) |
| China | male | 139938.28(99866.61,190298.30) | 140924.11(102384.34,189653.58) | 3.02(2.01,4.04) | 103.72(103.18,104.26) | 166.20(165.34,167.08) | 3.98(3.28,4.68) |
| Colombia | male | 2993.81(2174.56,3968.43) | 6098.63(4517.50,7998.11) | 2.35(2.20,2.51) | 93.28(89.97,96.68) | 147.12(143.45,150.86) | 1.35(1.25,1.45) |
| Comoros | male | 26.39(19.17,34.95) | 49.59(35.56,65.80) | 2.10(2.02,2.17) | 58.05(37.98,85.23) | 71.79(53.21,94.78) | 0.70(0.63,0.76) |
| Congo | male | 244.50(173.93,322.98) | 566.09(412.24,754.42) | 2.52(2.37,2.66) | 100.63(88.38,114.12) | 123.11(113.16,133.71) | 0.53(0.45,0.60) |
| Cook Islands | male | 3.44(2.65,4.42) | 4.71(3.61,6.08) | 0.96(0.73,1.19) | 182.93(43.28,507.81) | 374.42(116.30,897.18) | 2.51(2.44,2.58) |
| Costa Rica | male | 239.08(177.47,315.94) | 631.33(475.53,831.82) | 3.58(3.20,3.97) | 82.10(72.02,93.20) | 165.90(153.19,179.42) | 2.43(2.38,2.47) |
| Croatia | male | 197.77(136.14,272.62) | 204.73(133.11,297.85) | -0.19(-0.36,-0.01) | 55.78(48.28,64.14) | 81.76(70.90,93.89) | 1.05(0.95,1.15) |
| Cuba | male | 1482.50(1057.41,1977.40) | 1243.87(894.84,1701.53) | -0.81(-1.22,-0.40) | 125.35(119.04,131.90) | 170.56(161.20,180.33) | 0.48(0.22,0.74) |
| Cyprus | male | 24.41(15.42,35.23) | 65.91(41.66,100.49) | 4.11(3.57,4.65) | 36.96(23.77,55.00) | 80.53(62.11,103.06) | 2.73(2.57,2.89) |
| Czechia | male | 526.84(360.58,742.21) | 586.37(379.29,840.26) | -0.07(-0.50,0.36) | 70.84(64.90,77.18) | 119.00(109.56,129.05) | 1.65(1.56,1.73) |
| Côte d'Ivoire | male | 412.32(260.43,596.25) | 1534.41(1074.23,2093.75) | 4.11(3.68,4.54) | 36.06(32.66,39.71) | 60.87(57.86,64.00) | 1.63(1.49,1.77) |
| Democratic People's Republic of Korea | male | 1951.50(1509.15,2528.74) | 3314.22(2541.13,4297.93) | 2.32(2.09,2.55) | 114.40(109.37,119.59) | 165.13(159.53,170.88) | 1.09(1.00,1.17) |
| Democratic Republic of the Congo | male | 2757.04(2007.72,3649.83) | 10738.81(7799.05,14303.67) | 4.75(4.64,4.87) | 75.99(73.18,78.89) | 121.99(119.69,124.33) | 1.49(1.37,1.61) |
| Denmark | male | 151.81(89.99,224.69) | 311.08(200.01,437.45) | 3.05(2.60,3.49) | 37.41(31.68,43.90) | 86.97(77.55,97.23) | 3.24(3.11,3.37) |
| Djibouti | male | 33.34(23.75,44.89) | 94.19(69.20,122.64) | 4.20(3.97,4.44) | 55.91(38.56,78.38) | 82.65(66.81,101.13) | 1.40(1.35,1.45) |
| Dominica | male | 8.30(6.14,11.00) | 13.73(10.20,17.74) | 2.03(1.87,2.19) | 110.91(48.72,216.92) | 241.14(130.92,406.84) | 2.71(2.51,2.92) |
| Dominican Republic | male | 381.34(283.65,489.19) | 1034.48(782.25,1352.06) | 3.91(3.75,4.07) | 51.51(46.47,56.95) | 104.82(98.53,111.42) | 2.68(2.57,2.80) |
| Ecuador | male | 407.27(284.86,586.32) | 1307.15(924.80,1760.95) | 3.48(3.17,3.79) | 41.21(37.30,45.42) | 79.10(74.87,83.51) | 1.74(1.45,2.03) |
| Egypt | male | 2110.51(1414.84,2904.43) | 11169.45(8003.62,14722.98) | 6.29(6.11,6.46) | 39.11(37.46,40.82) | 121.25(119.01,123.52) | 4.22(3.95,4.50) |
| El Salvador | male | 472.35(356.21,608.41) | 1004.96(745.20,1320.65) | 3.06(2.75,3.37) | 91.06(83.00,99.70) | 168.18(157.93,178.92) | 2.47(2.11,2.84) |
| Equatorial Guinea | male | 34.02(24.20,45.58) | 249.12(185.99,324.33) | 7.39(7.26,7.53) | 94.38(65.25,132.37) | 126.78(111.51,143.59) | 0.94(0.77,1.11) |
| Eritrea | male | 209.97(150.93,275.30) | 654.00(473.76,860.85) | 4.01(3.72,4.29) | 69.44(60.34,79.55) | 90.34(83.54,97.54) | 0.85(0.70,1.00) |
| Estonia | male | 59.28(39.41,83.84) | 41.70(26.13,61.03) | -0.59(-1.22,0.05) | 52.96(40.34,68.28) | 63.06(45.38,85.51) | 0.73(0.47,1.00) |
| Eswatini | male | 37.80(25.91,51.43) | 105.19(75.56,137.78) | 3.43(2.69,4.19) | 50.59(35.66,69.85) | 90.65(74.15,109.76) | 2.06(1.55,2.56) |
| Ethiopia | male | 3011.35(2173.52,3950.78) | 6343.97(4455.96,8427.96) | 2.43(2.29,2.57) | 64.48(62.19,66.84) | 54.81(53.47,56.18) | -0.60(-0.72,-0.47) |
| Fiji | male | 157.90(120.88,199.89) | 300.90(236.08,380.15) | 2.18(1.93,2.43) | 211.08(179.43,246.72) | 388.45(345.79,434.94) | 2.06(2.01,2.12) |
| Finland | male | 216.93(121.84,332.28) | 500.89(338.08,685.77) | 2.79(2.41,3.17) | 62.80(54.69,71.81) | 156.87(143.41,171.27) | 2.75(2.30,3.21) |
| France | male | 960.75(598.31,1392.81) | 1939.99(1083.63,2906.57) | 1.44(0.22,2.67) | 21.36(20.03,22.76) | 48.12(46.00,50.31) | 1.77(0.56,3.00) |
| Gabon | male | 92.67(66.44,122.33) | 236.33(176.90,309.69) | 3.35(3.23,3.48) | 101.44(81.84,124.37) | 144.67(126.81,164.34) | 1.21(1.15,1.28) |
| Gambia | male | 26.80(16.86,38.34) | 129.26(87.35,178.85) | 5.34(5.24,5.45) | 29.25(19.24,42.72) | 56.51(47.17,67.18) | 2.32(2.26,2.37) |
| Georgia | male | 295.13(207.94,401.01) | 268.54(188.46,378.07) | 0.19(-0.28,0.66) | 69.04(61.38,77.39) | 121.72(107.58,137.25) | 2.32(2.04,2.60) |
| Germany | male | 3666.43(2483.54,5095.49) | 6746.55(4639.05,9385.96) | 1.89(1.65,2.13) | 62.79(60.73,64.90) | 136.00(132.76,139.31) | 2.16(1.93,2.39) |
| Ghana | male | 465.04(284.14,677.93) | 2244.19(1569.96,3075.04) | 5.53(5.30,5.77) | 33.70(30.70,36.92) | 71.97(69.02,75.01) | 2.48(2.22,2.74) |
| Greece | male | 308.42(194.20,454.39) | 536.56(346.04,773.10) | 1.30(0.82,1.79) | 38.21(34.06,42.73) | 102.66(94.15,111.73) | 3.23(3.05,3.41) |
| Greenland | male | 0.43(0.08,1.02) | 1.00(0.32,1.82) | 4.81(3.99,5.63) | 7.90(0.00,111.01) | 25.63(0.61,152.15) | 4.69(4.22,5.15) |
| Grenada | male | 7.84(5.73,10.51) | 15.92(11.36,21.34) | 2.32(2.02,2.62) | 97.24(41.50,193.87) | 162.87(92.77,266.77) | 1.78(1.72,1.85) |
| Guam | male | 29.41(22.88,37.64) | 50.05(39.33,63.55) | 2.31(1.95,2.67) | 186.92(124.94,270.69) | 339.78(252.02,449.05) | 2.17(2.06,2.28) |
| Guatemala | male | 620.14(469.31,800.03) | 3126.66(2326.08,4010.55) | 6.16(5.75,6.58) | 88.23(81.40,95.49) | 164.65(158.93,170.52) | 2.31(2.02,2.60) |
| Guinea | male | 130.34(80.32,190.70) | 634.13(446.24,876.02) | 5.31(5.10,5.51) | 26.36(22.01,31.33) | 56.47(52.14,61.07) | 2.54(2.40,2.68) |
| Guinea-Bissau | male | 34.00(22.40,48.87) | 122.52(85.46,169.62) | 4.45(4.29,4.60) | 37.39(25.84,52.45) | 65.08(54.05,77.71) | 1.83(1.77,1.89) |
| Guyana | male | 100.37(72.66,132.84) | 165.89(118.20,217.11) | 1.54(1.23,1.85) | 122.82(99.97,149.36) | 208.88(178.22,243.43) | 1.69(1.62,1.77) |
| Haiti | male | 498.38(360.60,656.13) | 1551.07(1153.76,2059.18) | 3.98(3.83,4.12) | 90.42(82.64,98.75) | 135.67(129.00,142.60) | 1.29(1.17,1.41) |
| Honduras | male | 538.55(399.10,717.24) | 1973.11(1486.21,2620.19) | 4.55(4.31,4.79) | 127.76(117.11,139.14) | 195.47(186.94,204.29) | 1.49(1.37,1.61) |
| Hungary | male | 484.48(334.59,687.31) | 461.63(304.87,658.15) | -0.58(-0.91,-0.26) | 66.33(60.55,72.51) | 86.47(78.75,94.77) | 0.84(0.72,0.96) |
| Iceland | male | 7.90(4.60,11.83) | 23.35(15.30,32.81) | 4.21(4.09,4.33) | 36.21(15.53,71.97) | 97.25(61.76,146.53) | 3.55(3.46,3.63) |
| India | male | 61816.04(42364.43,85134.56) | 182991.57(128719.31,251683.87) | 3.55(3.37,3.74) | 74.18(73.60,74.77) | 133.76(133.15,134.37) | 1.61(1.36,1.87) |
| Indonesia | male | 9070.58(6705.21,11891.75) | 17025.48(12466.54,22345.36) | 1.04(-0.02,2.12) | 50.40(49.37,51.45) | 75.60(74.47,76.75) | 0.49(-0.55,1.54) |
| Iran (Islamic Republic of) | male | 3039.31(1996.43,4289.72) | 5634.52(3790.73,7938.24) | 2.72(1.63,3.82) | 52.01(50.17,53.89) | 95.90(93.41,98.44) | 2.23(1.81,2.65) |
| Iraq | male | 1610.88(1159.92,2184.93) | 6988.56(5080.27,9562.10) | 4.81(4.51,5.11) | 96.26(91.60,101.11) | 154.20(150.61,157.86) | 1.57(1.35,1.80) |
| Ireland | male | 85.65(41.61,135.98) | 240.66(148.73,347.32) | 3.28(3.07,3.49) | 27.80(22.21,34.40) | 78.52(68.91,89.10) | 3.55(3.47,3.64) |
| Israel | male | 149.56(95.50,213.76) | 562.48(387.35,769.87) | 3.19(2.23,4.16) | 34.69(29.34,40.74) | 79.94(73.46,86.83) | 1.75(0.75,2.76) |
| Italy | male | 2316.62(1233.84,3582.85) | 2602.72(1494.66,3930.97) | -0.81(-2.14,0.55) | 49.82(47.81,51.89) | 84.51(81.29,87.83) | 0.61(-0.58,1.82) |
| Jamaica | male | 168.89(124.42,226.43) | 351.79(256.11,468.42) | 2.63(2.31,2.95) | 70.51(60.27,82.00) | 132.44(118.93,147.11) | 1.95(1.65,2.24) |
| Japan | male | 4942.81(3410.54,6831.22) | 5236.80(3617.37,7262.84) | -0.54(-0.85,-0.22) | 50.67(49.26,52.10) | 82.90(80.67,85.18) | 1.45(1.29,1.62) |
| Jordan | male | 324.80(233.26,434.78) | 1840.92(1316.77,2505.11) | 6.59(6.15,7.04) | 70.03(62.62,78.08) | 150.63(143.82,157.67) | 3.02(2.65,3.38) |
| Kazakhstan | male | 966.73(674.71,1329.51) | 1172.82(798.32,1626.19) | 0.92(0.53,1.30) | 68.88(64.61,73.37) | 93.39(88.12,98.90) | 0.71(0.47,0.95) |
| Kenya | male | 959.68(654.51,1309.10) | 3643.42(2616.80,4843.19) | 4.66(4.39,4.93) | 42.37(39.72,45.16) | 68.01(65.81,70.26) | 1.74(1.57,1.91) |
| Kiribati | male | 20.08(15.22,24.93) | 54.92(42.43,70.87) | 4.26(3.87,4.65) | 275.47(168.41,425.90) | 511.53(385.27,665.96) | 2.19(1.96,2.43) |
| Kuwait | male | 177.30(121.96,246.62) | 644.87(460.68,872.03) | 4.69(4.38,5.00) | 102.36(87.58,119.13) | 220.56(203.72,238.51) | 1.93(1.68,2.17) |
| Kyrgyzstan | male | 211.03(144.23,287.11) | 426.03(291.90,589.08) | 2.76(2.50,3.02) | 52.37(45.54,59.96) | 74.78(67.83,82.26) | 1.14(1.09,1.18) |
| Lao People's Democratic Republic | male | 266.89(197.93,340.63) | 739.77(553.39,981.23) | 3.51(3.28,3.74) | 74.95(66.19,84.57) | 105.12(97.68,112.97) | 0.91(0.83,0.99) |
| Latvia | male | 101.65(64.33,140.60) | 52.53(32.15,75.96) | -1.60(-2.27,-0.91) | 52.98(43.18,64.37) | 57.77(43.21,75.70) | 0.34(0.14,0.54) |
| Lebanon | male | 211.02(147.20,293.73) | 512.38(359.65,707.43) | 3.87(3.52,4.22) | 69.84(60.72,79.97) | 136.33(124.77,148.69) | 2.44(2.34,2.53) |
| Lesotho | male | 73.98(49.11,101.91) | 179.54(129.96,236.44) | 3.12(2.78,3.46) | 43.24(33.91,54.40) | 83.52(71.75,96.67) | 2.51(2.36,2.65) |
| Liberia | male | 59.10(38.54,86.06) | 379.04(255.52,528.40) | 6.20(5.70,6.71) | 43.23(32.60,56.43) | 79.46(71.65,87.91) | 2.08(2.04,2.11) |
| Libya | male | 345.24(245.62,476.58) | 953.03(670.87,1318.73) | 3.70(3.24,4.16) | 79.11(70.97,87.94) | 156.68(146.89,166.97) | 2.51(2.41,2.61) |
| Lithuania | male | 105.95(63.59,153.31) | 87.49(51.54,133.90) | -0.12(-0.61,0.38) | 36.40(29.80,44.05) | 52.09(41.72,64.41) | 1.40(1.04,1.76) |
| Luxembourg | male | 9.46(5.79,13.91) | 33.28(20.76,47.91) | 5.11(4.76,5.45) | 34.94(16.20,67.03) | 86.72(59.70,122.28) | 3.56(3.34,3.78) |
| Madagascar | male | 641.03(450.75,852.68) | 2021.31(1462.19,2686.34) | 3.94(3.85,4.03) | 57.13(52.77,61.75) | 74.22(71.01,77.53) | 0.83(0.77,0.89) |
| Malawi | male | 598.21(432.26,800.06) | 2063.10(1514.97,2722.24) | 4.04(3.86,4.22) | 67.23(61.94,72.85) | 107.90(103.26,112.71) | 1.48(1.28,1.67) |
| Malaysia | male | 1183.99(868.78,1567.84) | 3527.68(2570.25,4731.60) | 3.83(3.53,4.13) | 70.43(66.48,74.56) | 119.76(115.84,123.79) | 1.58(1.46,1.71) |
| Maldives | male | 10.13(7.37,13.45) | 36.96(26.99,49.56) | 5.50(5.18,5.82) | 48.06(23.15,88.42) | 85.76(59.83,120.16) | 2.00(1.87,2.13) |
| Mali | male | 204.06(125.98,298.71) | 1116.82(755.72,1525.30) | 5.99(5.84,6.15) | 29.21(25.32,33.53) | 52.36(49.32,55.54) | 2.02(1.98,2.06) |
| Malta | male | 15.52(9.30,23.03) | 23.01(14.68,32.85) | 1.48(0.87,2.10) | 59.09(33.42,96.91) | 93.57(59.09,142.02) | 1.65(1.33,1.96) |
| Marshall Islands | male | 16.56(12.51,21.43) | 37.34(28.10,48.30) | 2.62(2.09,3.15) | 392.10(225.81,635.84) | 721.37(508.69,993.60) | 2.13(1.98,2.29) |
| Mauritania | male | 40.77(23.04,62.25) | 132.76(87.78,187.13) | 3.90(3.75,4.05) | 21.86(15.66,29.74) | 34.05(28.49,40.40) | 1.31(1.18,1.44) |
| Mauritius | male | 99.46(72.38,131.77) | 152.08(110.03,202.09) | 2.65(2.28,3.02) | 91.97(74.77,112.04) | 159.10(134.81,186.55) | 3.04(2.62,3.47) |
| Mexico | male | 11535.46(8658.41,14880.37) | 19778.46(14679.05,25718.20) | 2.18(1.95,2.41) | 131.03(128.64,133.44) | 182.71(180.17,185.28) | 1.53(1.30,1.76) |
| Micronesia (Federated States of) | male | 17.37(13.42,21.81) | 41.22(32.00,52.81) | 2.84(1.98,3.70) | 182.04(106.04,293.23) | 393.12(282.28,533.44) | 2.74(2.01,3.48) |
| Monaco | male | 0.61(0.32,0.94) | 1.76(1.11,2.52) | 4.33(4.01,4.66) | 35.92(0.10,342.72) | 95.98(9.39,373.71) | 3.52(3.46,3.59) |
| Mongolia | male | 74.48(48.64,104.03) | 132.24(86.54,181.48) | 2.65(1.98,3.33) | 34.14(26.82,42.86) | 54.49(45.58,64.70) | 1.89(1.62,2.16) |
| Montenegro | male | 34.88(23.87,48.63) | 38.40(25.27,54.29) | 0.17(0.01,0.34) | 63.80(44.40,88.89) | 92.36(65.48,126.61) | 1.14(1.06,1.22) |
| Morocco | male | 1418.48(976.64,1992.59) | 3795.86(2702.61,5231.63) | 3.56(3.41,3.72) | 56.80(53.88,59.84) | 123.09(119.21,127.07) | 2.76(2.75,2.78) |
| Mozambique | male | 644.75(462.69,851.64) | 3097.77(2293.75,3994.90) | 5.54(5.31,5.76) | 62.27(57.50,67.35) | 113.15(109.19,117.23) | 2.36(2.19,2.53) |
| Myanmar | male | 2512.28(1871.74,3314.36) | 5040.13(3808.93,6461.45) | 2.42(2.39,2.44) | 62.79(60.36,65.30) | 106.33(103.42,109.31) | 1.87(1.83,1.91) |
| Namibia | male | 66.50(45.09,91.97) | 151.56(105.20,207.77) | 2.72(2.40,3.05) | 46.54(36.01,59.24) | 65.30(55.32,76.57) | 1.03(0.89,1.17) |
| Nauru | male | 1.87(1.44,2.36) | 4.13(3.22,5.20) | 2.66(2.60,2.71) | 200.09(21.70,756.90) | 386.08(108.04,980.73) | 2.31(2.23,2.39) |
| Nepal | male | 1127.87(777.58,1601.98) | 4035.72(2856.00,5516.52) | 4.17(3.88,4.46) | 66.02(62.22,70.01) | 133.45(129.36,137.64) | 2.22(1.93,2.51) |
| Netherlands | male | 438.82(259.59,672.73) | 859.35(542.58,1247.88) | 3.14(2.51,3.78) | 34.91(31.71,38.36) | 79.59(74.35,85.10) | 3.43(3.09,3.77) |
| New Zealand | male | 48.62(15.64,91.08) | 115.00(54.95,185.71) | 1.94(1.03,2.86) | 16.73(12.36,22.14) | 38.63(31.89,46.38) | 1.53(0.64,2.43) |
| Nicaragua | male | 360.15(267.42,474.92) | 1010.31(765.38,1329.21) | 3.68(3.34,4.02) | 97.60(87.74,108.28) | 160.03(150.32,170.22) | 1.79(1.69,1.89) |
| Niger | male | 105.56(56.19,161.50) | 815.80(544.01,1151.23) | 7.07(6.51,7.62) | 16.01(13.10,19.40) | 38.04(35.46,40.76) | 2.94(2.54,3.34) |
| Nigeria | male | 2370.13(1492.34,3404.68) | 7942.62(5127.23,11253.29) | 4.48(4.23,4.73) | 29.26(28.10,30.47) | 39.16(38.30,40.04) | 1.30(1.07,1.53) |
| Niue | male | 0.62(0.47,0.79) | 0.80(0.61,1.02) | 1.02(0.77,1.28) | 300.99(1.01,2487.81) | 629.89(7.09,4266.80) | 3.05(2.89,3.21) |
| North Macedonia | male | 88.61(56.53,128.08) | 116.24(76.49,169.36) | 1.14(0.81,1.47) | 50.27(40.35,61.91) | 78.81(65.08,94.74) | 1.47(1.37,1.57) |
| Northern Mariana Islands | male | 8.79(6.77,11.23) | 14.24(11.04,18.52) | 1.81(1.37,2.24) | 205.72(92.38,401.77) | 366.75(201.23,616.19) | 2.02(1.93,2.11) |
| Norway | male | 265.43(162.15,383.03) | 392.49(262.28,557.13) | 0.09(-0.26,0.45) | 78.01(68.89,88.03) | 112.85(101.94,124.63) | -0.28(-0.83,0.27) |
| Oman | male | 99.28(68.51,138.77) | 517.94(355.94,724.74) | 5.99(5.45,6.53) | 53.05(43.11,64.65) | 111.38(101.37,122.27) | 2.21(1.75,2.68) |
| Pakistan | male | 9814.07(6927.09,13610.06) | 31109.20(22320.32,42061.90) | 3.71(3.32,4.09) | 88.27(86.53,90.04) | 134.04(132.55,135.54) | 1.15(0.83,1.48) |
| Palau | male | 3.78(2.88,4.83) | 5.95(4.51,7.59) | 1.71(1.28,2.13) | 229.84(59.61,604.89) | 453.46(165.22,1003.65) | 2.42(2.34,2.51) |
| Palestine | male | 132.22(91.14,184.18) | 578.43(408.60,791.21) | 5.44(5.32,5.56) | 63.39(53.03,75.20) | 110.68(101.84,120.08) | 1.83(1.77,1.90) |
| Panama | male | 205.53(154.05,269.53) | 534.80(400.23,706.55) | 3.39(3.33,3.44) | 83.98(72.89,96.29) | 148.18(135.89,161.31) | 2.11(2.01,2.21) |
| Papua New Guinea | male | 1197.56(938.75,1488.63) | 4472.61(3511.58,5579.47) | 4.73(4.59,4.86) | 287.09(271.05,303.84) | 453.71(440.51,467.21) | 1.49(1.34,1.63) |
| Paraguay | male | 187.88(137.07,252.85) | 500.77(372.71,664.12) | 3.29(2.98,3.60) | 50.84(43.82,58.67) | 76.03(69.51,82.99) | 1.05(0.82,1.28) |
| Peru | male | 597.55(398.56,835.89) | 1614.04(1150.84,2258.53) | 3.31(3.15,3.47) | 27.56(25.40,29.87) | 53.71(51.12,56.40) | 2.20(2.06,2.35) |
| Philippines | male | 4645.04(3526.11,5995.64) | 6835.10(5048.12,9097.78) | 0.77(0.59,0.94) | 73.10(71.01,75.23) | 64.04(62.53,65.58) | -1.09(-1.30,-0.88) |
| Poland | male | 1494.20(984.17,2180.55) | 1859.12(1202.81,2678.63) | 1.05(0.68,1.41) | 56.68(53.83,59.63) | 88.52(84.53,92.66) | 1.89(1.30,2.48) |
| Portugal | male | 464.82(299.76,657.66) | 685.33(443.29,994.36) | 0.53(0.19,0.86) | 55.89(50.93,61.22) | 116.76(108.18,125.84) | 2.23(2.05,2.42) |
| Puerto Rico | male | 289.37(194.38,398.94) | 408.77(293.48,568.75) | 1.27(0.83,1.71) | 93.14(82.71,104.55) | 175.53(158.93,193.41) | 2.22(1.90,2.54) |
| Qatar | male | 37.07(25.08,51.94) | 561.30(367.56,805.56) | 12.74(11.40,14.11) | 81.50(56.66,114.84) | 144.06(130.14,159.56) | 2.61(2.28,2.95) |
| Republic of Korea | male | 2347.17(1647.74,3288.85) | 3033.63(2154.89,4110.18) | 1.37(0.85,1.89) | 49.65(47.66,51.71) | 91.73(88.46,95.09) | 2.52(1.90,3.15) |
| Republic of Moldova | male | 182.67(119.93,257.67) | 141.73(89.86,212.96) | -0.33(-0.95,0.30) | 58.72(50.50,67.90) | 65.59(55.23,77.39) | 0.66(0.49,0.84) |
| Romania | male | 937.24(629.49,1315.01) | 662.78(413.69,971.49) | -1.81(-2.11,-1.51) | 47.43(44.44,50.57) | 63.89(59.12,68.95) | 0.76(0.65,0.86) |
| Russian Federation | male | 4575.19(3012.11,6352.87) | 3534.13(2219.80,5097.17) | -0.95(-1.59,-0.31) | 44.04(42.77,45.34) | 47.61(46.05,49.21) | 0.10(-0.03,0.23) |
| Rwanda | male | 421.88(297.86,571.29) | 853.65(620.37,1148.30) | 2.53(2.01,3.05) | 64.57(58.54,71.07) | 66.46(62.07,71.08) | -0.45(-0.66,-0.24) |
| Saint Kitts and Nevis | male | 3.65(2.61,4.93) | 6.39(4.67,8.62) | 2.09(2.00,2.19) | 90.71(22.75,243.18) | 144.28(55.03,308.11) | 1.47(1.41,1.53) |
| Saint Lucia | male | 17.42(12.46,23.12) | 28.67(20.98,38.53) | 1.60(1.41,1.78) | 124.19(72.87,198.07) | 203.70(136.02,293.74) | 1.43(1.31,1.55) |
| Saint Vincent and the Grenadines | male | 12.30(8.75,16.70) | 17.72(13.11,23.01) | 1.22(1.18,1.25) | 105.35(54.91,183.39) | 188.38(111.12,299.15) | 2.08(2.02,2.14) |
| Samoa | male | 39.91(30.71,51.73) | 76.69(59.03,98.18) | 1.99(1.77,2.21) | 208.89(148.97,285.32) | 363.33(286.23,455.23) | 1.83(1.73,1.92) |
| San Marino | male | 0.73(0.39,1.12) | 1.98(1.28,2.80) | 3.61(3.37,3.85) | 36.89(0.28,269.47) | 95.24(11.37,349.97) | 3.42(3.38,3.46) |
| Sao Tome and Principe | male | 4.25(2.71,6.13) | 13.58(9.24,19.14) | 3.94(3.80,4.08) | 36.74(10.45,93.53) | 66.08(35.68,112.29) | 2.06(2.01,2.10) |
| Saudi Arabia | male | 1447.59(1027.83,1928.25) | 6539.18(4729.46,8983.32) | 6.23(5.81,6.65) | 81.91(77.74,86.24) | 186.83(182.29,191.46) | 3.42(3.13,3.71) |
| Senegal | male | 358.87(239.68,516.07) | 1347.09(922.41,1821.30) | 4.64(4.19,5.10) | 53.16(47.78,58.99) | 86.03(81.48,90.76) | 1.83(1.42,2.23) |
| Serbia | male | 367.83(244.79,535.66) | 434.29(282.22,642.78) | 0.07(-0.16,0.30) | 54.28(48.87,60.12) | 78.03(70.86,85.74) | 1.19(1.16,1.23) |
| Seychelles | male | 4.86(3.49,6.57) | 11.29(8.07,15.81) | 3.21(3.07,3.35) | 67.34(21.42,159.09) | 146.51(73.77,262.89) | 2.71(2.67,2.76) |
| Sierra Leone | male | 54.94(30.60,82.91) | 294.13(200.35,410.73) | 6.97(6.57,7.37) | 17.96(13.52,23.40) | 35.07(31.17,39.32) | 2.45(2.37,2.52) |
| Singapore | male | 188.44(130.58,260.78) | 334.56(233.73,464.07) | 2.32(1.86,2.79) | 63.41(54.66,73.18) | 123.95(111.02,137.97) | 1.75(1.48,2.02) |
| Slovakia | male | 173.24(113.44,251.50) | 195.59(122.05,295.15) | 0.26(-0.22,0.74) | 43.66(37.40,50.68) | 64.30(55.58,74.05) | 1.25(1.18,1.32) |
| Slovenia | male | 67.56(45.67,95.88) | 69.89(44.03,103.22) | 0.01(-0.34,0.36) | 44.75(34.71,56.83) | 68.56(53.42,86.78) | 1.44(1.37,1.50) |
| Solomon Islands | male | 73.58(57.02,92.58) | 249.61(197.46,310.75) | 4.03(3.90,4.17) | 213.92(167.65,269.26) | 397.42(349.57,450.08) | 2.21(2.16,2.26) |
| Somalia | male | 377.97(269.32,507.46) | 1967.21(1440.69,2533.91) | 4.99(4.51,5.47) | 59.30(53.30,65.83) | 91.47(87.46,95.62) | 1.47(1.36,1.58) |
| South Africa | male | 1751.86(1172.74,2460.57) | 3556.39(2550.60,4761.32) | 2.75(2.33,3.18) | 47.63(45.42,49.91) | 75.53(73.07,78.06) | 1.67(1.56,1.79) |
| South Sudan | male | 328.13(231.73,436.47) | 711.68(516.45,937.89) | 2.66(2.35,2.97) | 50.42(45.10,56.19) | 75.87(70.30,81.78) | 1.47(1.41,1.53) |
| Spain | male | 1464.27(903.49,2074.50) | 2636.97(1663.25,3775.99) | 1.38(0.95,1.81) | 43.74(41.53,46.04) | 114.90(110.56,119.37) | 3.00(2.67,3.32) |
| Sri Lanka | male | 1035.26(757.47,1364.52) | 2051.50(1506.12,2738.78) | 1.91(1.56,2.26) | 62.35(58.61,66.27) | 123.96(118.65,129.44) | 2.12(1.89,2.35) |
| Sudan | male | 1133.27(809.29,1544.77) | 5457.20(3879.03,7433.11) | 5.70(5.59,5.80) | 60.38(56.90,64.02) | 128.03(124.65,131.47) | 2.90(2.77,3.03) |
| Suriname | male | 47.84(34.87,63.79) | 94.76(70.88,124.40) | 3.04(2.81,3.28) | 113.51(83.63,150.77) | 202.97(164.17,248.21) | 2.36(2.21,2.51) |
| Sweden | male | 280.52(126.59,455.45) | 546.42(332.32,791.84) | 2.74(2.18,3.31) | 46.27(41.00,52.03) | 92.46(84.86,100.55) | 2.29(1.85,2.73) |
| Switzerland | male | 183.94(99.67,291.28) | 432.13(274.16,620.71) | 3.21(2.81,3.60) | 35.54(30.54,41.16) | 86.21(78.24,94.81) | 2.82(2.50,3.14) |
| Syrian Arab Republic | male | 1021.86(742.01,1376.82) | 1793.80(1276.93,2416.15) | 0.99(-0.01,2.01) | 79.43(74.62,84.48) | 126.14(120.29,132.20) | 1.35(1.25,1.44) |
| Taiwan (Province of China) | male | 1728.14(1290.02,2232.75) | 1469.06(1084.82,1964.00) | -0.81(-1.26,-0.35) | 89.78(85.59,94.13) | 98.78(93.76,104.02) | 0.11(-0.27,0.50) |
| Tajikistan | male | 314.86(221.02,425.73) | 928.43(662.30,1266.94) | 4.28(4.04,4.51) | 62.17(55.49,69.45) | 102.61(96.12,109.44) | 1.69(1.65,1.73) |
| Thailand | male | 3206.17(2338.54,4270.82) | 4072.17(2919.22,5560.24) | -0.07(-0.42,0.28) | 52.50(50.70,54.35) | 85.11(82.51,87.78) | 0.76(0.42,1.10) |
| Timor-Leste | male | 37.07(27.68,47.75) | 126.32(95.05,163.17) | 4.20(3.92,4.49) | 47.98(33.79,66.13) | 88.13(73.38,105.02) | 1.98(1.89,2.06) |
| Togo | male | 84.05(50.24,121.97) | 322.20(215.19,444.42) | 4.45(4.31,4.59) | 24.65(19.64,30.56) | 42.34(37.83,47.24) | 1.68(1.57,1.79) |
| Tokelau | male | 0.27(0.21,0.35) | 0.40(0.31,0.52) | 1.39(1.16,1.62) | 187.42(0.00,3173.25) | 352.36(0.06,4118.86) | 2.26(2.19,2.33) |
| Tonga | male | 18.02(13.85,23.37) | 30.30(23.39,38.87) | 1.74(1.62,1.86) | 176.02(104.01,280.01) | 318.31(214.96,454.89) | 2.06(1.98,2.15) |
| Trinidad and Tobago | male | 108.71(77.30,145.56) | 148.66(107.07,201.69) | 1.32(0.69,1.95) | 100.16(82.21,120.88) | 163.79(138.52,192.37) | 1.92(1.73,2.11) |
| Tunisia | male | 561.32(390.21,770.56) | 1244.36(876.37,1690.98) | 2.84(2.53,3.15) | 65.02(59.75,70.63) | 151.97(143.65,160.66) | 3.02(2.95,3.09) |
| Turkey | male | 2888.97(1983.47,4055.38) | 6025.96(4194.34,8094.87) | 3.14(2.87,3.41) | 48.32(46.58,50.12) | 86.22(84.06,88.43) | 2.75(2.42,3.08) |
| Turkmenistan | male | 199.65(138.47,276.12) | 468.68(331.18,635.37) | 3.34(2.97,3.72) | 55.45(48.02,63.72) | 107.51(97.99,117.73) | 2.35(2.24,2.45) |
| Tuvalu | male | 1.58(1.20,2.05) | 4.44(3.40,5.66) | 4.08(3.92,4.25) | 203.08(16.31,841.16) | 378.32(112.36,928.38) | 2.26(2.20,2.32) |
| Uganda | male | 1031.90(736.92,1388.94) | 4306.69(3185.84,5681.41) | 5.00(4.81,5.20) | 63.55(59.72,67.56) | 102.15(99.11,105.27) | 1.59(1.42,1.76) |
| Ukraine | male | 2130.70(1494.50,2880.50) | 1644.74(1083.89,2241.93) | -1.06(-1.67,-0.44) | 59.56(57.06,62.14) | 72.56(69.09,76.17) | 0.27(0.07,0.46) |
| United Arab Emirates | male | 110.25(74.51,156.23) | 451.02(315.59,625.57) | 6.38(3.86,8.95) | 61.73(50.47,75.01) | 130.10(118.37,142.68) | 2.57(2.40,2.74) |
| United Kingdom | male | 2663.21(1591.52,3992.16) | 13004.81(9422.91,17603.59) | 8.08(6.81,9.38) | 61.94(59.60,64.35) | 339.95(334.12,345.86) | 7.70(6.52,8.88) |
| United Republic of Tanzania | male | 922.18(639.00,1258.90) | 3487.02(2550.00,4623.88) | 4.30(4.10,4.50) | 38.90(36.41,41.51) | 65.99(63.81,68.22) | 1.65(1.52,1.78) |
| United States of America | male | 6918.96(2869.45,11662.96) | 12468.76(7044.51,19050.59) | 3.00(2.63,3.38) | 35.68(34.84,36.53) | 56.67(55.68,57.68) | 2.33(2.03,2.63) |
| United States Virgin Islands | male | 7.37(5.44,9.73) | 10.36(7.57,13.71) | 1.06(0.99,1.13) | 87.75(36.10,180.00) | 170.34(82.86,311.19) | 2.09(1.98,2.20) |
| Uruguay | male | 25.76(10.46,43.57) | 108.55(66.66,160.51) | 5.25(4.55,5.94) | 10.58(6.89,15.54) | 41.77(34.28,50.43) | 5.15(4.40,5.91) |
| Uzbekistan | male | 1141.52(786.50,1571.19) | 2858.15(2034.71,3867.85) | 3.34(2.81,3.87) | 57.59(54.29,61.03) | 94.79(91.35,98.34) | 1.47(1.12,1.82) |
| Vanuatu | male | 28.43(21.77,36.16) | 97.63(75.27,123.93) | 4.62(4.51,4.73) | 202.42(134.86,292.40) | 355.90(288.79,434.08) | 2.09(2.04,2.15) |
| Venezuela (Bolivarian Republic of) | male | 1521.79(1126.61,1995.59) | 2824.30(2125.17,3712.36) | 2.52(2.15,2.90) | 81.23(77.20,85.42) | 133.54(128.66,138.56) | 1.59(1.39,1.79) |
| Viet Nam | male | 2613.92(1890.49,3413.19) | 4802.38(3579.30,6337.62) | 2.45(2.22,2.68) | 40.14(38.61,41.71) | 65.78(63.93,67.68) | 1.65(1.29,2.02) |
| Yemen | male | 487.23(331.77,685.26) | 2499.03(1757.26,3345.69) | 4.96(4.20,5.73) | 43.81(39.97,47.93) | 83.88(80.62,87.25) | 1.66(0.77,2.56) |
| Zambia | male | 484.19(343.95,642.30) | 1660.15(1221.68,2222.29) | 4.34(4.28,4.40) | 60.56(55.26,66.24) | 90.36(86.06,94.82) | 1.32(1.14,1.51) |
| Zimbabwe | male | 589.57(415.52,790.22) | 1462.33(1058.40,2020.27) | 2.69(2.13,3.25) | 56.92(52.39,61.74) | 99.72(94.66,104.99) | 1.91(1.47,2.34) |

**Supplementary table5.The top three and the bottom three countries of type 2 diabetes among adolescents incidence, or DALY.**

| Measure | sex | Top three countries | | | Bottom three countries | | |
| --- | --- | --- | --- | --- | --- | --- | --- |
| 2019 ASR (per 100,000 people) |  |  |  |  |  |  |  |
| Age-standardized DALY rate |  |  |  |  |  |  |  |
|  | both | Marshall Islands(207.13) | Solomon Islands(261.18) | Kiribati(277.42) | Canada(3.76) | Australia(4.88) | Belarus(5.59) |
|  | female | Fiji(249.40) | Kiribati(252.78) | Solomon Islands(288.20) | Canada(4.98) | Australia(6.25) | Belarus(6.37) |
|  | male | Zimbabwe(215.77) | Solomon Islands(236.00) | Kiribati(303.18) | Canada(2.60) | France(3.15) | Australia(3.55) |
| ASIR |  |  |  |  |  |  |  |
|  | both | Niue(531.10) | American Samoa(623.60) | Marshall Islands(651.16) | Canada(22.00) | Greenland(26.45) | Australia(34.72) |
|  | female | Niue(425.08) | American Samoa(545.83) | Marshall Islands(578.32) | Canada(25.51) | Greenland(27.32) | Nigeria(36.04) |
|  | male | Niue(629.89) | American Samoa(699.65) | Marshall Islands(721.37) | Canada(18.65) | Greenland(25.63) | Australia(31.51) |
| 1990-2019 increase times |  |  |  |  |  |  |  |
| DALY (cases) |  |  |  |  |  |  |  |
|  | both | Sierra Leone(4.89) | Guatemala(5.77) | Qatar(6.90) | Belarus(0.31) | Bulgaria(0.40) | Romania(0.41) |
|  | female | Cameroon(5.08) | Guatemala(5.62) | Sierra Leone(6.10) | Belarus(0.27) | Romania(0.35) | Bulgaria(0.38) |
|  | male | Afghanistan(5.03) | Guatemala(6.01) | Qatar(9.32) | Belarus(0.39) | Bulgaria(0.45) | Republic of Moldova(0.47) |
| Incidence (case) |  |  |  |  |  |  |  |
|  | both | Afghanistan(6.87) | Niger(7.32) | Qatar(12.47) | Latvia(0.54) | Belarus(0.63) | Ukraine(0.68) |
|  | female | Afghanistan(6.50) | Niger(7.01) | Qatar(7.09) | Latvia(0.57) | Belarus(0.58) | Ukraine(0.60) |
|  | male | Afghanistan(7.59) | Niger(7.73) | Qatar(15.14) | Latvia(0.52) | Belarus(0.69) | Estonia(0.70) |
| EAPC |  |  |  |  |  |  |  |
| DALY |  |  |  |  |  |  |  |
|  | both | Mauritius(4.21) | United States of America(4.73) | United Kingdom(5.96) | Ethiopia(-3.32) | Belarus(-3.25) | Philippines(-3.06) |
|  | female | Mauritius(4.11) | Finland(4.16) | United Kingdom(5.96) | Belarus(-3.76) | Ethiopia(-3.40) | Greenland(-2.85) |
|  | male | Singapore(4.66) | United Kingdom(5.91) | United States of America(6.10) | Philippines(-4.15) | Ethiopia(-3.24) | Rwanda(-2.71) |
| Incidence |  |  |  |  |  |  |  |
|  | both | Uruguay(4.41) | Greenland(4.98) | United Kingdom(7.72) | Ethiopia(-1.04) | Rwanda(-0.74) | Belarus(-0.41) |
|  | female | Spain(4.30) | Greenland(5.33) | United Kingdom(7.71) | Ethiopia(-1.45) | Rwanda(-1.06) | Belarus(-0.72) |
|  | male | Greenland(4.69) | Uruguay(5.15) | United Kingdom(7.70) | Philippines(-1.09) | Ethiopia(-0.60) | Rwanda(-0.45) |

**Supplementary tables 6. The top three and the bottom three regions of type 2 diabetes among adolescents incidence, or DALY.**

| Measure | sex | Top three regions | | | Bottom three regions | | |
| --- | --- | --- | --- | --- | --- | --- | --- |
| 2019 ASR (per 100,000 people) |  |  |  |  |  |  |  |
| Age-standardized DALY rate |  |  |  |  |  |  |  |
|  | both | Central Latin America(88.66) | Caribbean(112.77) | Oceania(183.20) | Australasia(5.98) | Eastern Europe(9.60) | Central Europe(12.84) |
|  | female | Central Latin America(100.48) | Caribbean(151.41) | Oceania(163.94) | Australasia(7.74) | Eastern Europe(9.38) | Central Europe(13.78) |
|  | male | Central Latin America(76.84) | Southern sub-Saharan Africa(101.85) | Oceania(201.49) | Australasia(4.29) | Southern Latin America(9.17) | Eastern Europe(9.81) |
| ASIR |  |  |  |  |  |  |  |
|  | both | Caribbean(166.77) | Central Latin America(179.29) | Oceania(374.88) | Australasia(36.32) | Western sub-Saharan Africa(50.58) | Eastern Europe(51.26) |
|  | female | Central Latin America(190.28) | Caribbean(193.42) | Oceania(301.69) | Australasia(40.08) | Eastern Europe(48.95) | Western sub-Saharan Africa(50.75) |
|  | male | East Asia(165.01) | Central Latin America(168.37) | Oceania(442.52) | Australasia(32.70) | Western sub-Saharan Africa(50.41) | High-income North America(53.31) |
| 1990-2019 increase times |  |  |  |  |  |  |  |
| DALY (cases) |  |  |  |  |  |  |  |
|  | both | Western sub-Saharan Africa(3.08) | Oceania(3.11) | High-income North America(3.91) | Eastern Europe(0.53) | Central Europe(0.70) | East Asia(0.76) |
|  | female | Oceania(3.09) | Western sub-Saharan Africa(3.16) | High-income North America(3.42) | Eastern Europe(0.46) | Central Europe(0.62) | East Asia(0.64) |
|  | male | Oceania(3.12) | Southern Latin America(3.57) | High-income North America(4.75) | Eastern Europe(0.62) | Central Europe(0.81) | East Asia(0.86) |
| Incidence (case) |  |  |  |  |  |  |  |
|  | both | Oceania(3.36) | Central sub-Saharan Africa(3.82) | Western sub-Saharan Africa(3.96) | Eastern Europe(0.73) | East Asia(0.92) | Central Europe(1.01) |
|  | female | Oceania(3.43) | Central sub-Saharan Africa(3.77) | Western sub-Saharan Africa(3.78) | Eastern Europe(0.68) | East Asia(0.78) | Central Europe(0.99) |
|  | male | North Africa and Middle East(3.41) | Central sub-Saharan Africa(3.87) | Western sub-Saharan Africa(4.17) | Eastern Europe(0.77) | East Asia(1.01) | Central Europe(1.03) |
| EAPC |  |  |  |  |  |  |  |
| DALY |  |  |  |  |  |  |  |
|  | both | East Asia(2.81) | Western Europe(3.30) | High-income North America(4.72) | Eastern Europe(-1.47) | Tropical Latin America(-1.19) | Eastern Sub-Saharan Africa(-0.87) |
|  | female | East Asia(2.13) | Western Europe(3.48) | High-income North America(3.81) | Eastern Europe(-1.97) | Tropical Latin America(-1.96) | Eastern Sub-Saharan Africa(-1.18) |
|  | male | Southern Latin America(3.29) | East Asia(3.34) | High-income North America(6.06) | Eastern Europe(-0.94) | Eastern Sub-Saharan Africa(-0.59) | Southeast Asia(-0.47) |
| Incidence |  |  |  |  |  |  |  |
|  | both | Southern Latin America(3.26) | East Asia(3.56) | Western Europe(4.15) | Eastern Europe(-0.06) | Eastern Sub-Saharan Africa(0.65) | Southeast Asia(0.68) |
|  | female | East Asia(3.07) | Southern Latin America(3.10) | Western Europe(4.27) | Eastern Europe(-0.31) | Eastern Sub-Saharan Africa(0.31) | Southeast Asia(0.58) |
|  | male | Southern Latin America(3.48) | East Asia(3.89) | Western Europe(4.03) | Eastern Europe(0.16) | Southeast Asia(0.79) | Eastern Sub-Saharan Africa(0.95) |
