# Supplementary material for: Global burden of type 2 diabetes in adolescents from 1990 to 2019
Source: Front Endocrinol (Lausanne). 2024 Jul 11;15:1405739. doi: 10.3389/fendo.2024.1405739 (PMC11269148; doi:10.3389/fendo.2024.1405739)
Supplement: Supplementary file 2 [file DataSheet_2.docx]

**Supplemental figures**


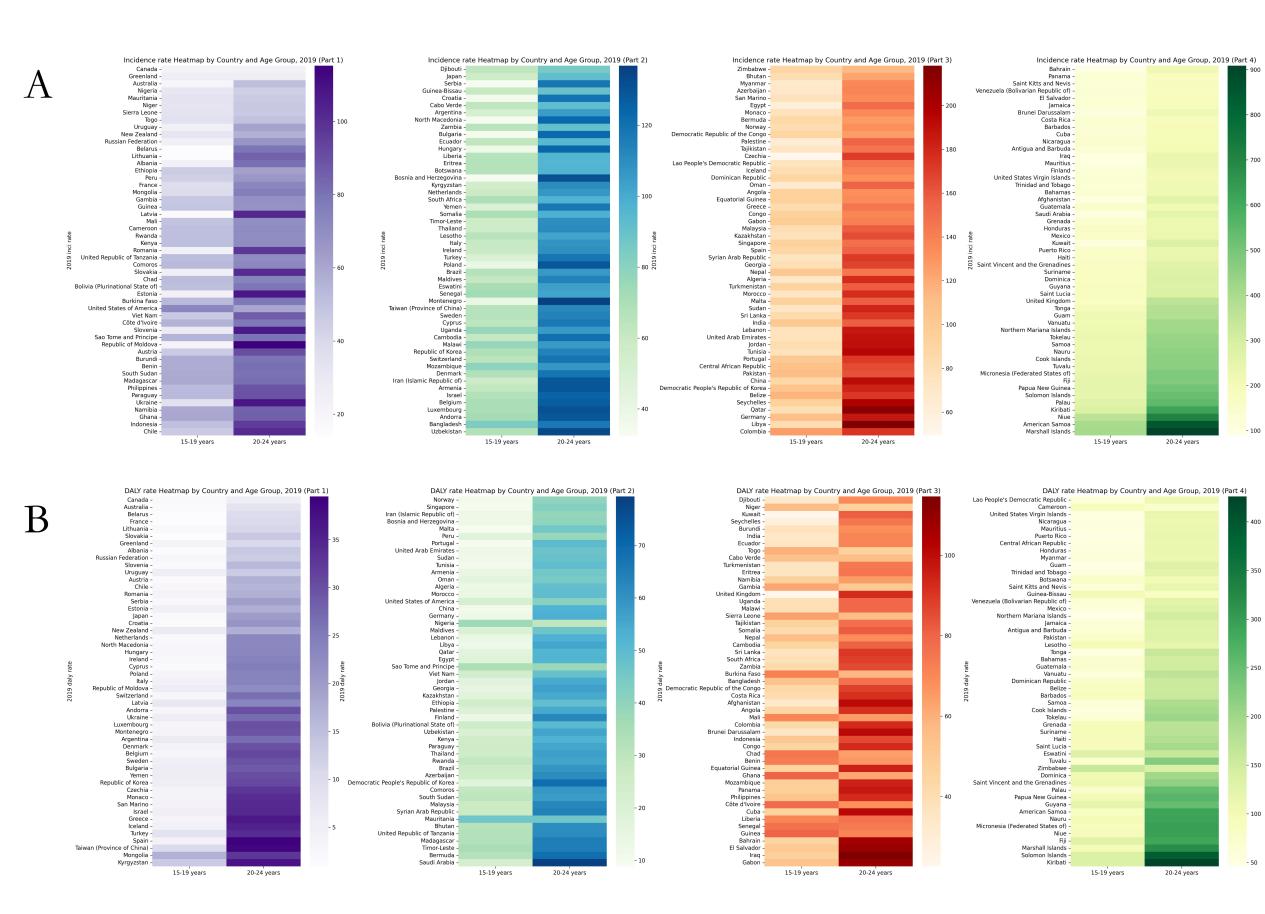


Supplementary figure 1(A) The incidence rate Heat map by Country and Age Group in 2019. (B) The DALY rate Heat map by Country and Age Group in 2019.


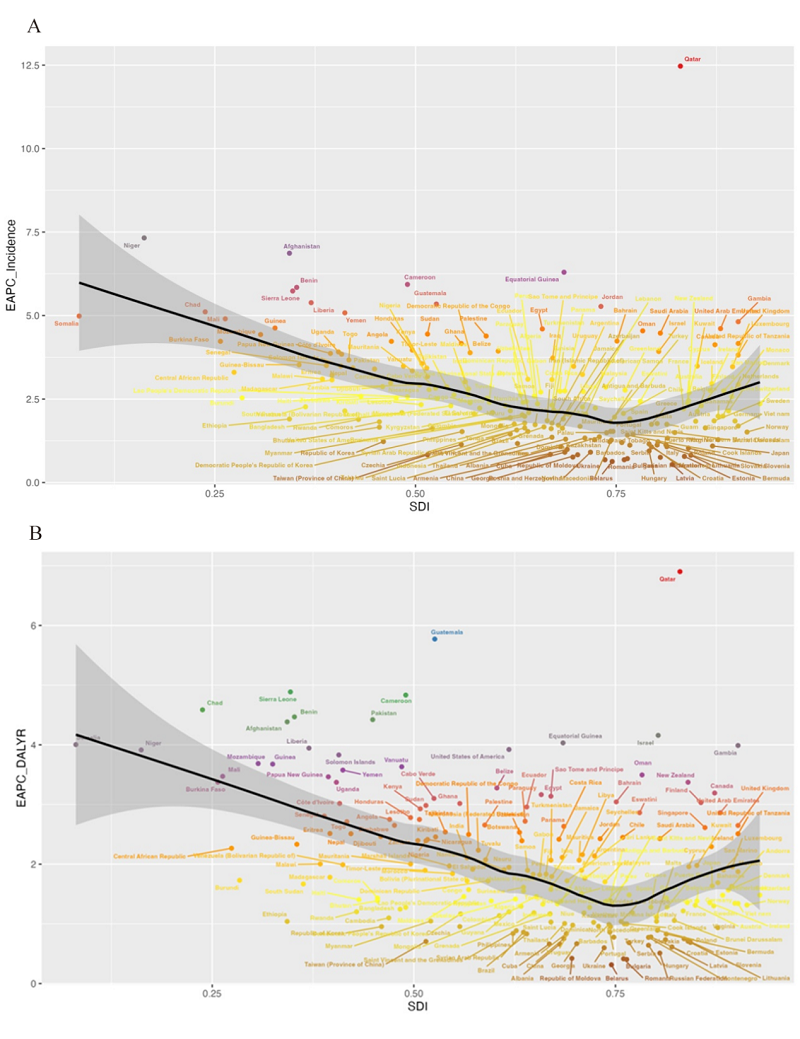


Supplementary figure 2(A) The relationship between ASIR and EAPC and SDI in 204 countries. (B) The relationship between DALY and EAPC and SDI in 204 countries.


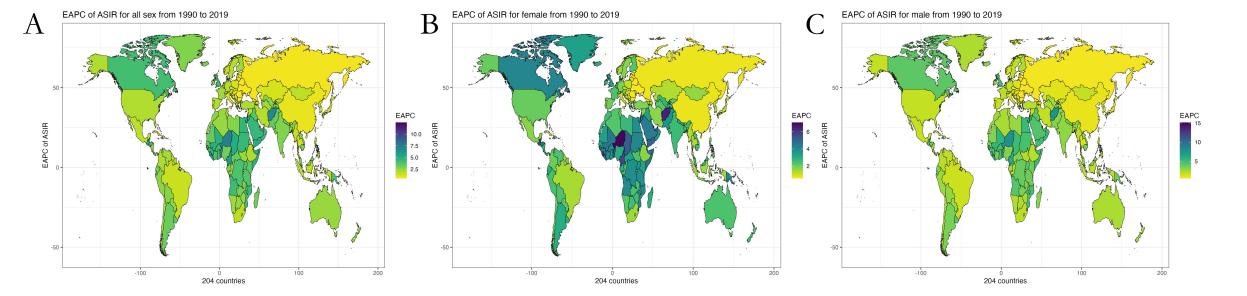


Supplementary figure 3(A)The EAPC of ASIR for all sex from 1990 to 2019. (B) The EAPC of ASIR for male from 1990 to 2019. (C) The EAPC of ASIR for female from 1990 to 2019


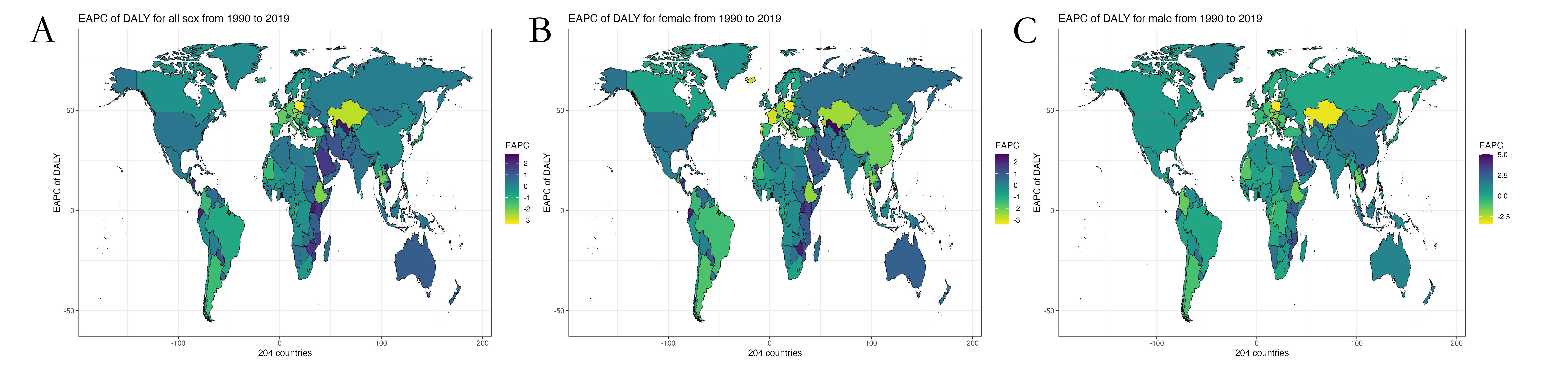


Supplementary figure 4(A) The EAPC of DALY for all sex from 1990 to 2019. (B) The EAPC of DALY for male from 1990 to 2019. (C) The EAPC of DALY for female from 1990 to 2019
